# High-risk neuroblastoma with NF1 loss of function is targetable using SHP2 inhibition

**DOI:** 10.1016/j.celrep.2022.111095

**Published:** 2022-07-26

**Authors:** Jinyang Cai, Sheeba Jacob, Richard Kurupi, Krista M. Dalton, Colin Coon, Patricia Greninger, Regina K. Egan, Giovanna T. Stein, Ellen Murchie, Joseph McClanaghan, Yuta Adachi, Kentaro Hirade, Mikhail Dozmorov, John Glod, Sosipatros A. Boikos, Hiromichi Ebi, Huaixiang Hao, Giordano Caponigro, Cyril H. Benes, Anthony C. Faber

**Affiliations:** 1 Philips Institute for Oral Health Research, School of Dentistry, and Massey Cancer Center, Virginia Commonwealth University, Richmond, VA 23298, USA; 2 Center for Cancer Research, Massachusetts General Hospital, Harvard Medical School, Charlestown, MA, USA; 3 Division of Molecular Therapeutics, Aichi Cancer Center Research Institute, Nagoya, Aichi 464-8681, Japan; 4 Department of Biostatistics, Virginia Commonwealth University, Richmond, VA 23298, USA; 5 National Cancer Institute, Pediatric Branch, Oncology, Bethesda, MD, USA; 6 Department of Medicine, Virginia Commonwealth University, Richmond, VA 23298, USA; 7 Novartis Institute for Biological Research, 250 Massachusetts Avenue, Cambridge, MA 02139, USA; 8 These authors contributed equally; 9 Present address: Novartis Institute of Biological Research, 10675 John Jay Hopkins Dr, San Diego, CA 92121, USA; 10 Lead contact

## Abstract

Reoccurring/high-risk neuroblastoma (NB) tumors have the enrichment of non-RAS/RAF mutations along the mitogen-activated protein kinase (MAPK) signaling pathway, suggesting that activation of MEK/ERK is critical for their survival. However, based on preclinical data, MEK inhibitors are unlikely to be active in NB and have demonstrated dose-limiting toxicities that limit their use. Here, we explore an alternative way to target the MAPK pathway in high-risk NB. We find that NB models are among the most sensitive among over 900 tumor-derived cell lines to the allosteric SHP2 inhibitor SHP099. Sensitivity to SHP099 in NB is greater in models with loss or low expression of the RAS GTPase activation protein (GAP) neurofibromin 1 (NF1). Furthermore, NF1 is lower in advanced and relapsed NB and NF1 loss is enriched in high-risk NB tumors regardless of MYCN status. SHP2 inhibition consistently blocks tumor growth in high-risk NB mouse models, revealing a new drug target in relapsed NB.

## In brief

In this paper, Cai et al. demonstrate that high-risk neuroblastomas with low NF1 expression are sensitive to SHP2 inhibitors, which may have treatment advantages over MEK inhibitors. Targeting SHP2 blocks neuroblastoma tumor growth. As several SHP2 inhibitors are in clinical trials, SHP2 inhibitors may benefit high-risk NB patients.

## Graphical Abstract

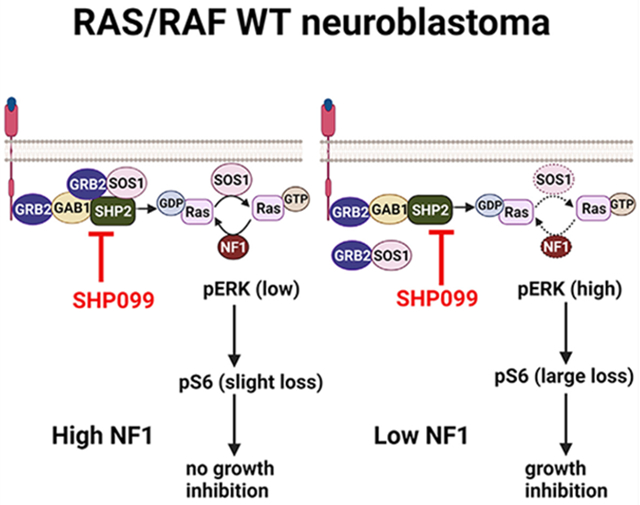

## INTRODUCTION

Neuroblastoma (NB) is a pediatric cancer developing from undifferentiated neural crest cells along the sympathetic nervous system (Cooper et al., 1990). Effective maintenance therapy has helped improve the outcomes of high-risk NB patients following multi-agent chemotherapy (platinum, alkylating, and topoisomerase agents) and myeloablative consolidation (Sano et al., 2019b). Maintenance therapy consists of high-dose 13-cis retinoic acid (RA) (isotretinoin) (Matthay et al., 2009); in addition, the inclusion of the anti-GD2 antibody dinutuximab with interleukin-2 (IL-2) and granulocyte macrophage-colony-stimulating factor (GM-CSF) during maintenance therapy improves outcomes in some patients (McGinty and Kolesar, 2017; Sano et al., 2019b; Yu et al., 2010). Still, half of all high-risk patients succumb to their disease, and NB accounts for the most cancer-related deaths in children aged 5 years and younger (Gao et al., 1997). While there have been intentional investigations into new therapies for NB, including the dedicated new approaches to NB therapy (Morris et al., 2013) consortium, major breakthroughs significantly altering the fate of high-risk NBs have been elusive.

Overall, NB tumors do not have a high percentage of mutations that would make them amenable to current targeted molecular agents that block growth factor pathways. However, recent studies, particularly focused on reoccurring NB tumors, have demonstrated a potentially critical role of the mitogen-activated protein kinase/extracellular signal-regulated kinase (MAPK/ERK) pathway. In fact, anaplastic lymphoma kinase (ALK) and downstream mutations that activate the MAPK/ERK pathway (Lopez-Delisle et al., 2018) are found in 80% of reoccurring NB tumors (Eleveld et al., 2015; Padovan-Merhar et al., 2016). Further supporting the role of the MEK/ERK pathway in high-risk/refractory NB, elegant studies in zebrafish models of NB have demonstrated that the expression of GAB2 or src homology region 2 domain phosphatase (SHP2) (positive regulators of the RAS-MAPK pathway) (Zhang et al., 2017) or loss of the RAS-GAP NF1 (He et al., 2016) potentiate *MYCN*-amplification-driven NB tumorigenesis.

Despite the accumulating evidence of an important role for the MAPK/ERK pathway in NB tumorigenesis, maintenance, and in particular, chemotherapy resistance/progression, preclinical studies with US Food and Drug Administration (FDA)-approved MEK inhibitors such as trametinib have not demonstrated wide-spread activity outside of *RAS* mutant NB cell lines, including a lack of activity against *ALK* mutant and *MYCN*-amplified NB (Hart et al., 2017; Umapathy et al., 2017). Importantly, the RAS-MAPK pathway is controlled by a plethora of inputs, and many different positive and negative feedback loops in the pathway have been shown to limit the activity of single-agent MEK inhibitors in other contexts (Lito et al., 2012; Montero-Conde et al., 2013; Turke et al., 2012). In addition, while MEK inhibitors are in clinical use in combination with RAF inhibitors in *BRAF* mutant cancers, they have demonstrated significant and frequent dose-limiting toxicities (DLTs), including severe gastrointestinal, cardiological, ocular, and liver toxicities (Brachmann et al., 2005; Feoktistova et al., 2016; Harenza et al., 2017; Lhuissier et al., 2017; Mourad et al., 2019; Rinehart et al., 2004; Welsh and Corrie, 2015)—when pERK levels have been measured in patients on MEK inhibitor treatment, the amount of inhibition is variable (Infante et al., 2012; Rinehart et al., 2004), and usually less than 50% in *RAS/RAF* wild-type (WT) tumors (Infante et al., 2012), speaking to the limited therapeutic window for these cancers.

SHP099 (Chen et al., 2016) is a newly developed allosteric inhibitor of SHP2, emerging as the first highly specific tyrosine phosphatase inhibitor of any kind. SHP099 stabilizes the closed, repressed conformation of SHP2 (Ran et al., 2016). Thus, SHP099 inhibits both scaffolding roles of SHP2 by blocking the access to SH2 domains for p-Tyr (phosphotyrosine) and catalytic activity of SHP2. SHP099 has demonstrated preclinical activity in some receptor tyrosine kinase (RTK)-driven cancers, alone (Chen et al., 2016) and in rational combinations (Dardaei et al., 2018). The initial report on the preclinical activity of SHP099 (Chen et al., 2016) included profiling across 250 cell lines with several subtypes of cancer, but this study was focused on adult cancers (Chen et al., 2016). Follow-up-focused studies demonstrated that various subtypes of cancers may be susceptible to SHP2 inhibition, usually in rational combinations: In a recent study, we demonstrated that SHP2 inhibition can counter the acquired resistance to ALK inhibitors by blocking signaling downstream of several RTKs engaged in compensatory signaling limiting the activity of ALK inhibitors (Dardaei et al., 2018), and studies have shown that SHP2 inhibition can potentiate the effect of MEK inhibitors in *KRAS*-WT gastroesophageal cancers (Wong et al., 2018), as well as restrict adaptive resistance to MEK inhibitors in *KRAS*-mutant cancers (Ahmed et al., 2019; Fedele et al., 2018; Ruess et al., 2018). In all of the cases, SHP099 effects are attributed to the inhibition of the MEK/ERK pathway (Ahmed et al., 2019; Chen et al., 2016; Dardaei et al., 2018; Fedele et al., 2018; Nichols et al., 2018; Ruess et al., 2018; Wong et al., 2018); importantly, a related molecule is in clinical trials (NCT03114319).

## RESULTS

### SHP099 is effective in a subset of neuroblastoma

**Figure 1.**
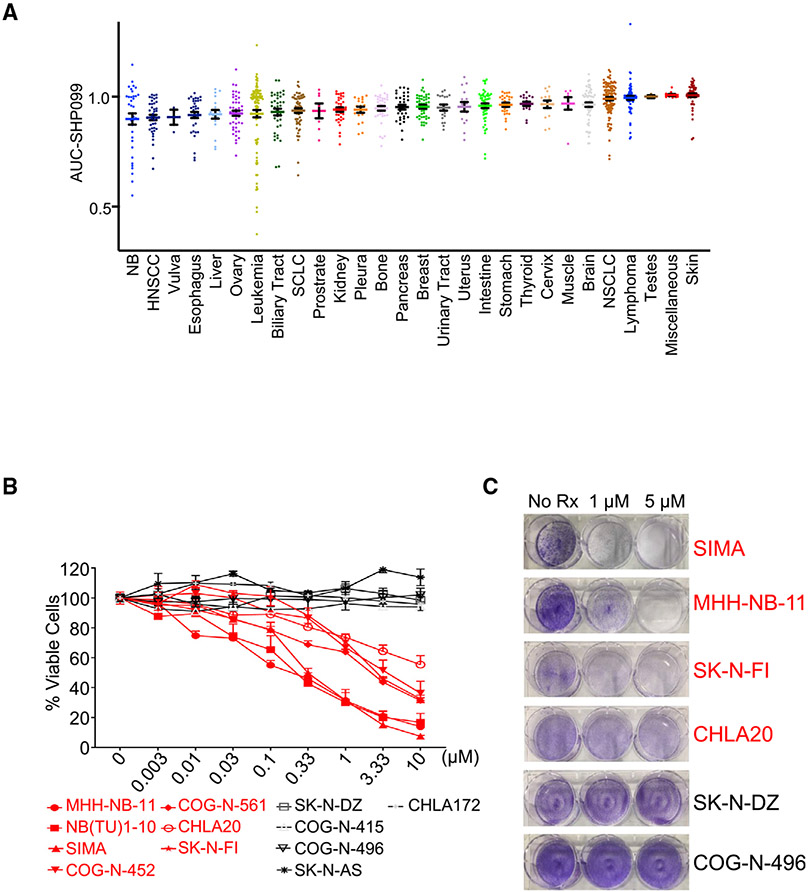
SHP099 sensitivity in neuroblastoma cell lines (A) Activity of SHP099 across 922 cell lines from 28 tissue types, including 32 NB cell lines. (B) Graph represents percentage of viable cells assessed by CellTiter-Glo in the NB cell lines indicated, following 7-day treatment with increasing concentrations (0.003–10 μM) of SHP099. SHP099-sensitive lines are shown in red, and SHP099-resistant lines are shown in black. Three individual sets of experiments were performed to calculate significance. Error bars are ± SEMs. (C) Crystal violet staining of NB cell lines following treatment with SHP099 (1 and 5 μM) or untreated (No Rx) until "No Rx" wells reached confluency. See also Figures S1-S3 and Tables S1, S2, and S3.

To evaluate the potential of SHP2 inhibitor across tumor types, we screened 922 cancer cell lines of diverse origin with SHP099 at 9 different doses (maximum dose, 10.24 μM; lowest, 40 nM); we also concomitantly (same screen campaign) profiled trametinib and the ERK inhibitor VX11E. SHP099 displayed some expected activity on RTK-driven cell lines. For example, several *EGFR* mutant non-small cell lung cancer (NSCLC) cell lines are in the top 10% most sensitive lines overall (Figure 1A) (Chen et al., 2016). Trametinib and VX11E, but not SHP099, had substantial activity across melanoma cell lines enriched in the *BRAF* mutant as well as some mild activity in *KRAS* mutant tumors such as pancreas (Figures S1A and S1B; Table S1). These results are consistent with SHP2 acting upstream of BRAF and with *KRAS* mutant models being generally insensitive to the SHP099 single agent (see below for *KRAS* allele-specific exceptions) (Ahmed et al., 2019; Chen et al., 2016; Nichols et al., 2018; Wong et al., 2018).

Interestingly, we found an enrichment of NB in the top 10% sensitive models overall (p = 0.0006, Fisher exact test). Indeed, surprisingly, NB was the second most sensitive subset of cancer across the high-throughput screening (HTS), with leukemic lines being the most proportionally enriched in the top 10% sensitive lines (p = 0.00003, Fisher exact test; Table S1). This sensitivity of NBs to SHP099 was not noted for trametinib or VX11E. While NB has a low rate of *RAS* and *RAF* mutations (Eleveld et al., 2015), our screen did include four in total: the NB *RAS* mutant SK-N-AS and CHP-212 cells (Kiessling et al., 2016), which have some sensitivity to trametinib (Healy et al., 2020; Umapathy et al., 2017) but are resistant to SHP099 (Valencia-Sama et al., 2020; Table S2), and the *BRAF V600E* mutant cell line *ACN*, which is also sensitive to trametinib but resistant to SHP099 (Tables S1 and S2). Of note, the LAN6 cell line with a *G12C RAS* mutation that retains high intrinsic guanosine triphosphatase (GTPase) activity was an exception and sensitive to trametinib and had some, albeit modest, sensitivity to SHP099 (Tables S1 and S2). This is consistent with previous reports regarding this mutation (Fedele et al., 2018; Nichols et al., 2018). A head-to-head comparison of the hotspot *NRAS* mutation (*Q61*) highlights the vast difference between MEK inhibitor response and SHP2 inhibitor resistance (Figure S2A). We further confirmed this drug data by analyzing genetic screens with RNAi of 648 solid tumor cancer cell lines (Figure S2B), demonstrating an enhanced sensitivity to SHP2 small interfering RNA (siRNA) in the RAS/RAF WT NB cell lines, and one not appreciated in the RAS/RAF mt NB cell lines. Therefore, in general while *RAS* and *RAF* mutant NB have some sensitivity to trametinib, they are resistant to SHP099 (we refer to these cancers in this study as “type 1” NB) and a large subset of NB outside of this small genetic subset demonstrates sensitivity to SHP2 inhibition. Most are relatively resistance to MEK inhibition, which we call “type 2” NB. Below, we provide evidence that type 2 NB cells are sensitive to SHP2 inhibition based on low expression/mutation of the RAS-GAP NF1.

### *In vitro* sensitivity corroborates the SHP099 screen

To further characterize the sensitivity of NB models to SHP099, we treated a panel of NB cells and assayed for viability using both CellTiter-Glo (CTG) over 7 days (Lhuissier et al., 2017) and crystal violet-based assays over 5 to 7 days (Feoktistova et al., 2016). These experiments confirmed that a subset of *RAS/RAF* WT NB lines are very sensitive to SHP099 (Figures 1B and 1C). Furthermore, viability effects were in part due to cell death, as the sensitive cell lines, but not the resistant cell lines, underwent apoptosis as evidenced by cleaved caspase 3 and cleaved poly-ADP ribose polymerase (PARP) (Figure S3).

### Signaling changes in sensitive and resistant NBs to SHP099

**Figure 2.**
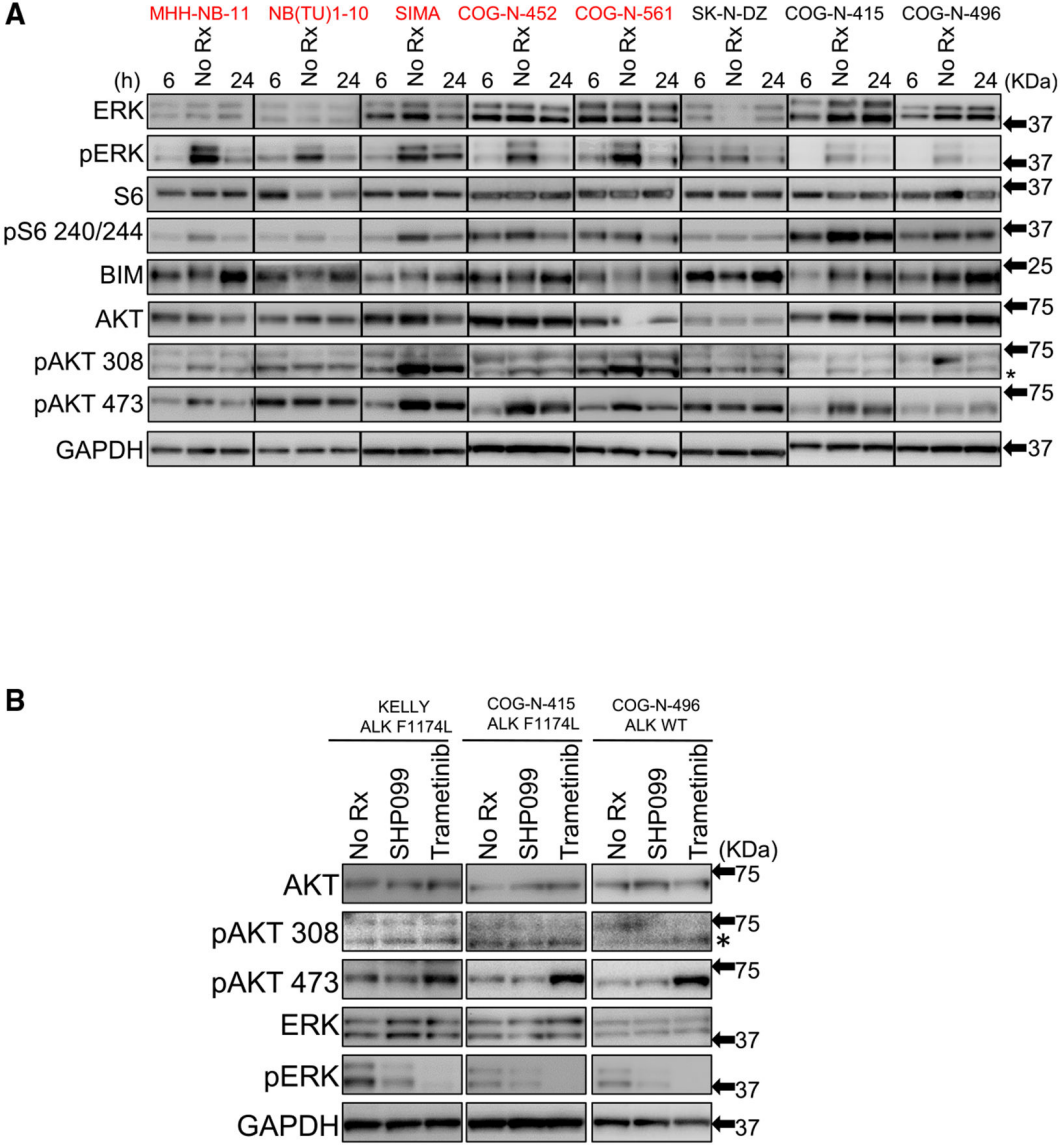
SHP099 inhibits MEK/ERK pathway in neuroblastoma cell lines (A) Immunoblot of NB cell lines indicated treated with SHP099 (5 μM) for 6 and 24 h, respectively, or untreated (No Rx) and assessed with the indicated antibodies. Glyceraldehyde 3-phosphate dehydrogenase (GAPDH) was used as a loading control. SHP099-sensitive lines are depicted in red font, and SHP099-resistant lines are depicted in black font. (B) Immunoblot of NB cell lines (*ALK* WT, *ALK F1174L* mut) treated with SHP099 (5 μM) ortrametinib (1 μM) for 24 h or untreated control (No Rx) were assessed with the indicated antibodies. See also Figures S4-S6.

We next asked whether signaling changes caused by SHP099 were different in the sensitive NB cell lines compared to insensitive NB cell lines. SHP2 primarily acts on the MEK/ERK pathway via signal activation, and we recently demonstrated that SHP099 sensitized *ALK* mutant NSCLCs to ALK inhibitors through the inhibition of pERK downstream of multiple RTKs (Dardaei et al., 2018). Here, we found that pERK was downregulated following single-agent SHP099 treatment (Figure 2A) (Dardaei et al., 2018), consistent with the hypersensitivity of NB to single-agent SHP099 in the HTS (Figure 1A; Tables S1 and S2). Interestingly, the duration and magnitude of ERK inhibition was not substantially different in the sensitive cells versus the resistant cells (Figures S4A-S4C). However, we found that baseline levels of pERK were higher in the sensitive cells (Figure S4D), providing an important clue that hyperactivation of MEK/ERK, independent of mutant *RAS/RAF*, conferred sensitivity to SHP2 inhibition. Consistent with this finding, we found that the mammalian target of rapamycin complex 1 (mTORC1) downstream of S6 protein was inhibited more potently in the sensitive cell lines (Corcoran et al., 2013; Kitai et al., 2016) (Figure S4E). This is consistent with our previous findings that mTORC1 is largely under the control of the MEK/ERK pathway in cancers “addicted” to MEK/ERK signaling (Corcoran et al., 2013; Misale et al., 2019). In addition, downregulation of the MEK/ERK pathway by SHP099 was sufficient to upregulate the BH3-only protein BIM, a major effector protein of this pathway in cancer (Faber et al., 2012; Sale and Cook, 2013) in both sensitive and resistant NBs (Figure 2A). These data are in keeping with other studies demonstrating that the MEK/ERK pathway is a critical pathway downstream of SHP2, and that high flux through the MEK/ERK pathway often includes dominant control of the mTORC1 pathway. In addition, we found that phosphatidylinositol 3-kinase (PI3K) signaling was inconsistently but measurably downregulated during the course of SHP099 treatment in some of the NBs (Figure 2A). Overall, SHP099-sensitive NBs had higher pERK signaling, and SHP099 more potently downregulated mTORC1 in these cancers.

### SHP099 treatment does not lead to feedback activation of mTORC2 substrates

We next studied why a significant subset of *RAS/RAF* WT NBs are hypersensitive to SHP2 inhibitor, yet are not hypersensitive to MEK inhibitor. MEK inhibitors have been reported to elicit unwanted feedback activation of other growth factor pathways (Lito et al., 2012; Montero-Conde et al., 2013; Turke et al., 2012), and, most pertinent to NB, it has recently been reported that NBs with *ALK* mutations are insensitive to single-agent MEK inhibitors due to MEK inhibitor-induced feedback activation of mTORC2 substrates (Umapathy et al., 2017). To evaluate whether MEK and SHP2 inhibition in NBs promoted the activation of the same mTORC2 substrates, we blotted for pAKT residue 473, which is phosphorylated directly by mTORC2, as well as pAKT 308, which is directly phosphorylated by PDK1 (Brachmann et al., 2005; Sarbassov et al., 2005). pAKT 308 was not affected by either SHP099 or trametinib, indicating that these drugs do not inhibit PI3K or the activation of AKT by PDK1. Consistent with the reported results (Umapathy et al., 2017), pAKT 473 was increased in response to trametinib in the KELLY (*ALK* mt) and COG-N-415 (*ALK* mt) cells. In addition, we found this to be true in the COG-N-496 (*ALK* WT) cells (Figure 2B). In contrast to trametinib, SHP099 did not lead to the enhanced phosphorylation of pAKT 473 in any of these cell lines (Figure 2B).

As pERK inhibition was more potent in the trametinib-treated cells, we treated SHP099-insensitive NB cells (COG-N-415 and COG-N-496) and SHP099-sensitive NB cells (SK-N-FI and COG-N-561) with SHP099 or the distinct and more potent SHP2 inhibitor RMC-4550 (Nichols et al., 2018). In parallel, we treated these cell lines with increasing concentrations of trametinib. Indeed, we found a consistent activation of mTORC2 with trametinib that was not appreciated with either SHP2 allosteric inhibitor, even when pERK suppression was equivalent to or exceeded that caused by trametinib (Figure S5A). Similar to the variable effects on pAKT, we noted in Figure 2A that pAKT was sometimes suppressed with the SHP2 inhibitors. In contrast to the activation of pAKT 473 by trametinib in the *RAS/RAF* WT NB cells (Figure S5A), we did not note the activation of mTORC2 across broad concentrations of trametinib in the *NRAS* mutant SK-N-AS cells (Figure S5B), consistent with a past finding (Umapathy et al., 2017) and with the differential sensitivity of mutant *RAS/RAF* NB and WT *RAS/RAF* NB to trametinib (Figure S1). Thus, SHP2 inhibitors do not elicit feedback activation of mTORC2 substrates, while MEK inhibitors do so in *RAS/RAF* WT NB models. Activation of mTORC2 substrates directly mitigates the efficacy of trametinib in NB (Umapathy et al., 2017), and we found that the addition of the mTORC1/2 inhibitor AZD2014 sensitized *RAS/RAF* WT NBs to trametinib (Figures S6A and S6B). Thus, the differential feedback effect contributes to the observed difference in viability outcome between MEK and SHP2 inhibition and possibility for clinical efficacy.

### Differential therapeutic window between MEK inhibition and SHP2 inhibition in RAS/RAF WT cells

We demonstrated the feedback activation of mTORC2 by MEK inhibitors but not SHP2 inhibitors in WT *RAS/RAF* NB, likely contributing to differential sensitivity between the two classes of MAPK inhibitors in these cancers. We next focused on MEK/ERK suppression, as this is the major effector pathway of both MEK inhibitors and SHP2 inhibitors. We have demonstrated that feedback mechanisms re-activate the MEK/ERK pathway following MEK inhibitors in *RAS* mutant cancers, which reduce their efficacy (Kitai et al., 2016). However, we did not note a difference in the length of pERK suppression between SHP2 and MEK inhibitors across 72 h in *RAS/RAF* WT NB (Figure S6C).

An important component to effective precision medicine is the existence of a therapeutic window caused by differences in cellular consequences of targeting a particular molecule in a cancer compared to the normal cells of a patient. We therefore focused on the magnitude of the suppression of pERK in NB cells and normal tissue-derived cells following treatment with SHP2 inhibitor or MEK inhibitor. MEK inhibitors have demonstrated diverse DLTs, including ocular and cardiac (Berger et al., 2020; Mendez-Martinez et al., 2019). A likely explanation for this is that normal cells also reportedly downregulate pERK in response to MEK inhibitor (Morris et al., 2013). Furthermore, MEK inhibitors are clinically effective primarily in *RAF* mutant cancers, where preclinical studies have found that lower concentrations of these drugs are needed to inhibit MEK/ERK signaling (Corcoran et al., 2013). To evaluate therapeutic windows for MEK inhibitor and SHP2 inhibitor, we treated the SK-N-AS *NRAS* mutant (*Q61K*) NB cells with increasing doses of trametinib and found that only 10 nM of trametinib was sufficient to abrogate pERK (Figure S5B), which aligned with approximately 70% of the cells being inhibited (Figure S2A). In contrast, 10 nM of trametinib was insufficient to block pERK signaling in the other non-*RAS/RAF* mutant NB, while neither SHP2 inhibitors reduced pERK in the *RAS* mt NB cells, but it did reduce pERK in the RAS/RAF WT NB cells, consistent with the central importance of pERK suppression for MAPK pathway inhibitors (Figure S5A).

**Figure 3.**
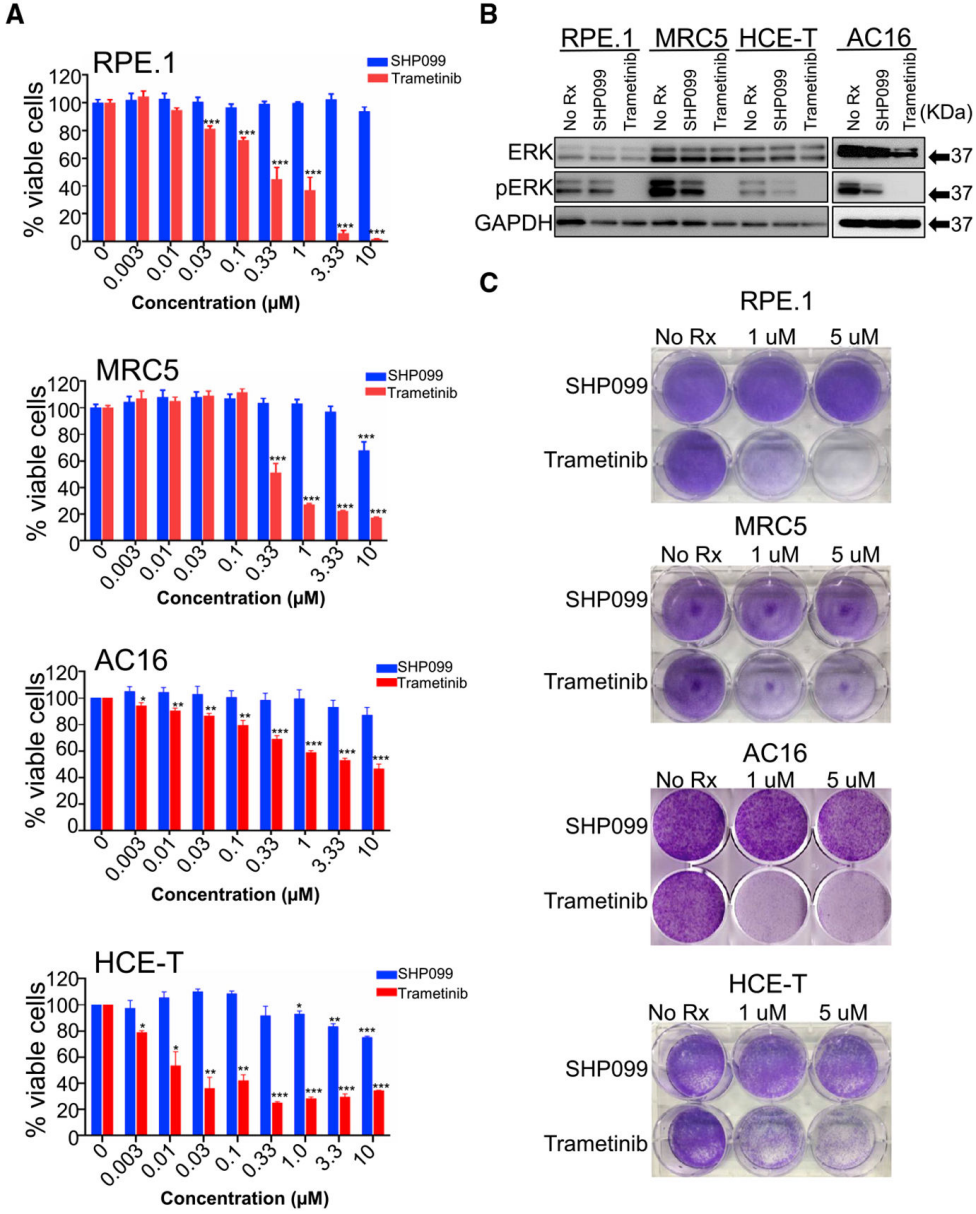
Effect of SHP099 and trametinib on normal tissue-derived cell lines (A) Graph represents percentage of viable cells assessed by CellTiter-Glo in the normal tissue-derived cell lines (RPE.1, MRC5, AC16, and HCE-T) following 7-day treatment with increasing concentrations (0.003–10 μM) of SHP099 (blue) and trametinib (red). Three individual sets of experiments were performed to calculate significance. Error bars are ± SEMs. (B) Normal tissue-derived cell lines (RPE.1, MRC5, AC16, and HCE-T) treated with SHP099 (5 μM) or trametinib (1 μM) for 24 h and untreated control (No Rx) were assessed with the indicated antibodies. GAPDH was used as a loading control. (C) Crystal violet staining of normal tissue-derived cell lines (RPE.1, MRC5, AC16, and HCE-T) following treatment with SHP099 or trametinib (concentrations are indicated) or untreated (No Rx) until "No Rx" well reached confluency. See also Figure S7.

To expand this evaluation, we next treated several normal tissue-derived cells with trametinib or SHP2 and evaluated both the magnitude of pERK inhibition and cellular viability. We included retinal pigmented epithelial cells (RPE.1), as ocular toxicity is broadly reported with MEK inhibitors (Mendez-Martinez et al., 2019; Stjepanovic et al., 2016), as well as the cardiomyocytic AC16 cells, as cardiac toxicity is also reported with MEK inhibitor (Berger et al., 2020). While trametinib similarly downregulated pERK in NB and normal tissue-derived cells (Figure 3B), SHP099 only markedly downregulated pERK in NB cells, with either no (RPE.1 and MRC5 cells) or minimal (HCE-T and AC16 cells) effects in the normal tissue-derived cells (Figure 3B). Consistent with the maintenance of pERK signaling following SHP099, the normal tissue-derived cells were uniformly insensitive to SHP099 (Figures 3A-3C); in contrast, these cells had variable sensitivity to trametinib (Figures 3A-3C). In the RPE.1 cells, which besides modeling possible retina toxicity, which is commonly reported with MEK inhibitors (Mendez-Martinez et al., 2019), are also developmentally close to NB (Harenza et al., 2017). There is a particularly striking difference between sensitivity to SHP099 and trametinib (Figures 3A-3C). These data indicate at concentrations where SHP099 is effective in a large subset of NBs (5 μM) that normal tissue-derived cells maintain pERK and are insensitive to the drug, while MEK inhibition demonstrates broad toxicity accompanying pERK downregulation. We confirmed the differential sensitivity and effects on pERK signaling in the NB cells and the normal tissue-derived cells, with the more potent SHP2 inhibitor TNO-155 (Lochmann et al., 2018a; Song et al., 2018) (Figure S7). This presents a therapeutic window for SHP2 inhibitors not appreciated with MEK inhibitors that, when also considering unwanted MEK inhibitor-specific mTORC2 feedback, highlights a possible and particular utility of SHP2 inhibitors in a subset of NB (type 2).

### NF1 mutation and expression correlates with response of neuroblastoma to SHP2 inhibition

**Figure 4.**
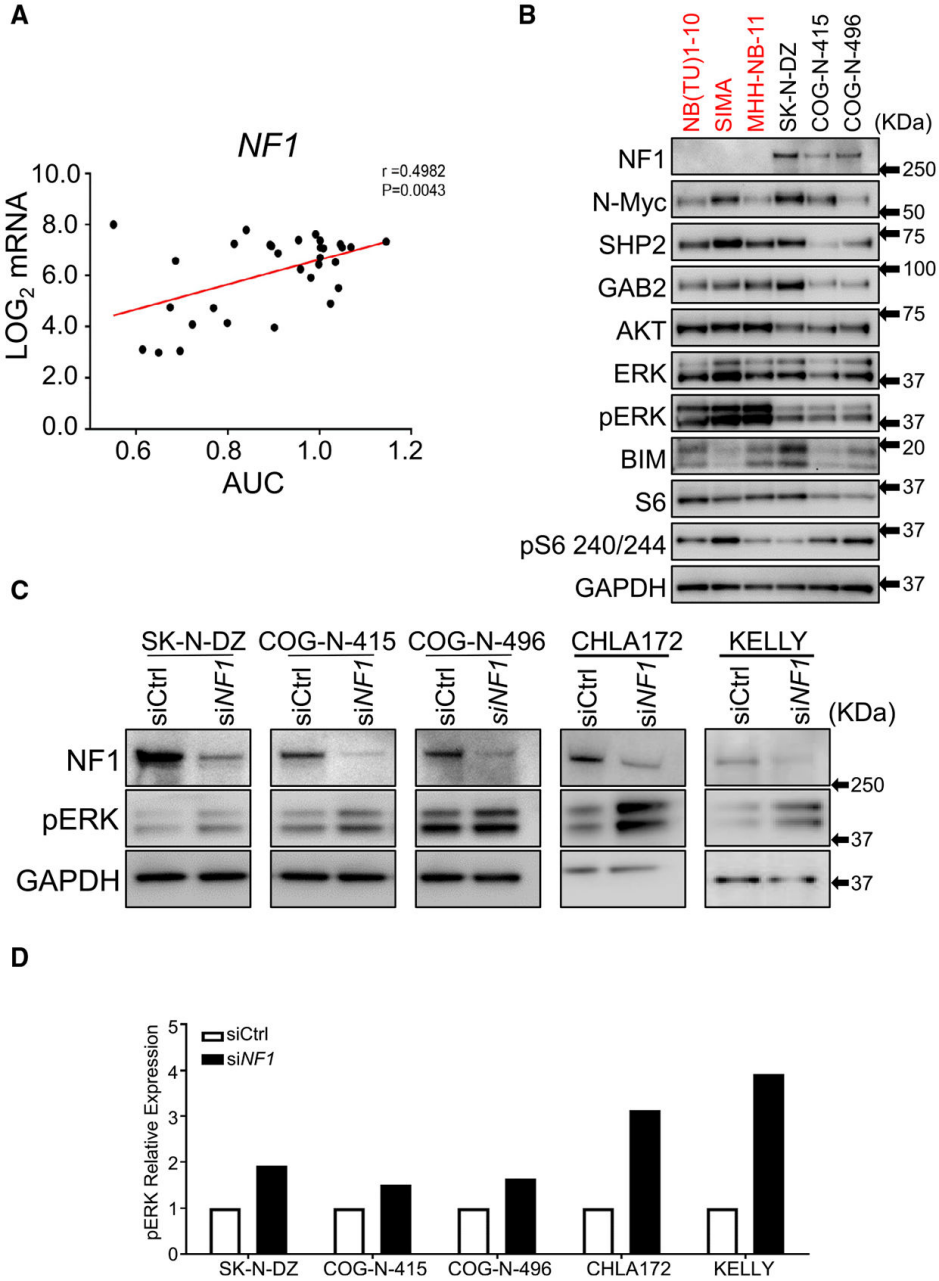
SHP099 sensitivity correlates with NF1 levels in neuroblastoma (A) Graph represents SHP099 sensitivity (area under the curve [AUC]) with respect to *NF1* transcript levels obtained from R2 genomic (Sanger) database (http://hgserver1.amc.nl) in 31 available NB cells lines from the HTS. Pearson correlation analysis value: *r* = 0.4982; p = 0.0043. (B) Immunoblot analysis of SHP099-sensitive (red) and SHP099-resistant (black) NB cell lines probed for NF1, SHP2, and other indicated antibodies. GAPDH was used as a loading control. (C) Immunoblot analysis of NF1 and pERK expression in NF1-silenced NB cell lines insensitive to SHP099. Scrambled oligonucleotide was used as an siRNA control (siCtrl), and GAPDH was used as a loading control. (D) Graph indicates relative levels of pERK in NF1-silenced NB cell lines quantified from (C). See also Figures S8-S11.

We sought to identify genomic correlates of SHP099 sensitivity in NB. MYCN was not associated with sensitivity: Many of the most sensitive NB lines are *MYCN* amplified, but the proportion in the most sensitive group (top 10%) is not different than in the non-sensitive group (Figures S8A and S8B). Gain of chromosome 17, the most common genetic event in NB, was also not statistically predictive of SHP099 sensitivity. Loss of chromosome arms 11q or 1p were also not predictive (Figure S8B). However, we found that SHP099 sensitivity was strongly correlated with mutational status (loss of function) of the RAS GTPase activation protein (GAP) neurofibromin 1 (NF1) (Figure S8C), with 6 of 10 NBs found in the top 10% sensitive cell lines (all tissue types) having NF1 loss of function (Figure 1A; p = 0.007, Fisher exact test). In addition, 6 of the total 9 NB cell lines harboring *NF1* mutations are in this group. *NF1* mutation and loss of NF1 expression is common in NB (Bollag et al., 1996). Consistent with the NF1 mutation data, low NF1 RNA levels were correlated with sensitivity to SHP099 (Figure 4A). Expression level of other known modulators of ERK signaling, including adaptor molecules and others that effect flux through the ERK pathway (Eleveld et al., 2018; Ran et al., 2016), such as GRB2 and GAB1, did not correlate with sensitivity (Figure S9). Consistent with NF1 known function (Bollag et al., 1996), *NF1* mutant models had high basal ERK phosphorylation (Figure 4B) and knockdown of NF1 with siRNA led to increased pERK levels in NF1-expressing NB (Figures 4C and 4D). However, small hairpin RNA (shRNA) deletion of NF-1 was not sufficient in the insensitive NB cell lines to confer sensitivity to SHP2 inhibition, suggesting pathway manipulation in NBs that are already developed is not sufficient to create a vulnerability to SHP2 inhibition (Figures S10A and S10B). Consistent with this notion, we did not find increased sensitivity to MEK inhibitor following NF1 deletion (Figure S10C). Lastly, the SHP2 *A72T* mutant found in NB (Pugh et al., 2013) did not confer enhanced sensitivity to either SHP2 inhibitor (Figure S11). These data altogether demonstrate that the loss of NF1 expression, either through mutation of NF1 itself or other mechanisms, is strongly predictive of sensitivity of NB cells to SHP099. NF1 mutation and expression may therefore constitute biomarkers for SHP099 in NB clinical trials.

### NF1 level is lower in high-risk neuroblastomas, is reduced as NB progresses, and is lower in chemorelapse patients

**Figure 5.**
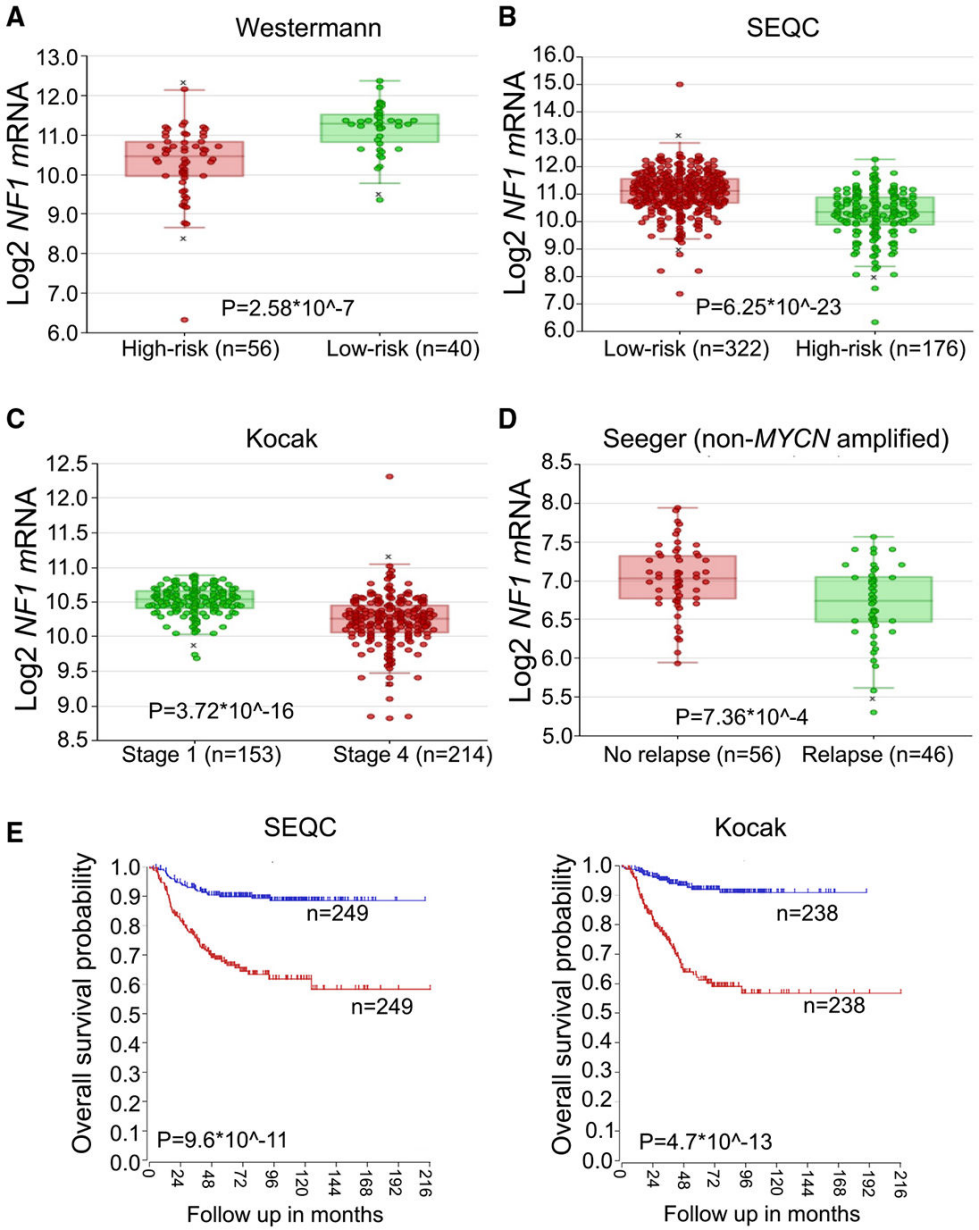
Low *NF1* levels correlate with high risk, tumor stage, recurrence, and patient outcome in neuroblastoma Transcript levels of *NF1* in high- and low-risk neuroblastoma obtained from the Westermann (A) and SEQC (B) datasets. (C) *NF1* transcript levels in respect to tumor stage obtained from the Kocak dataset. (D) *NF1* transcript levels in respect to relapsed and non-relapsed *MYCN*-non-amplified NB tumors obtained from the Seeger dataset. (E) Overall survival probability of patients with high (blue) and low (red) levels of *NF1* transcript obtained from SEQC and Kocak datasets.

As the need for new therapies is most acute in high-risk NB, we asked whether NF1 levels were low in high-risk NB. Indeed, in two datasets of NB tumors (Consortium, 2014; Henrich et al., 2016), we found low levels of NF1 transcript in high-risk NBs compared to low-risk NBs (Figures 5A and 5B). In addition, in the Kocak dataset (Kocak et al., 2013), where disease stage was available, NF1 levels decreased as stage increased (Figure 5C). In patients who relapsed with non-*MYCN*-amplified disease (Asgharzadeh et al., 2006), NF1 expression was lower than in patients in NBs who did not relapse (Figure 5D). Lastly, low NF1 levels portended poor survival (Figure 5E). These data together suggest that SHP099 may be particularly effective in high-risk NBs where new treatments are needed.

### *In vitro* and *in vivo* efficacy of SHP099 in NB

**Figure 6.**
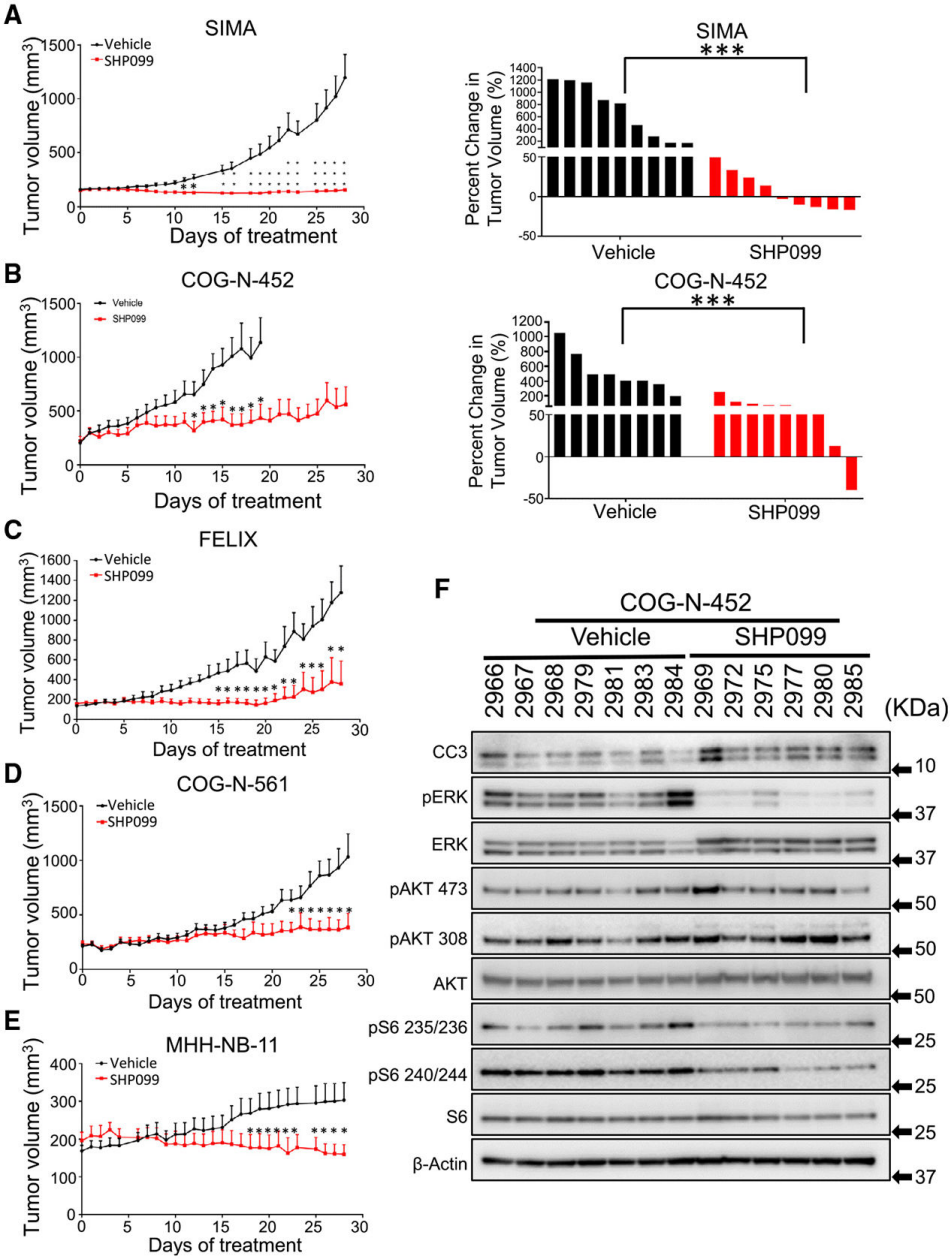
SHP099 treatment inhibits tumor growth in high-risk *in vivo* models of neuroblastomas (A–E) Antitumor activity of SHP099 was assessed in mice bearing high-risk NB tumors. Mice were dosed with SHP099 (75 mg/kg/day) or vehicle (control) for 28 days, and tumor volume was calculated using the formula v = (l × w × w) (π/6) Method details in the MYCN-amplified SIMA model (A), the *MYCN*-amplified COG-N-452 PDX model (B), the *ALK* mutant/*MYCN*-wild-type FELIX high-risk PDX model (C), the *MYCN*-amplified COG-N-561 PDX model (D), and the *MYCN*-amplified MHH-NB-11 model (E). Waterfall plot represents change in tumor volume percentage of each tumor to their initial tumor size (right panel) in the control and treated group. Error bars are ± SEMs. Student t test was used to calculate significance (*p < 0.05, **p < 0.01, ***p < 0.001). (F) Immunoblot of control (vehicle) and SHP099-treated COG-N-452 PDX mouse tumor tissues probed with the indicated antibodies. β-Actin was used as a loading control. See also Figures S12 and S13.

Based on the observed sensitivity of NB models and the ability of SHP099 to induce cell death in *RAS/RAF* WT NB cells, we next evaluated the efficacy of SHP099 against high-risk NB tumors *in vivo*. For these studies, we evaluated seven mouse models of high-risk NB: The *MYCN*-amplified (and therefore high-risk) SIMA and MHH-NB-11 models; the high-risk SK-N-FI model established from a metastatic site that is *MYCN*-WT but poorly differentiated; the *MYCN*-WT; the *ALK* mutant FELIX patient-derived xenograft (PDX) model (Sano et al., 2019a); CHLA20, a *MYCN*-WT PDX established at the progression of disease (Schoen et al., 2018); and two *MYCN*-amplified PDX models, COG-N452 and COG-N-561 (Lochmann et al., 2018b; Schoen et al., 2018). Details of these models are found in Table S3. These tumors were grown in immunocompromised mice, and upon reaching treatable sizes, were randomized, followed by treatment with 75 mg/kg/day SHP099 (Dardaei et al., 2018) or with vehicle. For the SIMA SHP099-treated group, complete growth inhibition was observed over 4 weeks (Figure 6A left), with 5 of 9 tumors shrinking (Figure 6A, right). For the COG-N-452 PDX tumors, while the effect was not as dramatic, the growth of the treated tumors was substantially slowed (Figure 6B). In the FELIX and COG-N-561 PDXs, tumor growth was controlled by SHP099 (Figures 6C and 6D), Lastly, in the MHH-NB-11 *MYCN*-amplified model, tumor growth was controlled (Figure 6E). For the SK-N-FI *MYCN*-WT xenograft model that grew slower *in vivo*, all SHP099-treated tumors regressed over 4 weeks’ treatment (Figure S13A). And in the slower growing CHLA20 PDX model, tumors were shrunk (Figure S13B). The individual growth of each tumor is demonstrated in Figure S12. We analyzed on-treatment tumors and measured pERK levels in the tumor lysates, finding SHP099 had on-target inhibition and evidence of cell death (cleaved caspase 3) (Figure 6F). In addition, similar to the *in vitro* results, there was no feedback activation of mTORC2, and downstream mTORC1 targets were downregulated (Figure 6F). Consistent with the HTS and *in vitro* data, trametinib did not have activity *in vivo* at a dose (1 mg/kg/day) that is effective in *BRAF* mutant models (Gilmartin et al., 2011) (Figure S13C). Lastly, the great majority of mice did not suffer significant weight loss on this dosing schedule (Figure S13D). Thus, *in vitro* activity of SHP099 translated to *in vivo* efficacy, consistent with past reports in other tumor types (Chen et al., 2016; Dardaei et al., 2018; Fedele et al., 2018; Nichols et al., 2018; Wong et al., 2018).

## Discussion

In this study, we integrate several HTS of MAPK inhibitors to demonstrate the unexpected hypersensitivity of WT *RAS/RAF* NB to SHP2 inhibition. SHP2 regulates the MEK/ERK pathway through multiple mechanisms. It is thought to affect RAS loading via its activity on RAS-GAP recruitment to RTK complexes (Montagner et al., 2005) via direct recruitment of Grb2 (Ran et al., 2016), dephosphorylation of RAS on a conserved Tyr32 residue increasing RAS-RAF association and subsequent MEK/ERK signaling (Kano et al., 2019), or via its effect on SRC (Zhang et al., 2004). SHP2 inhibition was found to be associated with the sensitivity of cancers driven by RTKs, through the disruption of RTK-driven, SHP2-mediated MEK/ERK activation (Chen et al., 2016). Efficacy has also been demonstrated in BCR-ABL-driven chronic myeloid leukemia CML (Gu et al., 2018), where SHP2 is a strong inducer of MEK/ERK signaling and through disruption of RTK-mediated feedback signaling in RAS-driven cancers (Wong et al., 2018).

Despite not being a classical RTK-driven or RAS-MAPK-driven cancer due to the absence of activating mutations or amplification in these genes, SHP2 inhibitor hypersensitivity in NB is consistent with some previous observations. First, in a tumor that presents with an average somatic mutation burden of ~3 (Hwang et al., 2018), SHP2 activating mutations are found in up to 3% of NB tumors, making it among the most common reoccurring mutations in these tumors (Bentires-Alj et al., 2004; Pugh et al., 2013). Second, Eleveld and colleagues reported that somatic mutation burden increases substantially in relapsed tumors, with diverse and reoccurring mutations of the MAPK pathway in nearly 80% of the relapse tumors (Eleveld et al., 2015); mutations that were not detected in the corresponding primary tumors included *NF1* loss, *HRAS Q61K* mutations, and *ALK* activating mutations, all of which would be expected to result in the activation of the MEK/ERK pathway. Modak and colleagues (Padovan-Merhar et al., 2016) similarly reported the enrichment of MAPK pathwayactivating mutations in chemorelapsed NB tumors, including in RAS and MAP3K1. Interestingly, Nichols and colleagues demonstrated that in several MAPK pathway mutant cancer-driven cancers, including non-V600E, class 3 *BRAF* mutant-driven cancers, *NF1* deletion-driven cancers, and *KRAS G12C* mutant cancers, SHP2 inhibition is effective at inhibiting downstream MEK/ERK signaling (Nichols et al., 2018).

**Figure 7.**
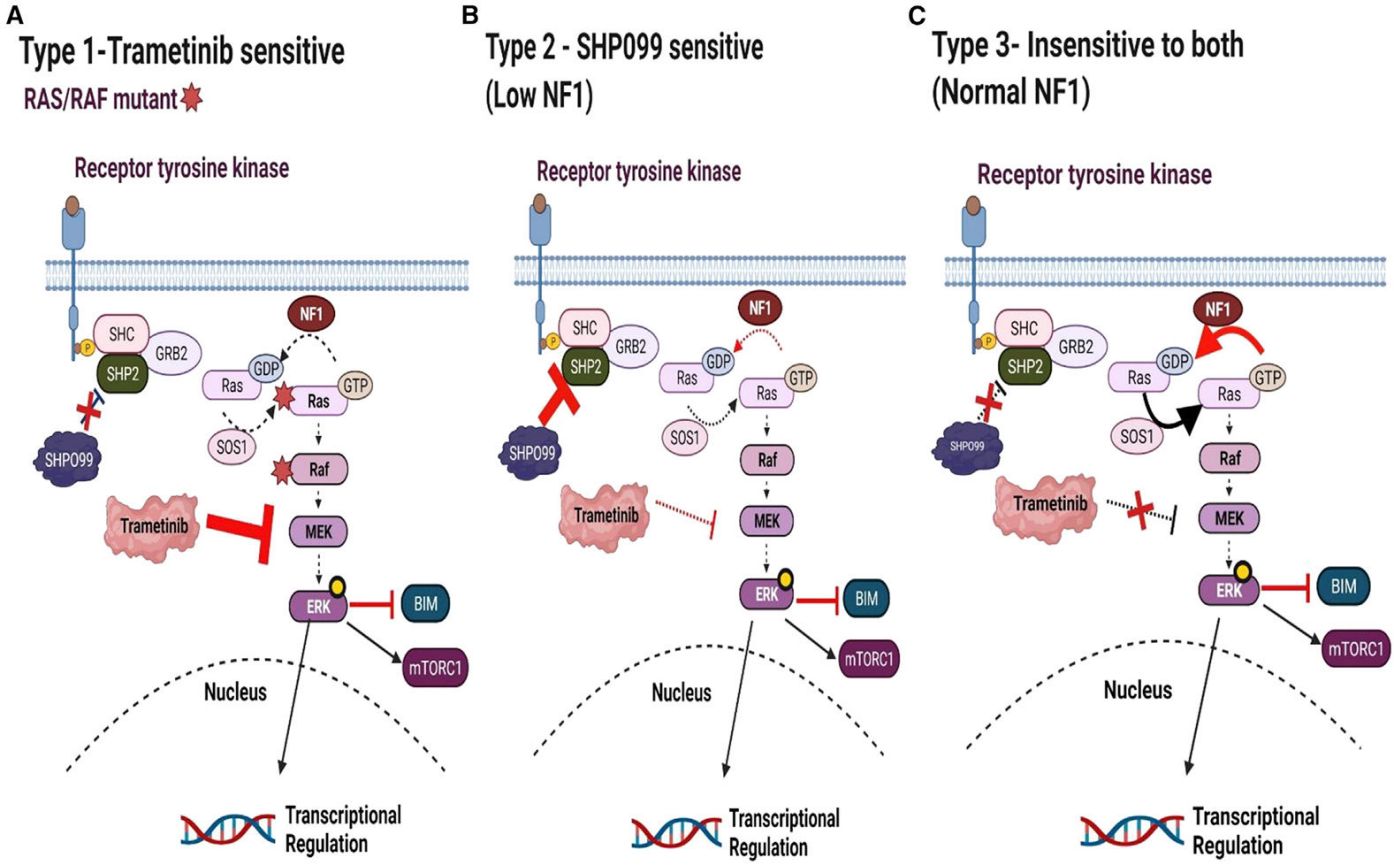
Model of sensitivity and insensitivity of NB to MAPK pathway inhibitors (A) We refer to type I NB as *RAS/RAF* MT NBs relatively sensitive to MEK inhibition and insensitive to SHP2 inhibition. (B) We refer to type II NB as *RAS/RAF* WT NBs with mutant/low NF1 that are sensitive to SHP2 inhibition and relatively insensitive to MEK inhibition. (C) We refer to type III NBs (RAS/RAF/NF1 WT). These are insensitive to SHP2 or MEK inhibitors.

MEK inhibitors have shown variable activity as single agents in preclinical testing in NB (Hart et al., 2017; Umapathy et al., 2017), and the presence of amplified *MYCN* correlates with resistance to the MEK inhibitor binimetinib (Hart et al., 2017). Analyses of our HTS show that WT *RAS/RAF* NB lines are not sensitive to MEK or ERK inhibition compared to effects seen in melanoma models (Figure 7; Figure S1). Many of these are sensitive to SHP2 inhibition, what we refer to in this study as type 2 NB cells, in reference to MAPK inhibitor response. In contrast, trametinib-sensitive NB cell lines (type 1) bear known *RAS/RAF* mutations and are insensitive to SHP099 (Figure 7; Figure S1; Table S2).

SHP2 inhibitors may be favored over MEK inhibitors in WT *RAS/RAF* NB for several reasons. First, while mutations that activate MEK/ERK signaling are prevalent in chemorelapse NB (Eleveld et al., 2015; Padovan-Merhar et al., 2016), most appear to be SHP2 dependent (e.g., ALK mutations, NF1 loss) and not *RAF/RAS* mutated. Second, SHP2 inhibition does not lead to the phosphorylation of mTORC2 substrates, as MEK inhibitors do in RAS/RAF WT NB (Figures 2B and S5) (Umapathy et al., 2017). Third, like NBs, we have found that normal derived tissues undergo the loss of pERK signaling following direct MEK inhibition (Figure 3); however, they retain pERK signaling following SHP2 inhibition. This difference in signaling changes between the two different inhibitors is consistent with the difference in sensitivity. That is, normal tissue-derived cells demonstrate variable sensitivity to MEK inhibitors but are not sensitive to SHP099. For instance, the h-TERT immortalized RPE.1 cells, neuronal in origin (Harenza et al., 2017), were quite sensitive to trametinib, yet completely resistant to SHP099 (Figure 3).

Lastly, we found that NF1 levels were inversely correlated with sensitivity to SHP099 (Figure 4), that NF1 loss increases baseline pERK flux (Figure 4), and that low NF1 levels associates with high-risk NB (Figure 5). However, manipulation of NF1 was not by itself sufficient to induce sensitivity to SHP2 inhibitors in high NF1 lines (Figures S10A and S10B). We interpret this to mean that NF1 pathway manipulation in NBs that are already developed is not sufficient to create a vulnerability to SHP2 inhibition. However, it is possible that co-occurring pathways must be present to confer SHP2 inhibitor sensitivity, when NF1 levels are low. This study did not look at different RTKs that may be acting together with NF1 to activate SHP2/RAS/MEK, EGFR, and FGFR1, FGFR2 and ALK have been found to associate with SHP2 pathway activation in NB (Zhang et al., 2017). Furtherworkwill be needed to determine what role RTKs, and which RTKs, if any, have in activating this pathway. NF1 germline deletions are syndromic for a cancer predisposition referred to as neurofibromatosis type 1 (Ling et al., 2005). The NF1 tumor-suppressor functions as a GAP for RAS promoting GTP hydrolysis and inhibiting MEK/ERK activation (Holzel et al., 2010). Consistent with our observation that low NF1-expressing NBs are sensitive to SHP099, Nichols and colleagues demonstrated that a different SHP2 allosteric inhibitor, RMC-4550, disrupts RAS loading and MEK/ERK activatior in several *NF1* mutant cancer cells (Nichols et al., 2018). Of note, a possible limitation of our studies is the duration of the *in vivo* experiments (~4 weeks). Indeed, it is not apparent what the duration of time that an anticancer drug should be tested for to potentially translate to a successful clinical candidate is, and concerns that longer periods of time may more precisely predict successful clinical candidates are reasonable.

Altogether, through a series of unbiased HTS and subsequent preclinical characterization, we demonstrate that NBs with low expression of NF1 are sensitive to SHP2 inhibition. Given the potential difference in therapeutic window with MEK inhibitor and the importance of NF1 levels for successful RA therapy (Holzel et al., 2010), we believe that SHP2 inhibition either by itself or incorporated into maintenance therapy for high-risk NB warrants further investigation.

### Limitations of the study

The limitations of this study include the relative short duration of the xenograft experiments, the inability of NF1 manipulation to directly impact SHP099 sensitivity, and the lack of comprehensive investigation of other mutations impacting SHP2-inhibitor sensitivity in NB.

## STAR★METHODS

### RESOURCE AVAILABILITY

#### Lead contact

Further information and requests for resources and reagents should be directed to and will be fulfilled by the lead contact, Anthony C. Faber (acfaber@vcu.edu).

#### Materials availability

All stable reagents generated in this study are available from the lead contact without restriction.

#### Data and code availability

The drug screen data have been attached as Table S1.

This paper does not report original code.

Any additional information required to reanalyze the data reported in this paper is available from the lead contact upon request.

### EXPERIMENTAL MODEL AND SUBJECT DETAILS

#### Mouse models

The xenograft models SIMA, MHH-NB-11, SK-N-FI, CHLA20 and the PDX models COG-N-452, COG-N-561 and Felix (PDXs kindly provided by C. Patrick Reynolds and the Childhood Cancer Repository at Texas Tech University Health Sciences Center, in part funded by Alex’s Lemonade Stand Foundation) were injected into the right flank of male NOD/SCID/IL2Rγ (NSG) mice (5–6 weeks) at the concentration of 7 × 10^6^ cells for SIMA, 4 × 10^6^ cells for MHH-NB-11 and 2 × 10^6^ cells for COG-N-452 and COG-N-561, or both flanks of male NOD-*Prkdc*^em26Cd52^*II2rg*^em26Cd22^/NjuCrl (NCG) mice (5–6 weeks) at the concentration of 2 × 10^6^ cells per injection for Felix, SK-N-FI and CHLA20 at the concentration of 2 × 10^6^ cells per tumor injection, using a 1:1 ratio of cells and Matrigel (Corning, cat #354248). When tumors reached about 150–200 mm^3^, the tumor-bearing mice were randomized to a vehicle group and a SHP099 treatment group (75 mg/kg). Mice in the treatment cohorts (n = 3–9) were treated with SHP099 or trametinib directly to the stomach by oral gavage. The solvent for SHP099 was 0.6% hydroxypropyl methylcellulose (HMC), 0.4% Tween 80, and 0.9% saline. The tumors were measured by digital calipers daily during the study in two dimensions (length and width), and the tumor volume was calculated with the formula v = (l × w × w) (π/6), where v is the tumor volume, l is the length (bigger measurement) and w is the width (smaller measurement). The drug schedule was once daily for four weeks (excluding Sundays). For pharmacodynamics studies, tumors were harvested 2h following the last SHP099 treatment and the tumors were snap frozen in liquid nitrogen. All mouse experiments were approved and performed in accordance with the Institutional Animal Care and Use Committee at Virginia Commonwealth University (VCU), under protocol AD10001048.

#### Cell lines

The neuroblastoma cell lines MHH-NB-11, KELLY, SK-N-DZ, SIMA, NB(TU)1-10 and SK-N-AS were from the Center for Molecular Center Therapeutics Laboratory at Massachusetts General Hospital. The RPE.1 cells and SK-N-FI were kindly provided by Dr. Yael Mosse (University of Pennsylvania, Children’s Hospital of Philadelphia). The HCE-T cells were purchased from RIKEN Biosource Center (RBC 2280) and MRC5 cells were purchased from ATCC (ATCC CCL-171). COG-N-415, COG-N-452, COG-N-496, COG-N-561, CHLA20 and CHLA172 PDX cell lines were kindly provided by C. Patrick Reynolds and the Childhood Cancer Repository at Texas Tech Health Sciences Center, in part funded by Alex’s Lemonade Stand Foundation. The MHH-NB-11 cell line was originally derived from a 4-year-old boy; the KELLY cell line was originally derived from a 1-year-old girl; the SK-N-DZ cell line was originally derived from a 2-year-old girl; the SIMA cell line was originally derived from a 1-year and 8 months-old boy; the NB(TU)1-10 cell line was originally derived from a 1-year and 8 months-old girl; the SK-N-AS cell line was originally derived from a 6-year-old girl; the RPE.1 cell line was originally derived from a 1-year-old girl; the SK-N-FI cell line was originally derived from a 11-year-old boy; the HCE-T cell line was originally derived from a 49-year-old women; the COG-N-415 cell line was originally derived from a 2-year-old girl; the COG-N-452 cell line was originally derived from a 5-year-old boy; the COG-N-496 cell line was originally derived from a 3-year and 2 months-old girl; the COG-N-561 cell line was originally derived from a 1-year and 8 months-old girl; the FELIX cell line was originally derived from a age unspecified boy; the CHLA20 cell line was originally derived from a 2-year-old girl; the CHLA-172 cell line was originally derived from a age unspecified boy. The MHH-NB-11, KELLY, SIMA, NB(TU)1-10, and RPE.1 cell lines were cultured in RPMI 1640 (the Lonza Group) supplemented with 10% fetal bovine serum (FBS, Seradigm) and 1 μg/mL penicillin and streptomycin. The HCE-T, SK-N-DZ, SK-N-FI and SK-N-AS cell lines were grown in Dulbecco’s modified Eagle medium (DMEM)/F12 (50:50) with 10% FBS and 1 mg/mL penicillin and streptomycin. The COG-N-415, COG-N-452, COG-N-496 and COG-N-561 cell lines were cultured in Iscove’s modified Dulbecco’s medium supplemented with 20% FBS, 1 μg/mL penicillin and streptomycin and 1× insulin-transferrin-selenium (ITS; Thermo Fisher Scientific, catalog #41400045). The MRC5 cell line was cultured in DMEM with 10% FBS, 1 μg/mL penicillin and streptomycin. The CHLA20 and CHLA172 cell lines were cultured in DMEM with 20% FBS, 1 × ITS and 1 μg/mL penicillin and streptomycin. Cell lines are routinely checked by DNA STR for verification and tested for mycoplasma infection by MycoAlert (Lonza). If the cell cutlures are found to be positive, they are treated with plasmocure (InvivoGen, catalog#ant-pc) per the manufacturer’s protocol until subsequent tests are negative.

### METHOD DETAILS

#### Drug screening

Certified cell lines were screened against SHP099 at the center for Molecular Therapeutics at the Massachusetts general hospital. Drug response metrics such as IC50 and Area under the dose–response curve (AUC) were determined for further analysis as previously described (Garnett et al., 2012) and the screen is described in detail elsewhere (Yang et al., 2013). The Genomics of Drug Sensitivity in Cancer (GDSC) screening platform at MGH utilizes fluorescence-based cell viability assays following 72 h of drug treatment. As described in (Cancer Cell Line Encyclopedia Consoritum and Genomics of Drug Sensitivity in Cancer Consoritum, 2015; Garnett et al., 2012), the HTS involves diverse compounds to evaluate anti-cancer effects across a broad number of cancer subtypes and the analyses of the data generated from the HTS includes dose-response curves are fitted to fluorescence signal intensities over nine drug concentrations (2-fold dilution series).

#### Expression analysis

The basal RNA expression data for the cell lines screened were obtained and analyzed through the R2: Genomics Analysis and Visualization Platform (http://hgserver1.amc.nl) using the GDSC-based Celline Cancer Drug (Sanger) dataset (Array Express Accession: E-MTAB-3610).

#### Antibodies and reagents

The primary antibodies for western blotting were n-Myc (cat #9405S), Gab2 (cat #3239S), Bim (cat #2819S), SHP2 (cat #3397T), phospho-SHP2 (Y542, cat #3751S), Akt (cat #4691S), phospho-Akt (T308, cat #4056S), phospho-Akt (S473, cat #4060S), S6 Ribosomal protein (cat #2217S), phospho-S6 Ribosomal Protein (S235/236, cat #4858S), phospho-S6 Ribosomal Protein (S240/244, cat #5364S), Erk (cat #4695S), phosphor-Erk (cat #4370S), NF1 (cat #14623S), cleaved PARP1 (cat #9541S), cleaved Caspase3 (cat #9664S), β-Actin (cat #4967S); all these antibodies are from Cell Signaling Technology (Beverly, MA). The GAPDH antibody (Wong et al., sc-32233) from Santa Cruz Biotechnology (Dallas, TX) was used as a loading control. The secondary antibodies used were anti-mouse IgG (GE Healthcare Life Sciences, cat #NXA931) and anti-rabbit IgG (GE Healthcare Life Sciences, cat #NA934). SHP099 (Wong et al., M6314) was from Abmole (Houston, TX), Trametinib (GSK 1120212, cat #CT-GSK212), RMC-4550 (Wong et al., CT-RMC4550) and TNO-155 (Wong et al., CT-TNO-155) were from ChemiTek (Indianapolis, IN). AZD-2014 was from Selleckchem (Wong et al., S2783). Crystal violet was from Thermo Scientific (cat #42583-0250).

#### Plasmid and cloning

The original NF1 plasmid R777-E139 Hs.NF1 was a gift from Dominic Esposito (Addgene plasmid # 70,423). The original PTPN11 plasmid pHAGE-PTPN11 was a gift from Gordon Mills & Kenneth Scott (Addgene plasmid # 116,782). NF1 and PTPN11 were subcloned into lentivirus expression vector by gateway clone method. Briefly, NF1 and PTPN11 ORF sequences were amplified from original plasmid with specific primers. The primers for NF1 were as following, NF1-F: 5′-CACCATGGCCGCCCACAGA-3′, NF1-R: 5′-TTACACGATTTTCTTGATGCTGTTCCG-3′, and for PTPN11, the primers for PTPN11 were as following, PTPN11-F: 5′-CACCATGACATCGCGGAGATGG-3′, PTPN11-R: 5′-CTACCTGCAGTGCACCACGACCGGCCC-3′. After ethanol precipitation, Gateway compatible amplified ORFs were recombined into pENTR^™^/SD/D-TOPO vector (Invitrogen) using the pENTR^™^/SD/D-TOPO cloning kit (Invitrogen). The mix was then transformed with OneShot TOP10 competent cells, and the clones were analyzed and sequenced with M13 forward and reverse primer to confirm the correct insertions. Once the entry clones were confirmed, the LR recombination reaction was carried out to transfer the target genes from the entry constructs into the pLenti6/V5-DEST vector using the Gateway^™^ LR Clonase^™^ Enzyme Mix (Invitrogen) to generate an expression clone. After reaction, the mix was transformed with OneShot Stbl3 chemically competent cells. The single clones were analyzed and sequenced with CMVforward primer and V5 reverse primer. For SHP2 A72T mutation, the reaction was performed with the mutation primers when the PTPN11 gene was cloned into the pENTR^™^/SD/D-TOPO vector. The mutation primers were listed as following, PTPN11mut F: 5′-CTCAGCCAAAGTGGTA AATTTCTCCCCTCCATACA-3′, PTPN11mut R: 5′-TGTATGGAGGGGAGAAATTTACCACTTTGGCTGAG-3′. When the mutation was confirmed in the pENTR vector, the LR reaction was carried out further to construct the mutation expression vector in pLenti6/V5-DEST. For GAB2 expression, the plasmid pLX317-EGFP and pLX317-GAB2 were gift from Dr. William Hahn (Dana-Farber Cancer Institute, Boston).

#### Cell viability assays

For CellTiter-Glo (CTG) (Promega) experiments, cells were seeded in quadruplicate in 96-well black plates at a concentration of 2 × 10^3^ cells per well in 180 μL of growth medium. 24 h after seeding, cells were treated with increasing concentrations of SHP099 from 0 to 10 μM for 7 days and maintained at 37°C and 5% CO_2_. Cell viability was read on a Centro LB960 microplate luminometer (Berthold Technologies) according to the manufacturer’s instructions, with the exception that half of the recommended CTG reagent was used. For crystal violet assays, 50,000 cells were seeded in 6-well plate and the next day cells were treated with different drugs as indicated in the figure legends. Every 3 days, the growth medium was replenished to ensure sufficient nutrients. Once the cells in the no-treatment control well approached full confluence as determined by microscope, the cells were stained with 0.1% crystal violet (Sigma-Aldrich). Some of the STR testing for these cell lines can be found in Data S1.

#### siRNA experiments

For the siRNA experiments, *NF1* and scrambled control siRNA (Dharmacon smart pool) were used at the concentration of 25 nM and transfected with lipofectamine RNAiMAX reagent (ThermoFisher). Briefly, cells were seeded in 60mm dish with antibiotic-free media to achieve next-day confluency of roughly 60%. When doing the transfection, 18 μL RNAiMAX was added to 300 μL of OPTI-MEM in a 1.5 mL Eppendorf tube. At the same time, 25nM siRNA was added to 300 μL of OPTI-MEM in a separate 1.5 mL Eppendorf tube. After 5 min, the tubes were combined and mixed gently. Following 20 more minutes, the RNAiMAX-siRNA mix was drop-wise added to cells and gently shaken for couples of times. Twenty-four hours later, the cells were either reseeded in 96-well plates and treated with the appropriate drug the following day or reseeded in 6-well plates and lysed the next day for Western blot analysis to determine the efficiency of the knockdown. The siRNA sequences used in the study were as follows: siRNA NF1 (ON TARGET plus SMART pool cat. no. L-003916-00-0005 5 nmol; Dharmacon) and siRNA scrambled (ON-TARGET plus Non-targeting Control Pool cat. no. D-001810-10-20; Dharmacon).

#### shRNA experiments

The plasmids for sh*NF1* (clone ID: sh*NF1*-1 NM_000,267.1-8468s1c1 and sh*NF1*-2 NM_000,267.1-8627s1c1) were obtained from the MISSION shRNA repository (Sigma-Aldrich). A nonspecific shRNA (MISSION pLKO.1-shRNA control, cat. no. SHC016-1EA) served as a negative control. The pLKO.1 plasmid contains a puromycin resistance gene, which allowed for cell selection. Cells were transduced with plasmid-containing viral particles and viral particles were generated in 293T cells and collected over 48 h.

#### Lentivirus production and stable cell line

For the knockout and knockdown lentivirus production, the expression plasmid was transfected into HEK 293T cells together with psPAX.2 and pMD2.G by lipofectamine 2000. For the overexpression lentivirus production, the expression plasmid was transfected into HEK293T cells together with pCMV-VSV-G and pCMV delta R8.2 using lipofectamine 2000. 48 h later, the virus was harvested for the first time and filtered with a 0.45 mm size filter, 10mL of media was added back to the transfected cells and then the virus was harvested the second time 72 h later. The target cells were transduced with the harvested lentivirus together with 8 μg/mL polybrene. 24 h later the cells are placed under selection with the appropriate antibiotic. Target gene knockdown/expression was confirmed by western blotting.

#### Western blotting

Cell lines, tumors from cell-line xenografts and tumors from patient-derived xenografts were prepared and lysed in NP40-containing cell lysis buffer along with 20 mM Tris, 150 mM NaCI, 1% NP-40, 1 mM EDTA, 1 mM EGTA, 10% Glycerol, and protease and phosphatase inhibitors. The samples were incubated on ice for 30 min, then centrifuged at maximum speed on a tabletop micro-centrifuge for 10 min at 4°C. For the tumor lysates, additional preparation included manual shearing with scissors into pieces and then homogenization with Tissuemiser (Fisher Scientific) in the same lysis buffer on ice, followed by a 30 min incubation period on ice and centrifugation. Total protein amount was quantified by BCA assay and equal amounts of the detergent-soluble lysates were separated with the NuPAGE Novex Midi Gel system on 4%–12% Bis-Tris Gels (Invitrogen), transferred to PVDF membranes (PerkinElmer) in transfer buffer (Biorad) with 20% methanol. Following transferring, the membrane was blocked in PBS-T with 5% non-fat milk for 1h and then incubated with the indicated antibodies overnight. After secondary antibody (GE Healthcare) incubation, the antibodies on the membranes were detected with the Syngene G: Box camera (Synoptics). Representative blots are shown in the figures. Band quantification was done using the Gene Tools software program (Syngene). The quantification data is presented as the ratio of the indicated readout/GAPDH for each data point.

### QUANTIFICATION AND STATISTICAL ANALYSIS

Non-parametric Mann-Whitney U-tests were performed for Figures S4, S8B, and S13D. Unpaired student’s test (two-tailed) was performed for Figures 3A, 5, 6, S2A, S6A, S6B, S10B, S10C, S11B, S13A, and S13B. Figures 4A and S9 were analyzed with Pearson Correlation analysis. All the analyses were performed using GraphPad Prism. Differences considered to be significant if p < 0.05.

## Supplementary Material

1

2

3

4

5

## Figures and Tables

**Table:** KEY RESOURCES TABLE

REAGENT or RESOURCE	SOURCE	IDENTIFIER
Antibodies		
p-ERK	Cell Signaling Technology	Cat# 4370; RRID:AB_2315112
ERK	Cell Signaling Technology	Cat#4695; RRID:AB_390779
p-AKT S473	Cell Signaling Technology	Cat#4060; RRID:AB_2315049
p-AKT T308	Cell Signaling Technology	Cat#4056S; RRID:AB_331163
AKT	Cell Signaling Technology	Cat#4691; RRID:AB_915783
cleaved PARP	Cell Signaling Technology	Cat#5625; RRID:AB_10699459
Gab2	Cell Signaling Technology	Cat# 3239; RRID:AB_10698601
BIM	Cell Signaling Technology	Cat# 2819; RRID:AB_10692515
SHP2	Cell Signaling Technology	Cat# 3397; RRID:AB_2174959
Phospho-SHP2	Cell Signaling Technology	Cat# 3751; RRID:AB_330825
S6 Ribosomal protein	Cell Signaling Technology	Cat# 2217; RRID:AB_331355
Phospho-S6 Ribosomal Protein (Ser235/236)	Cell Signaling Technology	Cat# 4858; RRID:AB_916156
Phospho-S6 Ribosomal Protein (Ser240/244)	Cell Signaling Technology	Cat# 5364; RRID:AB_10694233
Neurofibromin 1	Cell Signaling Technology	Cat# 14623; RRID:AB_2798543
Cleaved PARP (Asp214)	Cell Signaling Technology	Cat# 9541; RRID:AB_331426
Cleaved Caspase-3 (Asp175)	Cell Signaling Technology	Cat# 9664; RRID:AB_2070042
β-Actin	Cell Signaling Technology	Cat# 4967; RRID:AB_330288
Anti-GAPDH Antibody (6C5)	Santa Cruz	Cat# sc-32233; RRID:AB_627679
anti-mouse IgG	Millipore Sigma	NXA931; RRID:AB_772209
anti-rabbit IgG	Millipore Sigma	Cat# NA934; RRID:AB_772206
Bacterial and virus strains		
NF1 CRISPR-lentivirus	Virus prepared in lab	NF1 CRISPR Guide RNA
NF1 shRNA-lentivirus	Virus prepared in lab	sh*NF1*-1 NM_000267. 1-8468s1c1 and sh*NF1*-2 NM_000267. 1-8627s1c1
OneShot TOP10 competent cell	Invitrogen	C404010
OneShot Stbl3 competent cell	Invitrogen	C737303
Chemicals, peptides, and recombinant proteins		
SHP-099	Abmole	Cat#M6314
Trametinib	ChemieTek	Cat#CT-GSK212
VX-11E	APEXBIO	Cat#A3931
RMC-4550	Chemietek	Cat#CT-RMC4550
TNO-155	Chemietek	Cat#CT-TNO155
Crystal violet	Thermo Scientific	Cat#42583-0250
AZD2014	Selleckchem	Cat#S2783
Deposited data		
Genomics Analysis and Visualization Platform	http://hgserver1.amc.nl	E-MTAB-3610
Experimental models: Cell lines		
SIMA	Center for Molecular Center Therapeutics Laboratory at Massachusetts General Hospital	Cat# ACC-164; RRID:CVCL_1695
KELLY	Center for Molecular Center Therapeutics Laboratory at Massachusetts General Hospital	Cat# ACC-355; RRID:CVCL_2092
SK-N-DZ	Center for Molecular Center Therapeutics Laboratory at Massachusetts General Hospital	ATCC Cat# CRL-2149; RRID:CVCL_1701
SK-N-AS	Center for Molecular Center Therapeutics Laboratory at Massachusetts General Hospital	ATCC Cat# CRL-2137; RRID:CVCL_1700
SK-N-FI	University of Pennsylvania, Children’s Hospital of Philadelphia	ATCC Cat# CRL-2142; RRID:CVCL_1702
CHLA20	C. Patrick Reynolds and the Childhood Cancer Repository at Texas Tech	RRID:CVCL_6602
CHLA172	C. Patrick Reynolds and the Childhood Cancer Repository at Texas Tech	RRID:CVCL_A056
COG-N-415	C. Patrick Reynolds and the Childhood Cancer Repository at Texas Tech	RRID:CVCL_AQ23
COG-N-496	C. Patrick Reynolds and the Childhood Cancer Repository at Texas Tech	RRID:CVCL_LF61
COG-N-452	C. Patrick Reynolds and the Childhood Cancer Repository at Texas Tech	RRID:CVCL_VS96
NB(TU)1-10	Center for Molecular Center Therapeutics Laboratory at Massachusetts General Hospital	JCRB Cat# JCRB0154; RRID:CVCL_3041
MHH-NB-11	Center for Molecular Center Therapeutics Laboratory at Massachusetts General Hospital	DSMZ Cat# ACC-157; RRID:CVCL_1412
RPE1	University of Pennsylvania, Children’s Hospital of Philadelphia	ATCC Cat# CRL-4000; RRID:CVCL_4388
HCE-T	RCB	RCB Cat# RCB2280; RRID:CVCL_1272
MRC5	RCB	Cat# CCL-171; RRID:CVCL_0440
AC-16	Dr. Fadi Salloum, Virginia Commonwelath University	N/A
Experimental models: Organisms/strains		
Mouse: NOD/SCID/IL2Rγ (NSG)	The Jackson Laboratory	005557
Mouse: NOD-*Prkdc*^em26Cd52^ *II2rg*^em26Cd22^/NjuCrl (NCG)	Charles River	N/A
Oligonucleotides		
PTPN11-F: 5′-CACCATGACAT CGCGGAGATGG-3′	Thermofisher	N/A
PTPN11-R: 5′-CTACCTGCAGTGC ACCACGACCGGCCC-3′	Thermofisher	N/A
MISSON siRNA NF1	Dharmacon	Cat#L-003916-00-0005
siRNA scrambled	Dharmacon	Cat#D-001810-10-20
PTPN11mut F: 5′-CTCAGCCAAAG TGGTAAATTTCTCCCCTCCATACA-3′	Thermofisher	N/A
MISSION shRNA NF1	Sigma-Aldrich	sh*NF1*-1 NM_000267. 1-8468s1c1
MISSION shRNA NF1	Sigma-Aldrich	sh*NF1*-2 NM_000267. 1-8627s1c1
MISSION nonspecific pLKO. 1-shRNA control,	Sigma-Aldrich	cat#SHC016-1EA
PTPN11mut R: 5′-TGTATGGAGGG GAGAAATTTACCACTTTGGCTGAG-3′	Thermofisher	N/A
NF1-F: 5′-CACCATGGCCGCCCACAGA-3′	Thermofisher	N/A
NF1-R: 5′-TTACACGATTTTCTTGAT GCTGTTCCG-3′	Thermofisher	N/A
Recombinant DNA		
pLX317-EGFP	Dr. William Hahn, Dana-Farber Cancer Institute	N/A
pLX317-GAB2	Dr. William Hahn, Dana-Farber Cancer Institute	N/A
R777-E139 Hs.NF1	Addgene	Addgene Cat# 70423
pHAGE-PTPN11	Addgene	Addgene Cat## 116782
Software and algorithms		
Prism software	GraphPad	https://www.graphpad.com/scientific-software/prism/

## Highlights

- We identify neuroblastoma models as sensitive to SHP2 inhibition
- SHP099 sensitivity is correlated with low expression of neurofibromin (NF1)
- SHP2 inhibitors inhibit pERK in neuroblastoma cells better than in normal cells
- NF1 mutation/expression may predict SHP2 inhibitor response in neuroblastoma

## References

[R1] Ahmed TA, Adamopoulos C, Karoulia Z, Wu X, Sachidanandam R, Aaronson SA, and Poulikakos PI (2019). SHP2 drives adaptive resistance to ERK signaling inhibition in molecularly defined subsets of ERK-dependent tumors. Cell Rep. 26, 65–78.e5. 10.1016/j.celrep.2018.12.013. 30605687 PMC6396678

[R2] Asgharzadeh S, Pique-Regi R, Sposto R, Wang H, Yang Y, Shimada H, Matthay K, Buckley J, Ortega A, and Seeger RC (2006). Prognostic significance of gene expression profiles of metastatic neuroblastomas lacking MYCN gene amplification. J. Natl. Cancer Inst 98, 1193–1203. 10.1093/jnci/djj330. 16954472

[R3] Bentires-Alj M, Paez JG, David FS, Keilhack H, Halmos B, Naoki K, Maris JM, Richardson A, Bardelli A, Sugarbaker DJ, (2004). Activating mutations of the noonan syndrome-associated ***SHP2/PTPN11*** gene in human solid tumors and adult acute myelogenous leukemia. Cancer Res. 64, 8816–8820. 10.1158/0008-5472.can-04-1923. 15604238

[R4] Berger M, Amini-Adle M, Maucort-Boulch D, Robinson P, Thomas L, Dalle S, and Courand PY (2020). Left ventricular ejection fraction decrease related to BRAF and/or MEK inhibitors in metastatic melanoma patients: a retrospective analysis. Cancer Med. 9, 2611–2620. 10.1002/cam4.2922. 32056395 PMC7163110

[R5] Bollag G, Clapp DW, Shih S, Adler F, Zhang YY, Thompson P, Lange BJ, Freedman MH, McCormick F, Jacks T, and Shannon K (1996). Loss of NF1 results in activation of the Ras signaling pathway and leads to aberrant growth in haematopoietic cells. Nat. Genet 12, 144–148. 10.1038/ng0296-144. 8563751

[R6] Brachmann SM, Ueki K, Engelman JA, Kahn RC, and Cantley LC (2005). Phosphoinositide 3-kinase catalytic subunit deletion and regulatory subunit deletion have opposite effects on insulin sensitivity in mice. Mol. Cell Biol 25, 1596–1607. 10.1128/mcb.25.5.1596-1607.2005. 15713620 PMC549361

[R7] Cancer Cell Line Encyclopedia Consortium; Genomics of Drug Sensitivity in Cancer Consortium (2015). Pharmacogenomic agreement between two cancer cell line data sets. Nature 528, 84–87. 10.1038/nature15736. 26570998 PMC6343827

[R8] Chen YNP, LaMarche MJ, Chan HM, Fekkes P, Garcia-Fortanet J, Acker MG, Antonakos B, Chen CHT, Chen Z, Cooke VG, (2016). Allosteric inhibition of SHP2 phosphatase inhibits cancers driven by receptor tyrosine kinases. Nature 535, 148–152. 10.1038/nature18621. 27362227

[R9] Consortium SM-I (2014). A comprehensive assessment of RNA-seq accuracy, reproducibility and information content by the Sequencing Quality Control Consortium. Nat. Biotechnol 32, 903–914. 10.1038/nbt.2957. 25150838 PMC4321899

[R10] Cooper MJ, Hutchins GM, Cohen PS, Helman LJ, Mennie RJ, and Israel MA (1990). Human neuroblastoma tumor cell lines correspond to the arrested differentiation of chromaffin adrenal medullary neuroblasts. Cell Growth Differ. 1, 149–159. 1982060

[R11] Corcoran RB, Rothenberg SM, Hata AN, Faber AC, Piris A, Nazarian RM, Brown RD, Godfrey JT, Winokur D, Walsh J, (2013). TORC1 suppression predicts responsiveness to RAF and MEK inhibition in BRAF-mutant melanoma. Sci. Transl. Med 5, 196ra98. 10.1126/scitranslmed.3005753. PMC3867020 23903755

[R12] Dardaei L, Wang HQ, Singh M, Fordjour P, Shaw KX, Yoda S, Kerr G, Yu K, Liang J, Cao Y, (2018). SHP2 inhibition restores sensitivity in ALK-rearranged non-small-cell lung cancer resistant to ALK inhibitors. Nat. Med 24, 512–517. 10.1038/nm.4497. 29505033 PMC6343825

[R13] Eleveld TF, Oldridge DA, Bernard V, Koster J, Daage LC, Diskin SJ, Schild L, Bentahar NB, Bellini A, Chicard M, (2015). Relapsed neuroblastomas show frequent RAS-MAPK pathway mutations. Nat. Genet 47, 864–871. 10.1038/ng.3333. 26121087 PMC4775079

[R14] Eleveld TF, Schild L, Koster J, Zwijnenburg DA, Alles LK, Ebus ME, Volckmann R, Tijtgat GA, van Sluis P, Versteeg R, and Molenaar JJ (2018). RAS-MAPK pathway-driven tumor progression is associated with loss of CIC and other genomic aberrations in neuroblastoma. Cancer Res. 78, 6297–6307. 10.1158/0008-5472.can-18-1045. 30115695

[R15] Faber AC, Ebi H, Costa C, and Engelman JA (2012). Apoptosis in targeted therapy responses: the role of BIM. Adv. Pharmacol 65, 519–542. 10.1016/B978-0-12-397927-8.00016-6. 22959036

[R16] Fedele C, Ran H, Diskin B, Wei W, Jen J, Geer MJ, Araki K, Ozerdem U, Simeone DM, Miller G, (2018). SHP2 inhibition prevents adaptive resistance to MEK inhibitors in multiple cancer models. Cancer Discov. 8, 1237–1249. 10.1158/2159-8290.CD-18-0444. 30045908 PMC6170706

[R17] Feoktistova M, Geserick P, and Leverkus M (2016). Crystal violet assay for determining viability of cultured cells. Cold Spring Harb. Protoc. 2016, pdb. prot087379. 10.1101/pdb.prot087379. 27037069

[R18] Gao RN, Levy IG, Woods WG, Coombs BA, Gaudette LA, Hill GB, and Hill G (1997). Incidence and mortality of neuroblastoma in Canada compared with other childhood cancers. Cancer Causes Control 8, 745–754. 10.1023/a:1018483405637. 9328197

[R19] Garnett MJ, Edelman EJ, Heidorn SJ, Greenman CD, Dastur A, Lau KW, Greninger P, Thompson IR, Luo X, Soares J, (2012). Systematic identification of genomic markers of drug sensitivity in cancer cells. Nature 483, 570–575. 10.1038/nature11005. 22460902 PMC3349233

[R20] Gilmartin AG, Bleam MR, Groy A, Moss KG, Minthorn EA, Kulkarni SG, Rominger CM, Erskine S, Fisher KE, Yang J, (2011). GSK1120212 (JTP-74057) is an inhibitor of MEK activity and activation with favorable pharmacokinetic properties for sustained in vivo pathway inhibition. Clin. Cancer Res 17, 989–1000. 10.1158/1078-0432.ccr-10-2200. 21245089

[R21] Gu S, Sayad A, Chan G, Yang W, Lu Z, Virtanen C, Van Etten RA, Neel BG, and SAyAd A (2018). SHP2 is required for BCR-ABL1-induced hematologic neoplasia. Leukemia 32, 203–213. 10.1038/leu.2017.250. 28804122 PMC6005183

[R22] Harenza JL, Diamond MA, Adams RN, Song MM, Davidson HL, Hart LS, Dent MH, Fortina P, Reynolds CP, and Maris JM (2017). Transcriptomic profiling of 39 commonly-used neuroblastoma cell lines. Sci. Data 4, 170033. 10.1038/sdata.2017.33. 28350380 PMC5369315

[R23] Hart LS, Rader J, Raman P, Batra V, Russell MR, Tsang M, Gagliardi M, Chen L, Martinez D, Li Y, (2017). Preclinical therapeutic synergy of MEK1/2 and CDK4/6 inhibition in neuroblastoma. Clin. Cancer Res 23, 1785–1796. 10.1158/1078-0432.ccr-16-1131. 27729458

[R24] He S, Mansour MR, Zimmerman MW, Ki DH, Layden HM, Akahane K, Gjini E, de Groh ED, Perez-Atayde AR, Zhu S, (2016). Synergy between loss of NF1 and overexpression of MYCN in neuroblastoma is mediated by the GAP-related domain. Elife 5, e14713. 10.7554/elife.14713. 27130733 PMC4900799

[R25] Healy JR, Hart LS, Shazad AL, Gagliardi ME, Tsang M, Elias J, Ruden J, Farrel A, Rokita JL, Li Y, (2020). Limited antitumor activity of combined BET and MEK inhibition in neuroblastoma. Pediatr. Blood Cancer 67, e28267. 10.1002/pbc.28267. 32307821 PMC7188563

[R26] Henrich KO, Bender S, Saadati M, Dreidax D, Gartlgruber M, Shao C, Herrmann C, Wiesenfarth M, Parzonka M, Wehrmann L, (2016). Integrative genome-scale Analysis identifies epigenetic mechanisms of transcriptional deregulation in unfavorable neuroblastomas. Cancer Res. 76, 5523–5537. 10.1158/0008-5472.can-15-2507. 27635046

[R27] Hölzel M, Huang S, Koster J, Ora I, Lakeman A, Caron H, Nijkamp W, Xie J, Callens T, Asgharzadeh S, (2010). NF1 is a tumor suppressor in neuroblastoma that determines retinoic acid response and disease outcome. Cell 142, 218–229. 10.1016/j.cell.2010.06.004. 20655465 PMC2913027

[R28] Hwang WL, Wolfson RL, Niemierko A, Marcus KJ, DuBois SG, and Haas-Kogan D (2018). Clinical impact of tumor mutational burden in neuroblastoma. J. Natl. Cancer Inst 111, 695–699. 10.1093/jnci/djy157 PMC6624164 30307503

[R29] Infante JR, Fecher LA, Falchook GS, Nallapareddy S, Gordon MS, Becerra C, DeMarini DJ, Cox DS, Xu Y, Morris SR, (2012). Safety, pharmacokinetic, pharmacodynamic, and efficacy data for the oral MEK inhibitor trametinib: a phase 1 dose-escalation trial. Lancet Oncol. 13, 773–781. 10.1016/s1470-2045(12)70270-x. 22805291

[R30] Kano Y, Gebregiworgis T, Marshall CB, Radulovich N, Poon BPK, St-Germain J, Cook JD, Valencia-Sama I, Grant BMM, Herrera SG, (2019). Tyrosyl phosphorylation of KRAS stalls GTPase cycle via alteration of switch I and II conformation. Nat. Commun 10, 224. 10.1038/s41467-018-08115-8. 30644389 PMC6333830

[R31] Kiessling MK, Curioni-Fontecedro A, Samaras P, Lang S, Scharl M, Aguzzi A, Oldrige DA, Maris JM, and Rogler G (2016). Targeting the mTOR complex by everolimus in NRAS mutant neuroblastoma. PLoS One 11, e0147682. 10.1371/journal.pone.0147682. 26821351 PMC4731059

[R32] Kitai H, Ebi H, Tomida S, Floros KV, Kotani H, Adachi Y, Oizumi S, Nishimura M, Faber AC, and Yano S (2016). Epithelial-to-mesenchymal transition defines feedback activation of receptor tyrosine kinase signaling induced by MEK inhibition in KRAS mutant lung cancer. Cancer Discov. 6, 754–769. 27154822 10.1158/2159-8290.CD-15-1377 PMC4957999

[R33] Kocak H, Ackermann S, Hero B, Kahlert Y, Oberthuer A, Juraeva D, Roels F, Theissen J, Westermann F, Deubzer H, (2013). Hox-C9 activates the intrinsic pathway of apoptosis and is associated with spontaneous regression in neuroblastoma. Cell Death Dis. 4, e586. 10.1038/cddis.2013.84. 23579273 PMC3668636

[R34] Lhuissier E, Bazille C, Aury-Landas J, Girard N, Pontin J, Boittin M, Boumediene K, and Baugé C (2017). Identification of an easy to use 3D culture model to investigate invasion and anticancer drug response in chondrosarcomas. BMC Cancer 17, 490. 10.1186/s12885-017-3478-z. 28720081 PMC5516396

[R35] Ling BC, Wu J, Miller SJ, Monk KR, Shamekh R, Rizvi TA, Decourten-Myers G, Vogel KS, DeClue JE, and Ratner N (2005). Role for the epidermal growth factor receptor in neurofibromatosis-related peripheral nerve tumorigenesis. Cancer Cell 7, 65–75. 10.1016/j.ccr.2004.10.016. 15652750 PMC2854500

[R36] Lito P, Pratilas CA, Joseph EW, Tadi M, Halilovic E, Zubrowski M, Huang A, Wong WL, Callahan MK, Merghoub T, (2012). Relief of profound feedback inhibition of mitogenic signaling by RAF inhibitors attenuates their activity in BRAFV600E melanomas. Cancer Cell 22, 668–682. 10.1016/j.ccr.2012.10.009. 23153539 PMC3713778

[R37] Lochmann TL, Bouck YM, and Faber AC (2018a). BCL-2 inhibition is a promising therapeutic strategy for small cell lung cancer. Oncoscience 5, 218–219. 10.18632/oncoscience.455. 30234143 PMC6142900

[R38] Lochmann TL, Powell KM, Ham J, Floros KV, Heisey DAR, Kurupi RIJ, Calbert ML, Ghotra MS, Greninger P, Dozmorov M, (2018b). Targeted inhibition of histone H3K27 demethylation is effective in high-risk neuroblastoma. Sci. Transl. Med 10, eaao4680. 10.1126/scitranslmed.aao4680. 29769286 PMC6200133

[R39] Lopez-Delisle L, Pierre-Eugène C, Louis-Brennetot C, Surdez D, Raynal V, Baulande S, Boeva V, Grossetête-Lalami S, Combaret V, Peuchmaur M, (2018). Activated ALK signals through the ERK-ETV5-RET pathway to drive neuroblastoma oncogenesis. Oncogene 37, 1417–1429. 10.1038/s41388-017-0039-5. 29321660 PMC6168456

[R40] Matthay KK, Reynolds CP, Seeger RC, Shimada H, Adkins ES, Haas-Kogan D, Gerbing RB, London WB, and Villablanca JG (2009). Long-term results for children with high-risk neuroblastoma treated on a randomized trial of myeloablative therapy followed by 13-cis-retinoic acid: a children’s oncology group study. J. Clin. Oncol 27, 1007–1013. 10.1200/jco.2007.13.8925. 19171716 PMC2738615

[R41] McGinty L, and Kolesar J (2017). Dinutuximab for maintenance therapy in pediatric neuroblastoma. Am. J. Health Syst. Pharm 74, 563–567. 10.2146/ajhp160228. 28389455

[R42] Méndez-Martínez S, Calvo P, Ruiz-Moreno O, Pardiñas Barón N, Leciñena Bueno J, Gil Ruiz MDR, and Pablo L (2019). Ocular adverse events associated with MEK inhibitors. Retina 39, 1435–1450. 10.1097/iae.0000000000002451. 30681641

[R43] Misale S, Fatherree JP, Cortez E, Li C, Bilton S, Timonina D, Myers DT, Lee D, Gomez-Caraballo M, Greenberg M, (2019). KRAS G12C NSCLC models are sensitive to direct targeting of KRAS in combination with PI3K inhibition. Clin. Cancer Res 25, 796–807. 10.1158/1078-0432.ccr-18-0368. 30327306

[R44] Montagner A, Yart A, Dance M, Perret B, Salles JP, and Raynal P (2005). A novel role for Gab1 and SHP2 in epidermal growth factor-induced Ras activation. J. Biol. Chem 280, 5350–5360. 10.1074/jbc.m410012200. 15574420

[R45] Montero-Conde C, Ruiz-Llorente S, Dominguez JM, Knauf JA, Viale A, Sherman EJ, Ryder M, Ghossein RA, Rosen N, and Fagin JA (2013). Relief of feedback inhibition of HER3 transcription by RAF and MEK inhibitors attenuates their antitumor effects in BRAF-mutant thyroid carcinomas. Cancer Discov. 3, 520–533. 10.1158/2159-8290.cd-12-0531. 23365119 PMC3651738

[R46] Morris EJ, Jha S, Restaino CR, Dayananth P, Zhu H, Cooper A, Carr D, Deng Y, Jin W, Black S, (2013). Discovery of a novel ERK inhibitor with activity in models of acquired resistance to BRAF and MEK inhibitors. Cancer Discov. 3, 742–750. 10.1158/2159-8290.cd-13-0070. 23614898

[R47] Mourad N, Lourenço N, Delyon J, Eftekhari P, Bertheau P, Allayous C, Ballon A, Pagès C, Allez M, Lebbé C, and Baroudjian B; PATIO group (2019). Severe gastrointestinal toxicity of MEK inhibitors. Melanoma Res. 29, 556–559. 10.1097/CMR.0000000000000618. 31095035

[R48] Nichols RJ, Haderk F, Stahlhut C, Schulze CJ, Hemmati G, Wildes D, Tzitzilonis C, Mordec K, Marquez A, Romero J, (2018). RAS nucleotide cycling underlies the SHP2 phosphatase dependence of mutant BRAF-NF1- and RAS-driven cancers. Nat. Cell Biol 20, 1064–1073. 10.1038/s41556-018-0169-1. 30104724 PMC6115280

[R49] Padovan-Merhar OM, Raman P, Ostrovnaya I, Kalletla K, Rubnitz KR, Sanford EM, Ali SM, Miller VA, Mossé YP, Granger MP, (2016). Enrichment of targetable mutations in the relapsed neuroblastoma genome. PLoS Genet. 12, e1006501. 10.1371/journal.pgen.1006501. 27997549 PMC5172533

[R50] Pugh TJ, Morozova O, Attiyeh EF, Asgharzadeh S, Wei JS, Auclair D, Carter SL, Cibulskis K, Hanna M, Kiezun A, (2013). The genetic landscape of high-risk neuroblastoma. Nat. Genet 45, 279–284. 10.1038/ng.2529. 23334666 PMC3682833

[R51] Ran H, Tsutsumi R, Araki T, and Neel BG (2016). Sticking it to cancer with molecular glue for SHP2. Cancer Cell 30, 194–196. 10.1016/j.ccell.2016.07.010. 27505669 PMC5558882

[R52] Rinehart J, Adjei AA, Lorusso PM, Waterhouse D, Hecht JR, Natale RB, Hamid O, Varterasian M, Asbury P, Kaldjian EP, (2004). Multicenter phase II study of the oral MEK inhibitor, CI-1040, in patients with advanced non-small-cell lung, breast, colon, and pancreatic cancer. J. Clin. Oncol 22, 4456–4462. 10.1200/jco.2004.01.185. 15483017

[R53] Ruess DA, Heynen GJ, Ciecielski KJ, Ai J, Berninger A, Kabacaoglu D, Görgülü K, Dantes Z, Wörmann SM, Diakopoulos KN, (2018). Mutant KRAS-driven cancers depend on PTPN11/SHP2 phosphatase. Nat. Med 24, 954–960. 10.1038/s41591-018-0024-8. 29808009

[R54] Sale MJ, and Cook SJ (2013). The BH3 mimetic ABT-263 synergizes with the MEK1/2 inhibitor selumetinib/AZD6244 to promote BIM-dependent tumour cell death and inhibit acquired resistance. Biochem. J 450, 285–294. 10.1042/bj20121212. 23234544

[R55] Sano R, Krytska K, Larmour CE, Raman P, Martinez D, Ligon GF, Lillquist JS, Cucchi U, Orsini P, Rizzi S, (2019a). An antibody-drug conjugate directed to the ALK receptor demonstrates efficacy in preclinical models of neuroblastoma. Sci. Transl. Med 11, eaau9732. 10.1126/scitranslmed.aau9732. 30867324 PMC10023134

[R56] Sano S, Oe K, Fukui T, Hayashi S, Kuroda R, and Niikura T (2019b). Humeral shaft non-union in a patient with osteogenesis imperfecta treated with mandible locking plate fixation: a case report. J. Orthop. Case Rep 9, 19–21. 10.13107/jocr.2250-0685.1400. PMC6742884 31559219

[R57] Sarbassov DD, Guertin DA, Ali SM, and Sabatini DM (2005). Phosphorylation and regulation of Akt/PKB by the rictor-mTOR complex. Science 307, 1098–1101. 10.1126/science.1106148. 15718470

[R58] Schoen F, Lochmann M, Prell J, Herfurth K, and Rampp S (2018). Neuronal correlates of product feature attractiveness. Front. Behav. Neurosci 12, 147. 10.3389/fnbeh.2018.00147. 30072882 PMC6059068

[R59] Song KA, Hosono Y, Turner C, Jacob S, Lochmann TL, Murakami Y, Patel NU, Ham J, Hu B, Powell KM, (2018). Increased synthesis of MCL-1 protein underlies initial survival of EGFR-mutant lung cancer to EGFR inhibitors and provides a novel drug target. Clin. Cancer Res 24, 5658–5672. 10.1158/1078-0432.ccr-18-0304. 30087143

[R60] Stjepanovic N, Velazquez-Martin JP, and Bedard PL (2016). Ocular toxicities of MEK inhibitors and other targeted therapies. Ann. Oncol 27, 998–1005. 10.1093/annonc/mdw100. 26951625

[R61] Turke AB, Song Y, Costa C, Cook R, Arteaga CL, Asara JM, and Engelman JA (2012). MEK inhibition leads to PI3K/AKT activation by relieving a negative feedback on ERBB receptors. Cancer Res. 72, 3228–3237. 10.1158/0008-5472.can-11-3747. 22552284 PMC3515079

[R62] Umapathy G, Guan J, Gustafsson DE, Javanmardi N, Cervantes-Madrid D, Djos A, Martinsson T, Palmer RH, and Hallberg B (2017). MEK inhibitor trametinib does not prevent the growth of anaplastic lymphoma kinase (ALK)-addicted neuroblastomas. Sci. Signal 10, eaam7550. 10.1126/scisignal.aam7550. 29184034

[R63] Valencia-Sama I, Ladumor Y, Kee L, Adderley T, Christopher G, Robinson CM, Kano Y, Ohh M, and Irwin MS (2020). NRAS status determines sensitivity to SHP2 inhibitor combination therapies targeting the RAS-MAPK pathway in neuroblastoma. Cancer Res. 80, 3413–3423. 10.1158/0008-5472.can-19-3822. 32586982

[R64] Welsh SJ, and Corrie PG (2015). Management of BRAF and MEK inhibitor toxicities in patients with metastatic melanoma. Ther. Adv. Med. Oncol 7, 122–136. 10.1177/1758834014566428. 25755684 PMC4346212

[R65] Wong GS, Zhou J, Liu JB, Wu Z, Xu X, Li T, Xu D, Schumacher SE, Puschhof J, McFarland J, (2018). Targeting wild-type KRAS-amplified gastroesophageal cancer through combined MEK and SHP2 inhibition. Nat. Med 24, 968–977. 10.1038/s41591-018-0022-x. 29808010 PMC6039276

[R66] Yang W, Soares J, Greninger P, Edelman EJ, Lightfoot H, Forbes S, Bindal N, Beare D, Smith JA, Thompson IR, Ramaswamy S, Futreal PA, Haber DA, Stratton MR, Benes C, McDermott U, and Garnett MJ (2013). Genomics of Drug Sensitivity in Cancer (GDSC): a resource for therapeutic biomarker discovery in cancer cells. Nucleic Acids Res. 41, D955–D961. 10.1093/nar/gks1111. 23180760 PMC3531057

[R67] Yu AL, Gilman AL, Ozkaynak MF, London WB, Kreissman SG, Chen HX, Smith M, Anderson B, Villablanca JG, Matthay KK, (2010). Anti-GD2 antibody with GM-CSF, interleukin-2, and isotretinoin for neuroblastoma. N. Engl. J. Med 363, 1324–1334. 10.1056/nejmoa0911123. 20879881 PMC3086629

[R68] Zhang SQ, Yang W, Kontaridis MI, Bivona TG, Wen G, Araki T, Luo J, Thompson JA, Schraven BL, Philips MR, and Neel BG (2004). Shp2 regulates SRC family kinase activity and Ras/Erk activation by controlling Csk recruitment. Mol. Cell 13, 341–355. 10.1016/s1097-2765(04)00050-4. 14967142

[R69] Zhang X, Dong Z, Zhang C, Ung CY, He S, Tao T, Oliveira AM, Meves A, Ji B, Look AT, (2017). Critical role for GAB2 in neuroblastoma pathogenesis through the promotion of SHP2/MYCN cooperation. Cell Rep. 18, 2932–2942. 10.1016/j.celrep.2017.02.065. 28329685 PMC5393048

