# Supplementary material for: High-risk neuroblastoma with NF1 loss of function is targetable using SHP2 inhibition
Source: Cell Rep. Author manuscript; Available in PMC 2023 Jul 19. (PMC10353975; doi:10.1016/j.celrep.2022.111095)
Supplement: 1 [file NIHMS1914136-supplement-1.pdf]

Cell Reports, Volume *40*

Supplemental information

High-risk neuroblastoma with NF1 loss of function is targetable using SHP2 inhibition

Jinyang Cai, Sheeba Jacob, Richard Kurupi, Krista M. Dalton, Colin Coon, Patricia Greninger, Regina K. Egan, Giovanna T. Stein, Ellen Murchie, Joseph McClanaghan, Yuta Adachi, Kentaro Hirade, Mikhail Dozmorov, John Glod, Sosipatros A. Boikos, Hiromichi Ebi, Huaixiang Hao, Giordano Caponigro, Cyril H. Benes, and Anthony C. Faber

Sup. Fig. 1

A

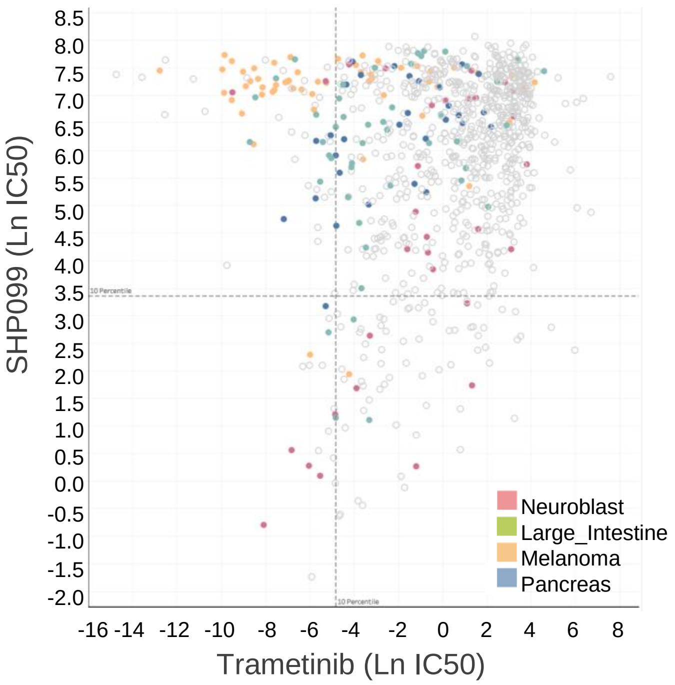

8.5
8.0
7.5
7.0
6.5
6.0
5.5
5.0
4.5
4.0
3.5
3.0
2.5
2.0
1.5
1.0
0.5
0.0
-0.5
-1.0
-1.5
-2.0
SHP099 (Ln IC50)
-16 -14 -12 -10 -8 -6 -4 -2 0 2 4 6 8
Trametinib (Ln IC50)
Neuroblast
Large_Intestine
Melanoma
Pancreas

B

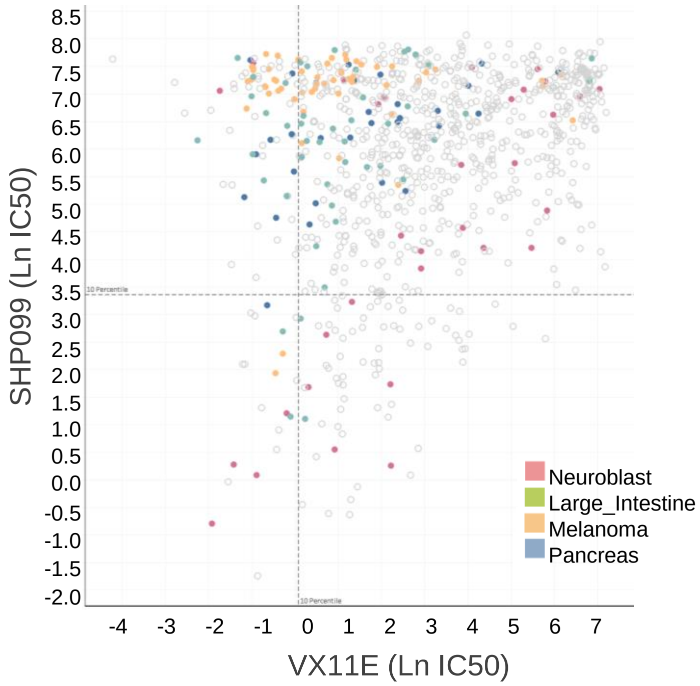

8.5
8.0
7.5
7.0
6.5
6.0
5.5
5.0
4.5
4.0
3.5
3.0
2.5
2.0
1.5
1.0
0.5
0.0
-0.5
-1.0
-1.5
-2.0
SHP099 (Ln IC50)
-4 -3 -2 -1 0 1 2 3 4 5 6 7
VX11E (Ln IC50)
Neuroblast
Large_Intestine
Melanoma
Pancreas

Sup Fig.1. Graphs represent differential activity of SHP099 (IC50) with respect to IC50 of trametinib (A) and VX11E (B) for each cell line (n=922; 32 NB cell lines). Dotted lines correspond to top 10% sensitive cell line for each drug. Related to Figure 1.

Sup. Fig. 2

A

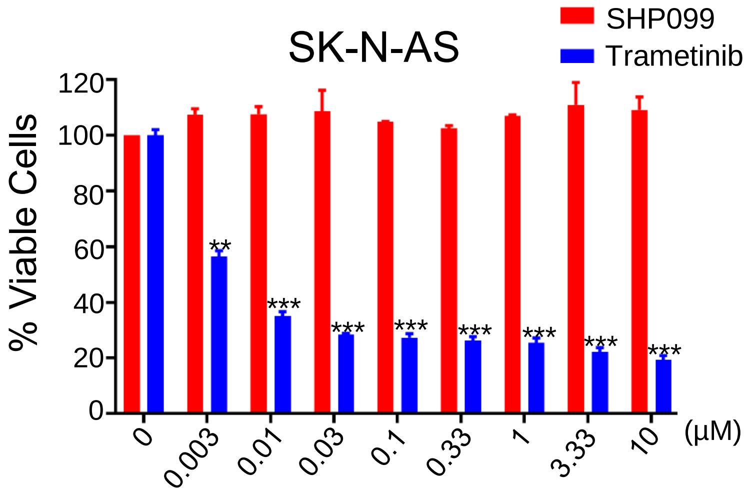

SK-N-AS
SHP099
Trametinib
% Viable Cells
120
100
80
60
40
20
0
**
***
0
0.003
0.01
0.03
0.1
0.33
1
3.33
10
(μM)

B

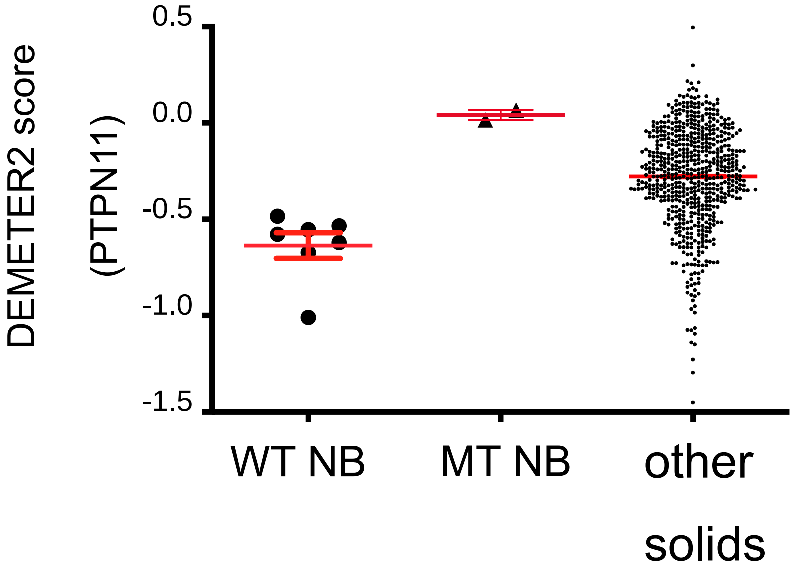

DEMETER2 score
(PTPN11)
0.5
0.0
-0.5
-1.0
-1.5
WT NB
MT NB
other
solids

Sup Fig.2. Trametinib sensitivity in an *NRAS* MT NB cell line. A) Graph represents percent viable cells assessed by Cell Titer-Glo in the *NRAS* MT SK-N-AS cell line following 7 days treatment with increasing concentrations (0.003-10 µM) of trametinib or SHP099. Error bars are ±SEM. Three individual sets of experiments were performed. B) PTPN11 (SHP2) RNAi screens (Achilles+DRIVE+Marcotte) DEMETER2 scores from DepMap. NB RAS/RAF WT n=7, NB RAS/RAF MT n=2, other tumors, n= 639. WT NB versus other tumors, $p<0.0001$ by Mann-Whitney. Related to Figure 1.

Sup. Fig. 3

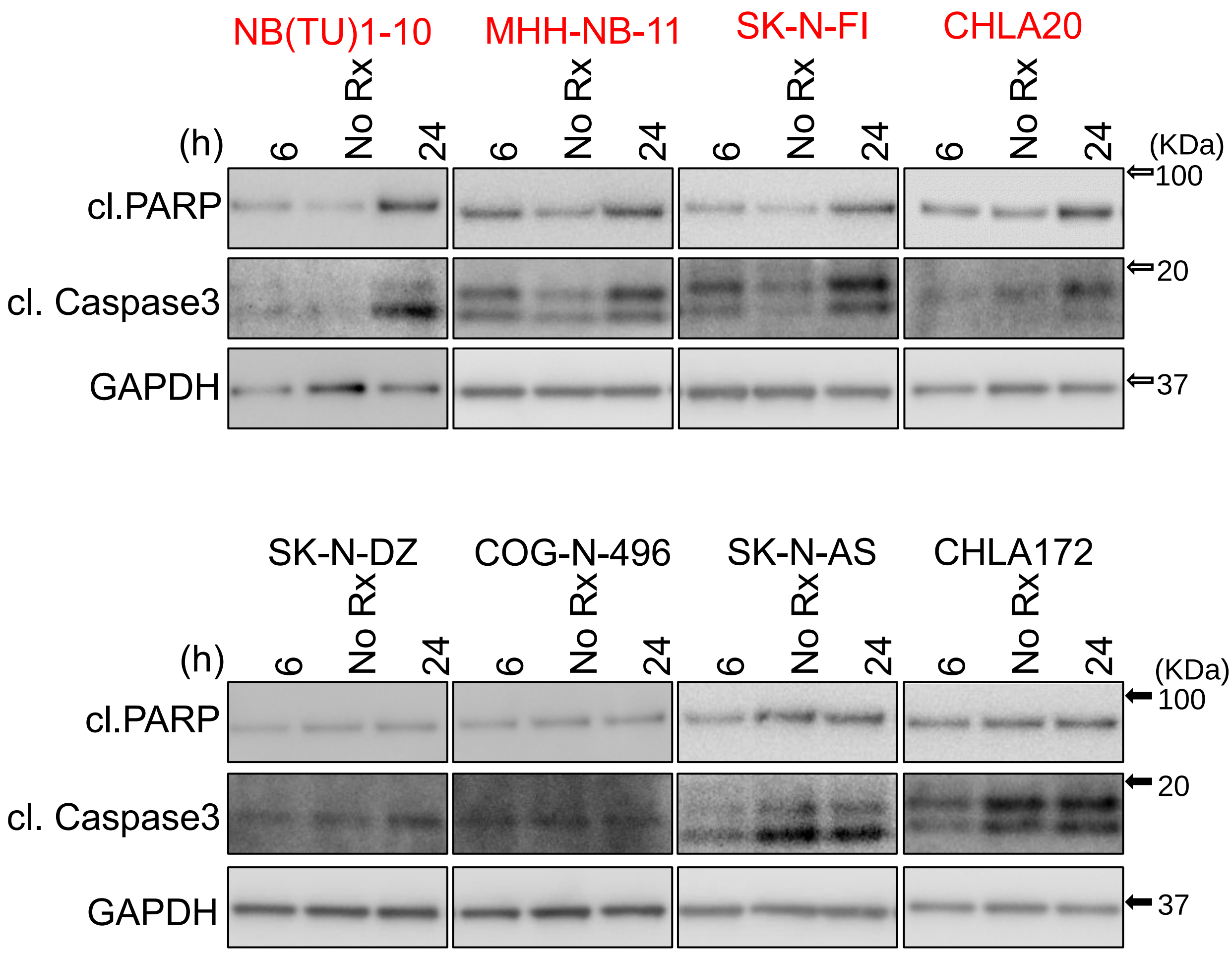
NB(TU)1-10
MHH-NB-11
SK-N-FI
CHLA20
(h)
6
No Rx
24
(KDa)
cl.PARP
cl. Caspase3
GAPDH
100
20
37
SK-N-DZ
COG-N-496
SK-N-AS
CHLA172

Sup Fig.3. Viability effects were in part due to cell death. Immunoblot of the NB cell lines indicated, treated with SHP099 (5 µM) for 6h and 24h, respectively, or untreated (No Rx), and assessed with the indicated antibodies. Related to Figure 1.

Sup .Fig. 4

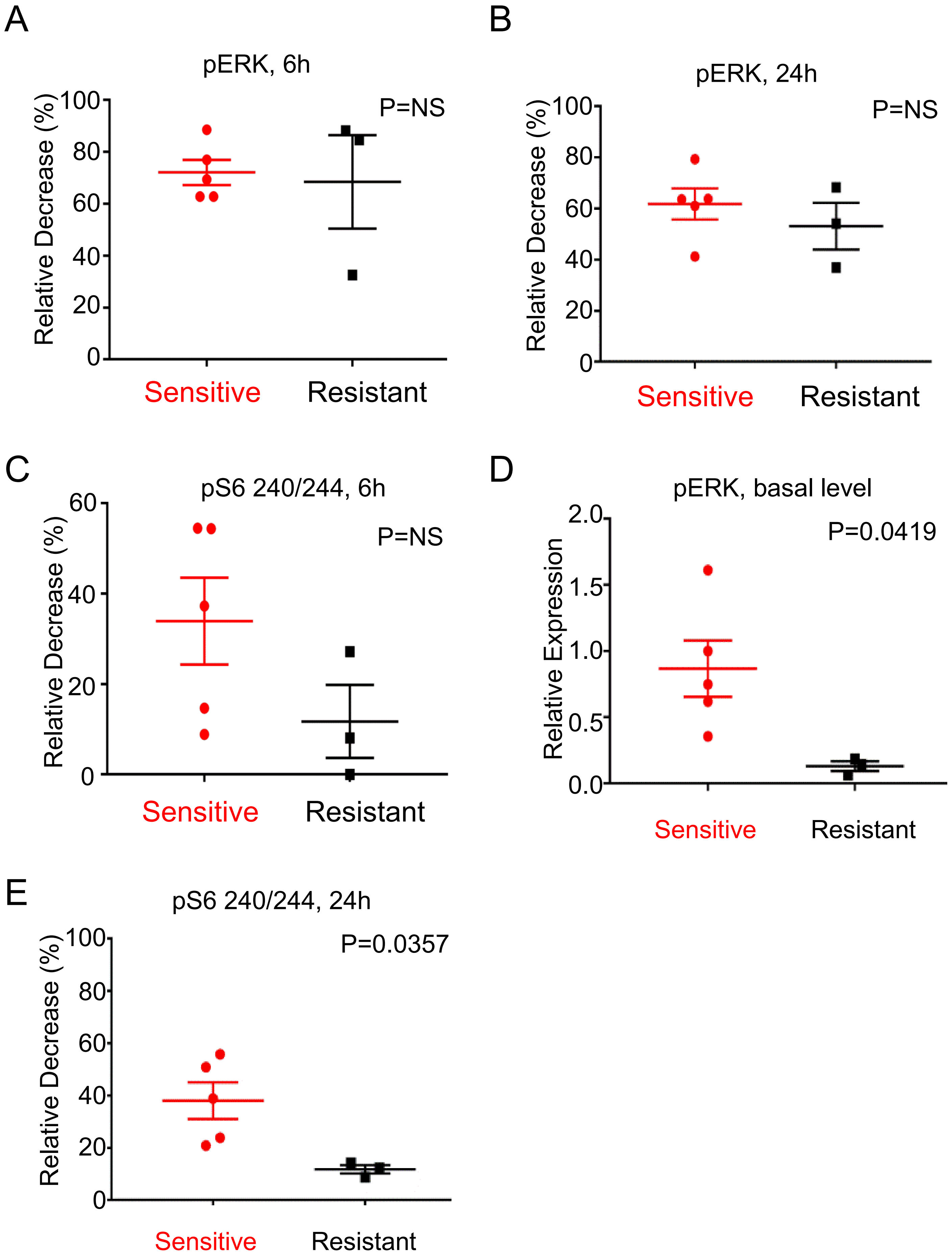
A
pERK, 6h
P=NS
Relative Decrease (%)
100
80
60
40
20
0
Sensitive
Resistant
B
pERK, 24h
P=NS
Relative Decrease (%)
100
80
60
40
20
0
Sensitive
Resistant
C
pS6 240/244, 6h
P=NS
Relative Decrease (%)
60
40
20
0
Sensitive
Resistant
D
pERK, basal level
P=0.0419
Relative Expression
2.0
1.5
1.0
0.5
0.0
Sensitive
Resistant
E
pS6 240/244, 24h
P=0.0357
Relative Decrease (%)
100
80
60
40
20
0
Sensitive
Resistant

Sup Fig.4. Relative levels of pERK and pS6 in SHP099 sensitive and insensitive NB cell lines. (A) Relative levels of pERK in SHP099 sensitive and insensitive lines normalized to GAPDH. (B) Relative percent decrease of pERK expression in SHP099 sensitive and resistant NB cell lines treated with SHP099 for 24h. (C) Relative percent decrease of pS6 in SHP099 sensitive and resistant NB cell lines treated with SHP099 for 6h. (D) Relative expression of pERK basal level in SHP099 sensitive (red) and resistant (black) NB cell lines compared to untreated (No Rx) cells. (E) Relative percentage decrease of pS6 (240/244) in SHP099 sensitive (red) and resistant (black) NB cell lines compared to untreated (No Rx), quantified from figure 2A. Related to Figure 2.

Sup. Fig. 5

A

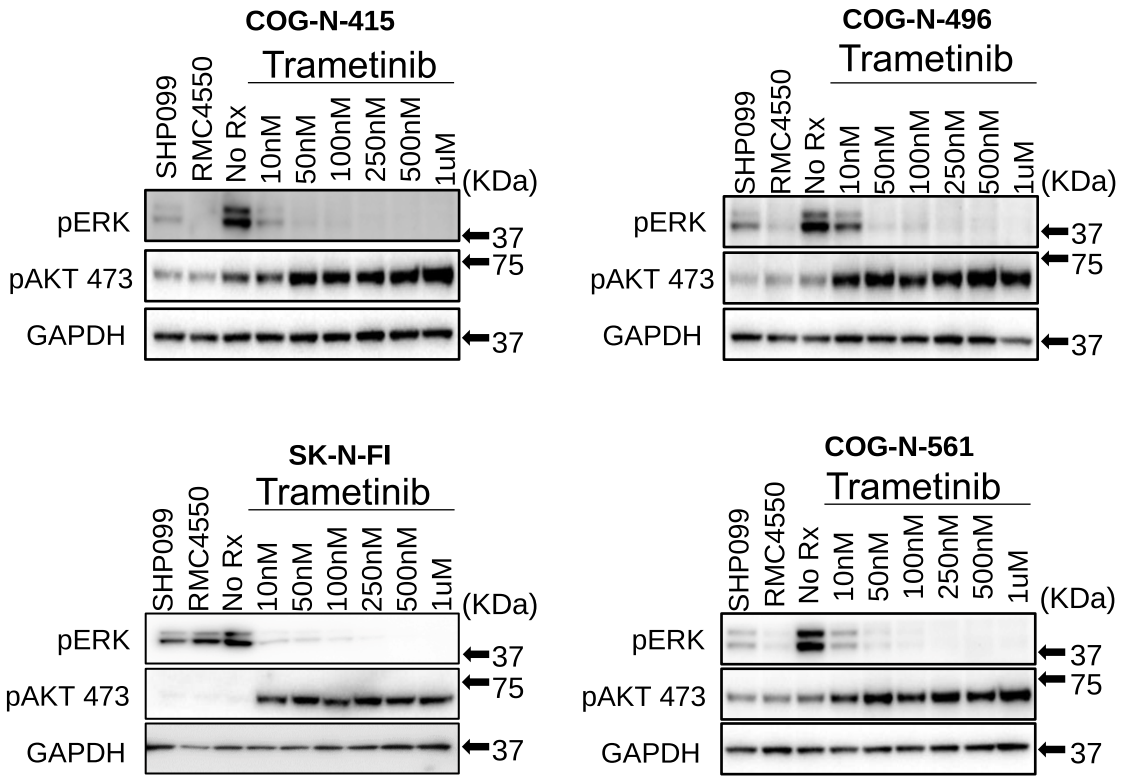
COG-N-415
Trametinib
SHP099
RMC4550
No Rx
10nM
50nM
100nM
250nM
500nM
1uM
(KDa)
pERK
pAKT 473
GAPDH
37
75
37
COG-N-496
Trametinib
SHP099
RMC4550
No Rx
10nM
50nM
100nM
250nM
500nM
1uM
(KDa)
pERK
pAKT 473
GAPDH
37
75
37
SK-N-FI
Trametinib
SHP099
RMC4550
No Rx
10nM
50nM
100nM
250nM
500nM
1uM
(KDa)
pERK
pAKT 473
GAPDH
37
75
37
COG-N-561
Trametinib
SHP099
RMC4550
No Rx
10nM
50nM
100nM
250nM
500nM
1uM
(KDa)
pERK
pAKT 473
GAPDH
37
75
37

B

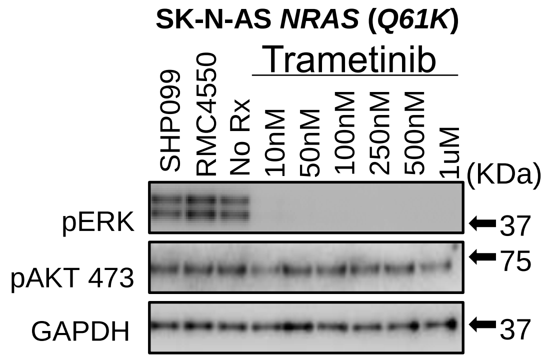
SK-N-AS *NRAS* (*Q61K*)
Trametinib
SHP099
RMC4550
No Rx
10nM
50nM
100nM
250nM
500nM
1uM
(KDa)
pERK
pAKT 473
GAPDH
37
75
37

Sup Fig.5. Immunoblot analysis of NRAS WT and MT NB cell lines treated with SHP099 (5 µM) or RMC-4550 (10 µM) or trametinib. A) Immunoblot analysis of NB PDX lines COG-N-415, COG-N-496, COG-N-561 and SK-N-FI treated with SHP099 (5 µM) or RMC-4550 (10 µM) or trametinib (0.01 µM-1 µM) for 24h and untreated control (No Rx) cells assessed with the indicated antibodies. GAPDH was used as loading control. B) Immunoblot analysis of NRAS mutant SK-N-AS cells treated with SHP099 (5 µM) or RMC-4550 (10 µM) or trametinib (0.01 µM-1 µM) for 24h and untreated control (No Rx) cells were assessed with the indicated antibodies. GAPDH was used as loading control. Related to Figure 2.

Sup Fig. 6

A

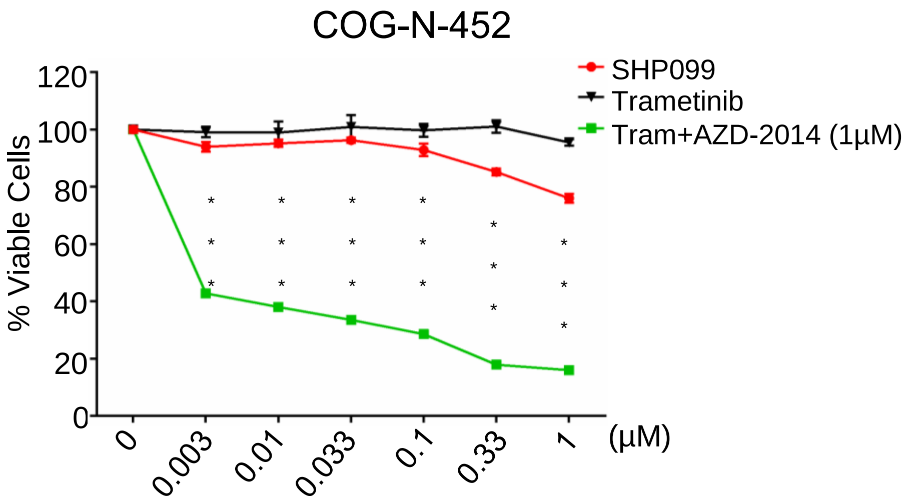
COG-N-452
SHP099
Trametinib
Tram+AZD-2014 (1µM)
% Viable Cells
120
100
80
60
40
20
0
0
0.003
0.01
0.033
0.1
0.33
1
(µM)

B

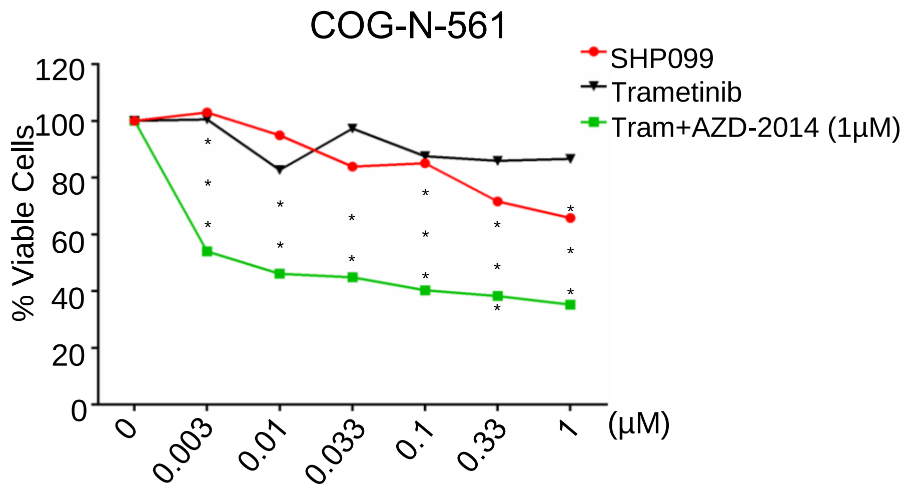
COG-N-561
SHP099
Trametinib
Tram+AZD-2014 (1µM)
% Viable Cells
120
100
80
60
40
20
0
0
0.003
0.01
0.033
0.1
0.33
1
(µM)

C

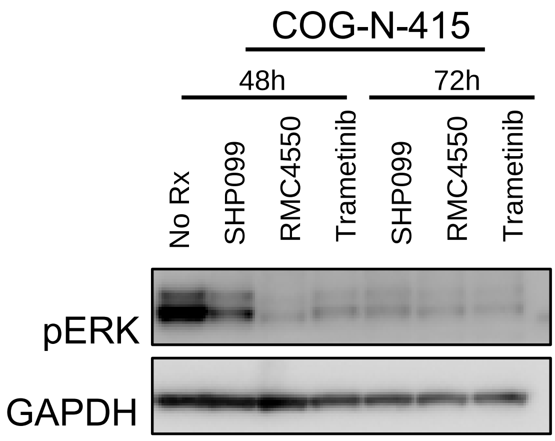
COG-N-415
48h
72h
No Rx
SHP099
RMC4550
Trametinib
SHP099
RMC4550
Trametinib
pERK
GAPDH

Sup Fig.6. mTOR inhibitor AZD-2014 sensitizes NB lines to trametinib. Percent viable cells assessed by Cell Titer-Glo in the (A) COG-N-452 and (B) COG-N-561 cell lines following 72h treatment with increasing concentrations (0.003-10 µM) of SHP099, trametinib, AZD-2014 and trametinib in combination with 1 µM AZD-2014. Error bars are ±SEM. Three individual sets of experiments were performed. (C) Immunoblot analysis of pERK in the COG-N-415 cell line treated with SHP099 (5 µM), RMC-4550 (10 µM) or trametinib (1 µM) for 48h and 72h. Related to Figure 2.

Sup. Fig. 7

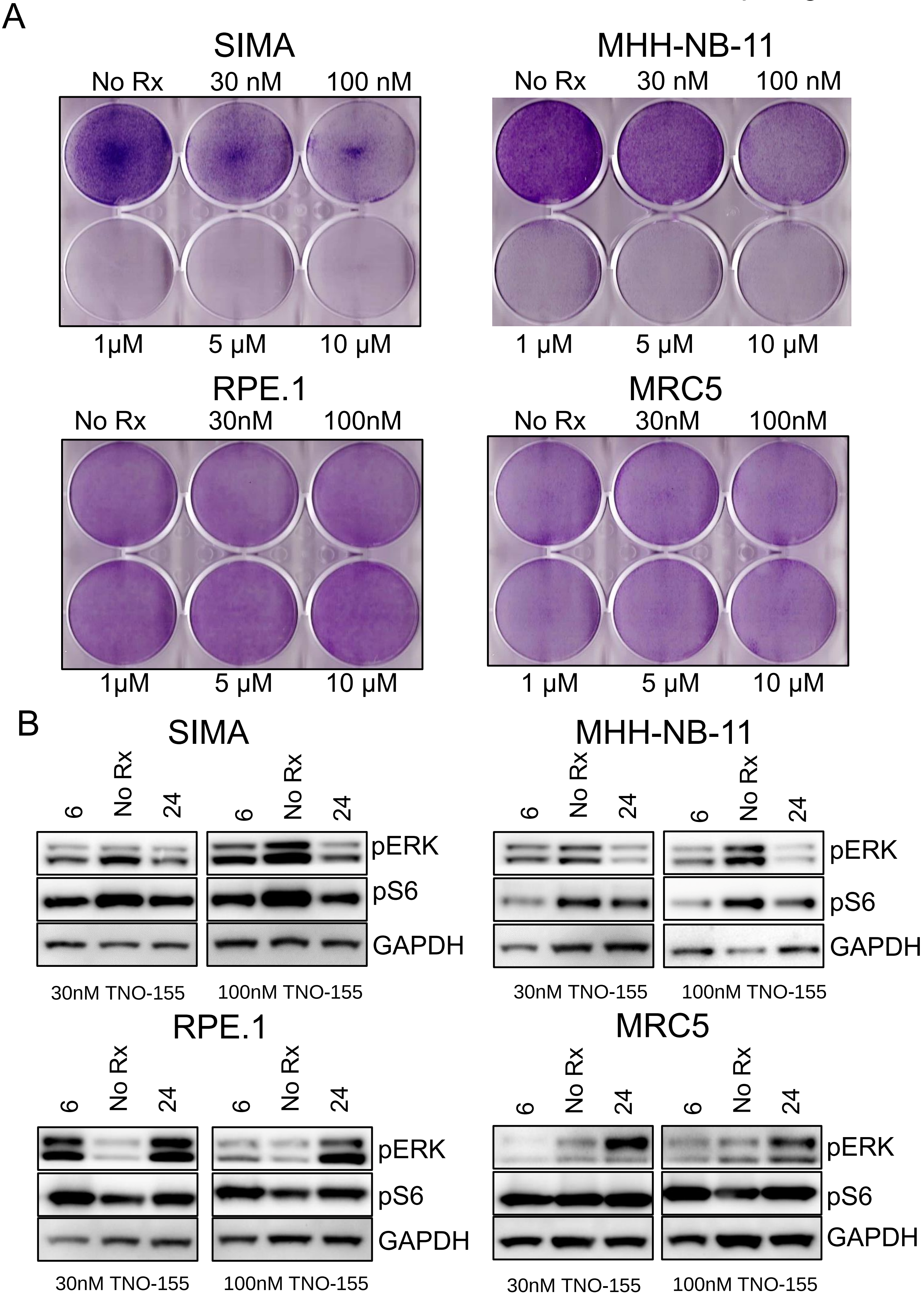
A
SIMA
No Rx
30 nM
100 nM
1μM
5 μM
10 μM
MHH-NB-11
No Rx
30 nM
100 nM
1 μM
5 μM
10 μM
RPE.1
No Rx
30nM
100nM
1μM
5 μM
10 μM
MRC5
No Rx
30nM
100nM
1 μM
5 μM
10 μM
B
SIMA
6
No Rx
24
pERK
pS6
GAPDH
30nM TNO-155
100nM TNO-155
MHH-NB-11
6
No Rx
24
pERK
pS6
GAPDH
30nM TNO-155
100nM TNO-155
RPE.1
6
No Rx
24
pERK
pS6
GAPDH
30nM TNO-155
100nM TNO-155
MRC5
6
No Rx
24
pERK
pS6
GAPDH
30nM TNO-155
100nM TNO-155

Sup Fig.7. TNO-155 show similar sensitivity to SHP099 sensitive in both NB cell lines and normal tissue-derived cell lines. (A) Crystal violet assays or (B) immunoblot analysis of SIMA and MHH-NB-11 NB cell lines and RPE.1 and MRC5 normal tissue-derived cell lines treated with vehicle (No Rx), and for (A), increasing TNO-155 from 30 nM to 10 µM until the untreated (No Rx) cells reached confluency. Experiments were performed in triplicates. (B) Immunoblot analysis of NB cell lines SIMA, MHH-NB-11 and the normal tissue-derived cell lines RPE.1and MRC5 treated with TNO-155 (30 nM or 100 nM) for 6h or 24h and untreated control (No Rx) cells assessed with the indicated antibodies. GAPDH was used as loading control. For (A), experiments were performed in triplicates. Related to Figure 2.

Sup Fig.8

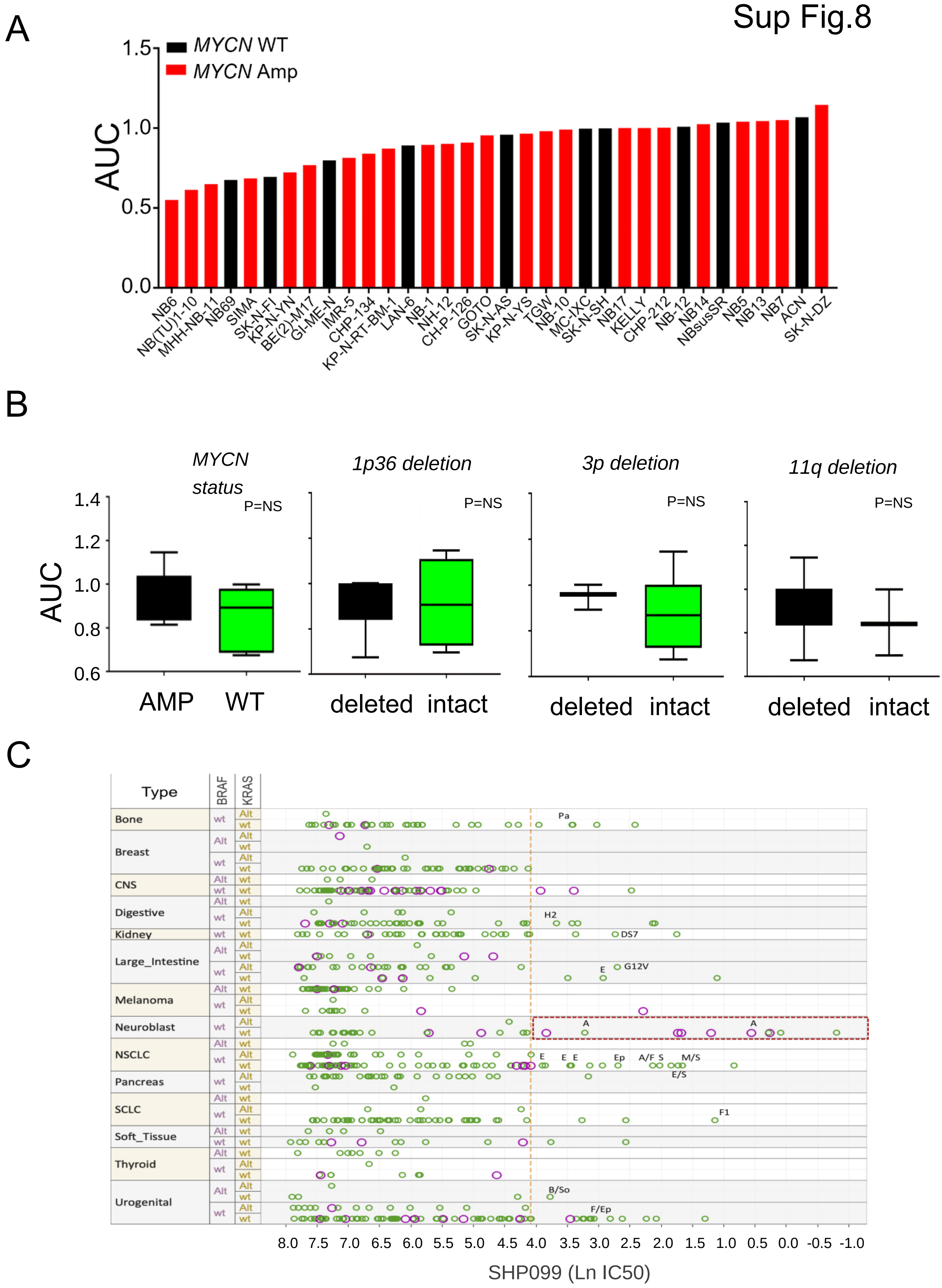
A
MYCN WT
MYCN Amp
AUC
1.5
1.0
0.5
0.0
NB6
NB(TU)1-10
MHH-NB-11
NB69
SIMA
SK-N-FI
KP-N-YN
BE(2)-M17
GI-ME-N
IMR-5
CHP-134
KP-N-RT-BM-1
LAN-6
NB-1
NH-12
CH-P-126
GOTO
SK-N-AS
KP-N-YS
TGW
NB-10
MC-IXC
SK-N-SH
NB17
KELLY
CHP-212
NB-12
NB14
NBsusSR
NB5
NB13
NB7
ACN
SK-N-DZ
B
MYCN status
1p36 deletion
3p deletion
11q deletion
P=NS
AUC
1.4
1.2
1.0
0.8
0.6
AMP
WT
deleted
intact
C
Type
BRAF
KRAS
Bone
Breast
CNS
Digestive
Kidney
Large_Intestine
Melanoma
Neuroblast
NSCLC
Pancreas
SCLC
Soft_Tissue
Thyroid
Urogenital
Alt
wt
Pa
H2
DS7
E
G12V
A
E E E
Ep
A/F S
M/S
E/S
F1
B/So
F/Ep
8.0 7.5 7.0 6.5 6.0 5.5 5.0 4.5 4.0 3.5 3.0 2.5 2.0 1.5 1.0 0.5 0.0 -0.5 -1.0
SHP099 (Ln IC50)

Sup Fig 8. SHP099 sensitivity does not correlate with typical genomic alterations in NB. A) Area under curve (AUC) of SHP099 in NB cell lines assayed in HTS. Amplification status of MYCN are indicated as MYCN amplified (red bars) and MYCN-WT (black bars). B) AUC of SHP099 compared to MYCN status (WT and amplified) and various deletions in NB cell lines. Mann-Whitney test was performed to calculate statistical significance between the groups (NS- nonsignificant). C) SHP099 sensitivity correlates with NF1 mutational status in NB. Activity of SHP099 across different tissue types including 32 NB cell lines. The orange dotted line corresponds to the top 10% sensitive cell lines. Mutational status of selected lines are shown: Pa- PDGFRA; H2-HER2 amplified; DS7- DUSP7; E- EGFR; G12V- KRASG12V; A- ALK; Ep- EPHA2; F- FGFR2; S- STK11; M- MET; F1- FGFR1; B- BRAF; So- SOS1. Related to Figure 4.

Sup Fig.9

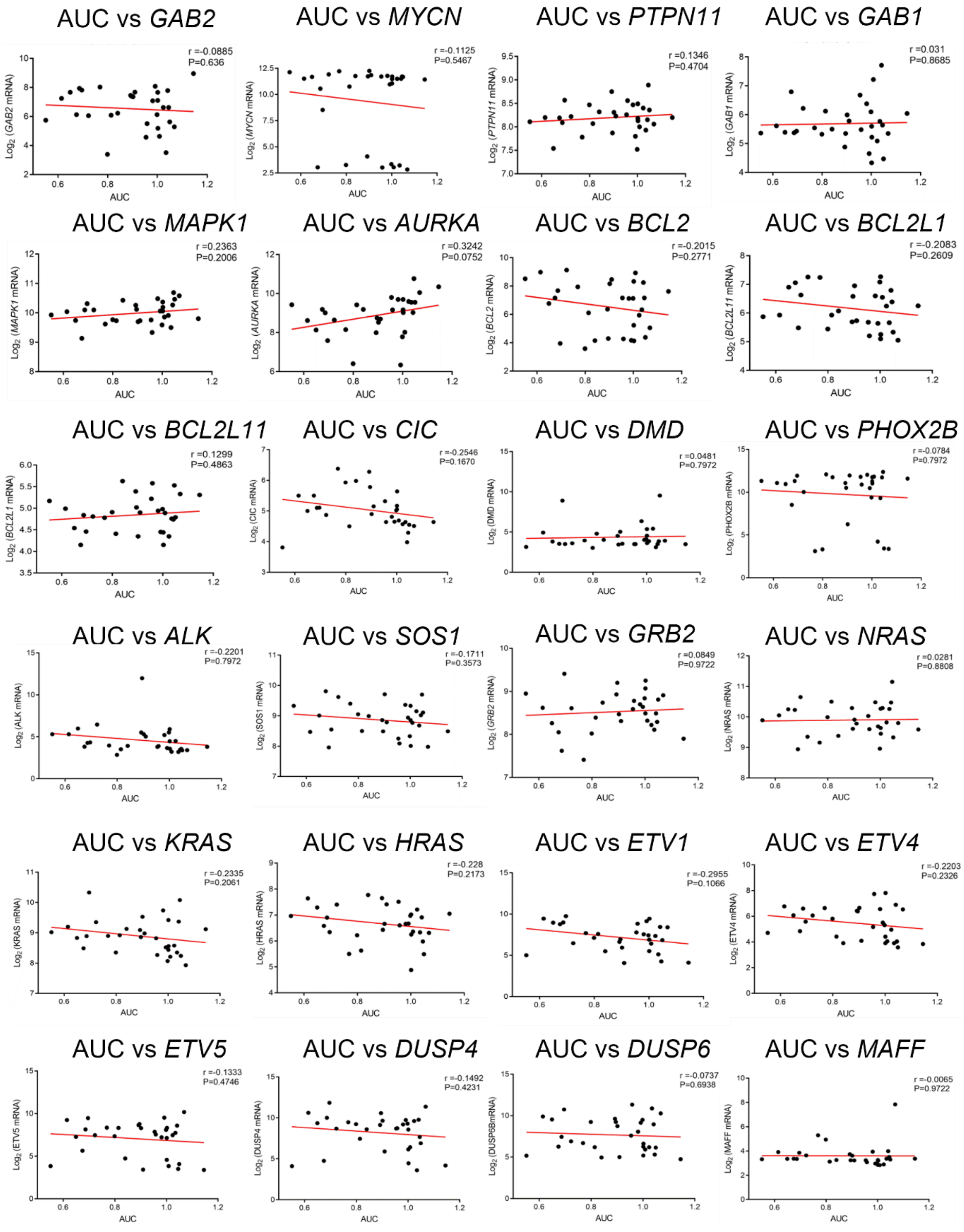

AUC vs GAB2
r =-0.0885
P=0.636
AUC vs MYCN
r =-0.1125
P=0.5467
AUC vs PTPN11
r =0.1346
P=0.4704
AUC vs GAB1
r =0.031
P=0.8685
AUC vs MAPK1
r =0.2363
P=0.2006
AUC vs AURKA
r =0.3242
P=0.0752
AUC vs BCL2
r =-0.2015
P=0.2771
AUC vs BCL2L1
r =-0.2083
P=0.2609
AUC vs BCL2L11
r =0.1299
P=0.4863
AUC vs CIC
r =-0.2546
P=0.1670
AUC vs DMD
r =0.0481
P=0.7972
AUC vs PHOX2B
r =-0.0784
P=0.7972
AUC vs ALK
r =-0.2201
P=0.7972
AUC vs SOS1
r =-0.1711
P=0.3573
AUC vs GRB2
r =0.0849
P=0.9722
AUC vs NRAS
r =0.0281
P=0.8808
AUC vs KRAS
r =-0.2335
P=0.2061
AUC vs HRAS
r =-0.228
P=0.2173
AUC vs ETV1
r =-0.2955
P=0.1066
AUC vs ETV4
r =-0.2203
P=0.2326
AUC vs ETV5
r =-0.1333
P=0.4746
AUC vs DUSP4
r =-0.1492
P=0.4231
AUC vs DUSP6
r =-0.0737
P=0.6938
AUC vs MAFF
r =-0.0065
P=0.9722
AUC

Sup Fig.9. SHP099 sensitivity is not correlated with MEK/ERK expression in NB cell lines. AUC of SHP099 compared to RNA levels of various genes associated with the MEK/ERK pathway. Data obtained from R2 Genomic (Sanger) Database (http://hgserver1.amc.nl). Related to Figure 4.

Sup Fig.10

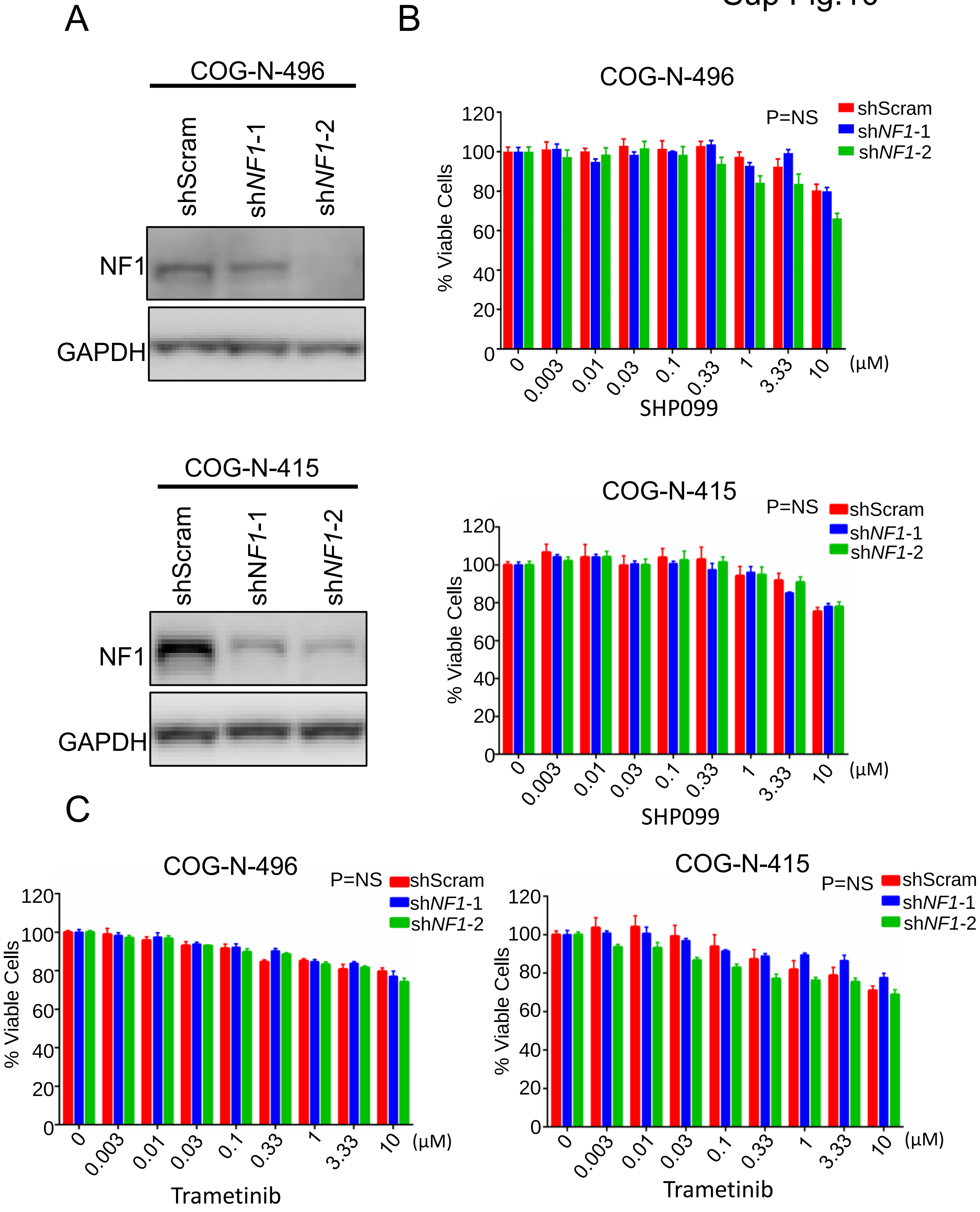
A
B
COG-N-496
shScram
shNF1-1
shNF1-2
NF1
GAPDH
COG-N-415
C
P=NS
% Viable Cells
0
20
40
60
80
100
120
0.003
0.01
0.03
0.1
0.33
1
3.33
10
(µM)
SHP099
Trametinib

Sup Fig.10. NF1 knockdown is not sufficient to confer SHP099 sensitivity in SHP099-insensitive NB cell lines. A) Immunoblot analysis of NF1 knockdown using NF1shRNA in SHP099 insensitive lines: COG-N-496 and COG-N-415. Scrambled shRNA was used for an experimental control and GAPDH as the loading control. (B and C) Percent viable cells assessed by Cell Titer-Glo in NF1 shRNA knockdown and scrambled shRNA (control) COG-N-496 and COG-N-415 cells treated with increasing concentrations (0.003-10 µM) of SHP099 (B) and Trametinib (C) for 72 h. Error bars are ±SEM. Three individual sets of experiments were performed. Related to Figure 4.

Sup Fig.11

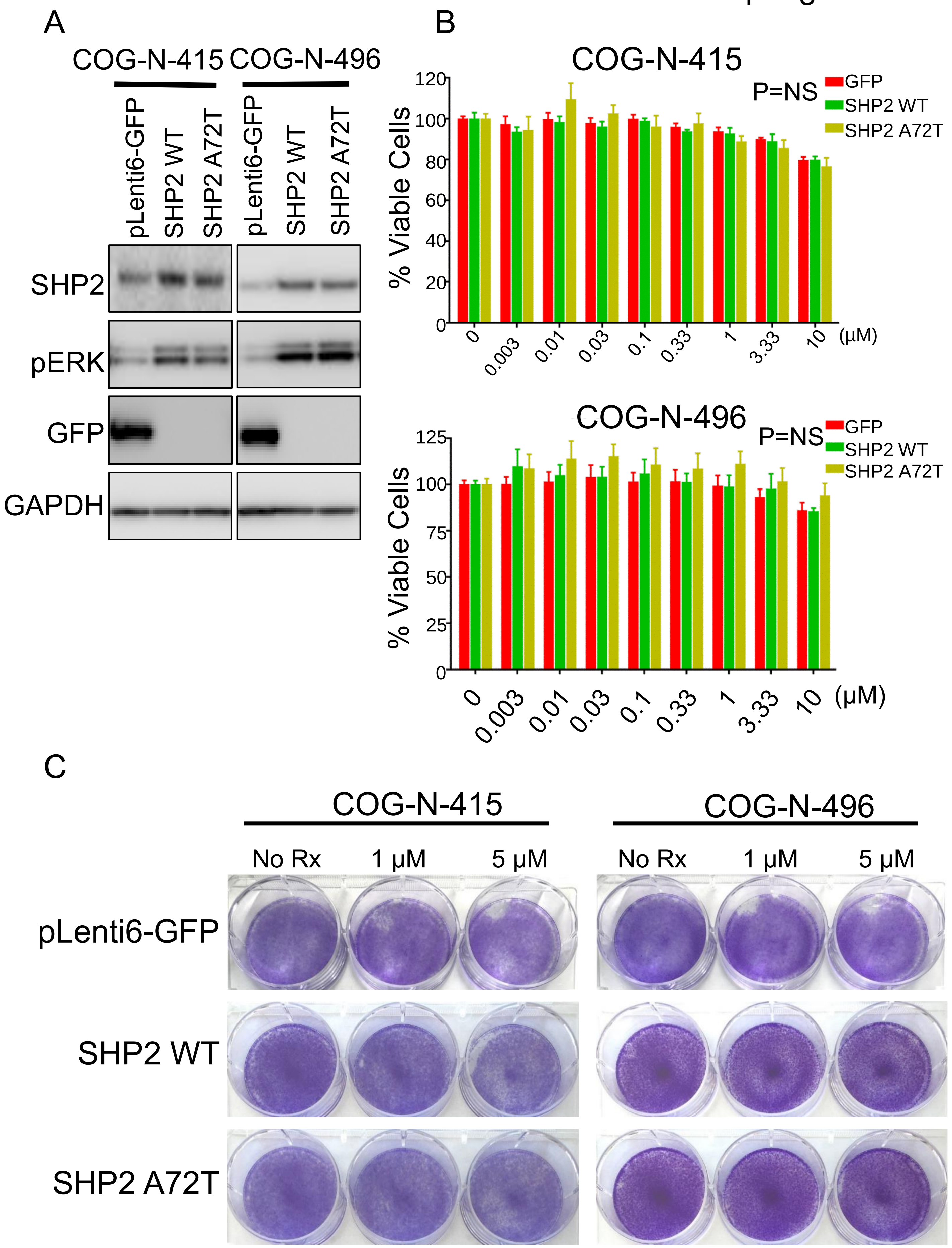
A
COG-N-415
COG-N-496
pLenti6-GFP
SHP2 WT
SHP2 A72T
pLenti6-GFP
SHP2 WT
SHP2 A72T
SHP2
pERK
GFP
GAPDH
B
COG-N-415
P=NS
GFP
SHP2 WT
SHP2 A72T
% Viable Cells
120
100
80
60
40
20
0
0
0.003
0.01
0.03
0.1
0.33
1
3.33
10
(µM)
COG-N-496
P=NS
GFP
SHP2 WT
SHP2 A72T
% Viable Cells
125
100
75
50
25
0
0
0.003
0.01
0.03
0.1
0.33
1
3.33
10
(µM)
C
COG-N-415
COG-N-496
No Rx
1 µM
5 µM
No Rx
1 µM
5 µM
pLenti6-GFP
SHP2 WT
SHP2 A72T

Sup Fig 11. SHP2 mutation (A72T) in NB does not affect SHP099 sensitivity. A) Immunoblot of pLentiGFP, SHP2 WT or SHP2 A72T mutant expressing CO-N-415 and COG-N-496 NB cell lines assessed for SHP2, pERK and GFP expression. GAPDH was used as loading control. B) Graph represents percent viable cells assessed by Cell Titer-Glo in the pLentiGFP, SHP2 WT or SHP2 A72T mutant expressing COG-N-415 and COG-N-496 NB cell lines following 72h treatment with increasing concentrations (0.003-10 µM) of SHP099. Error bars are ±SEM. Three individual sets of experiments were performed. C) Crystal violet assay of pLentiGFP, SHP2 WT or SHP2 A72T mutant expressing COG-N-415 and COG-N-496 NB lines treated with vehicle (No Rx) and SHP099 at 1 µM and 5 µM until the untreated cells (No Rx) reached confluency. Experiments were performed in triplicates. Related to Figure 4.

Sup Fig. 12

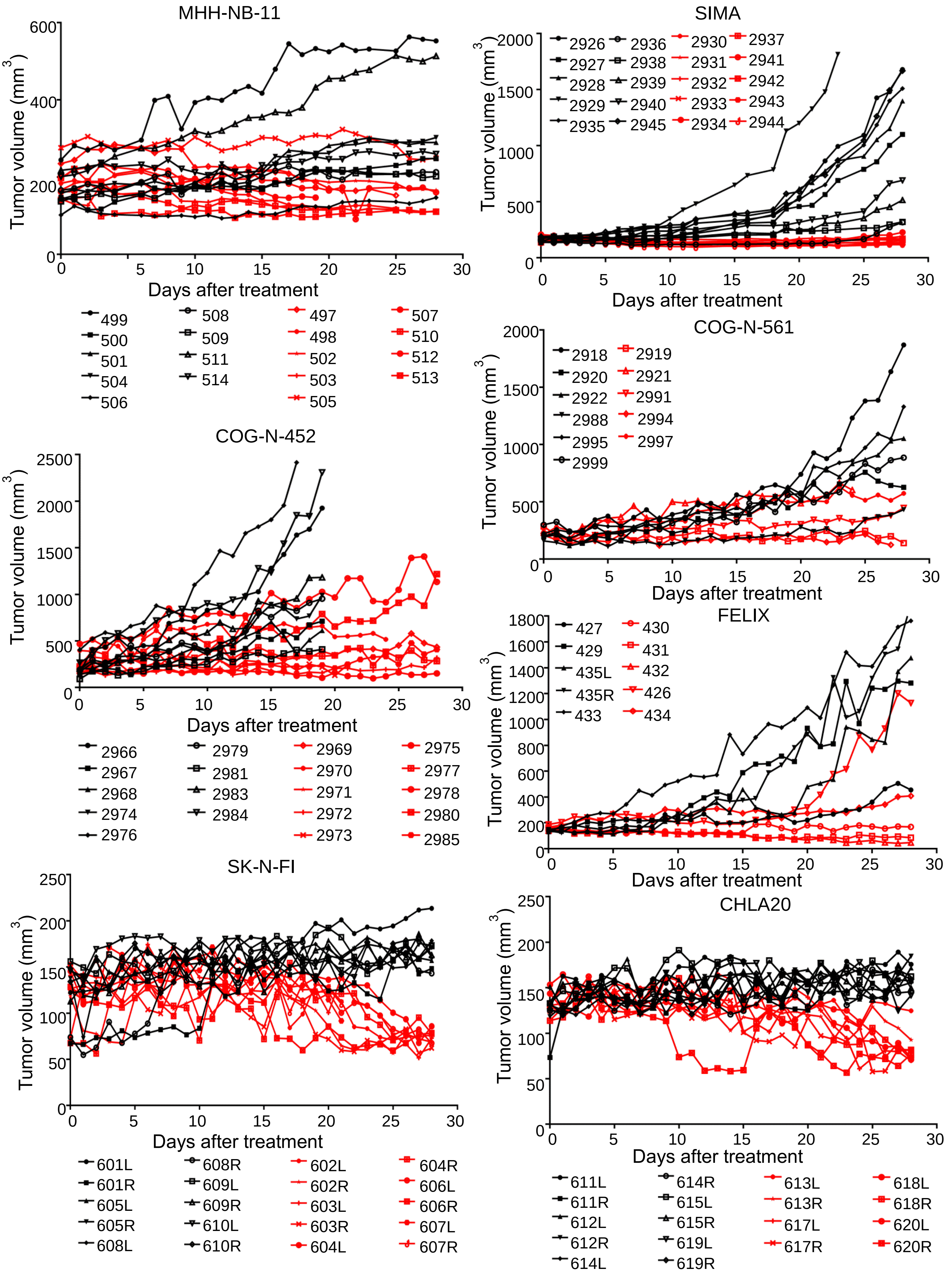
MHH-NB-11
Tumor volume (mm3)
Days after treatment
499
500
501
504
506
508
509
511
514
497
498
502
503
505
507
510
512
513
SIMA
2926
2927
2928
2929
2935
2936
2938
2939
2940
2945
2930
2931
2932
2933
2934
2937
2941
2942
2943
2944
COG-N-561
2918
2920
2922
2988
2995
2999
2919
2921
2991
2994
2997
COG-N-452
2966
2967
2968
2974
2976
2979
2981
2983
2984
2969
2970
2971
2972
2973
2975
2977
2978
2980
2985
FELIX
427
429
435L
435R
433
430
431
432
426
434
SK-N-FI
601L
601R
605L
605R
608L
608R
609L
609R
610L
610R
602L
602R
603L
603R
604L
604R
606L
606R
607L
607R
CHLA20
611L
611R
612L
612R
614L
614R
615L
615R
619L
619R
613L
613R
617L
617R
618L
618R
620L
620R

Sup Fig.12. Tumor measurements of cell line xenograft and patient derived xenograft models of NB treated with SHP099. Graph represents tumor volume of 7 individual cell line xenograft and patient derived xenograft models measured during SHP099 (75 mg/kg) treatment for 28 days. Related to Figure 6.

Sup Fig. 13

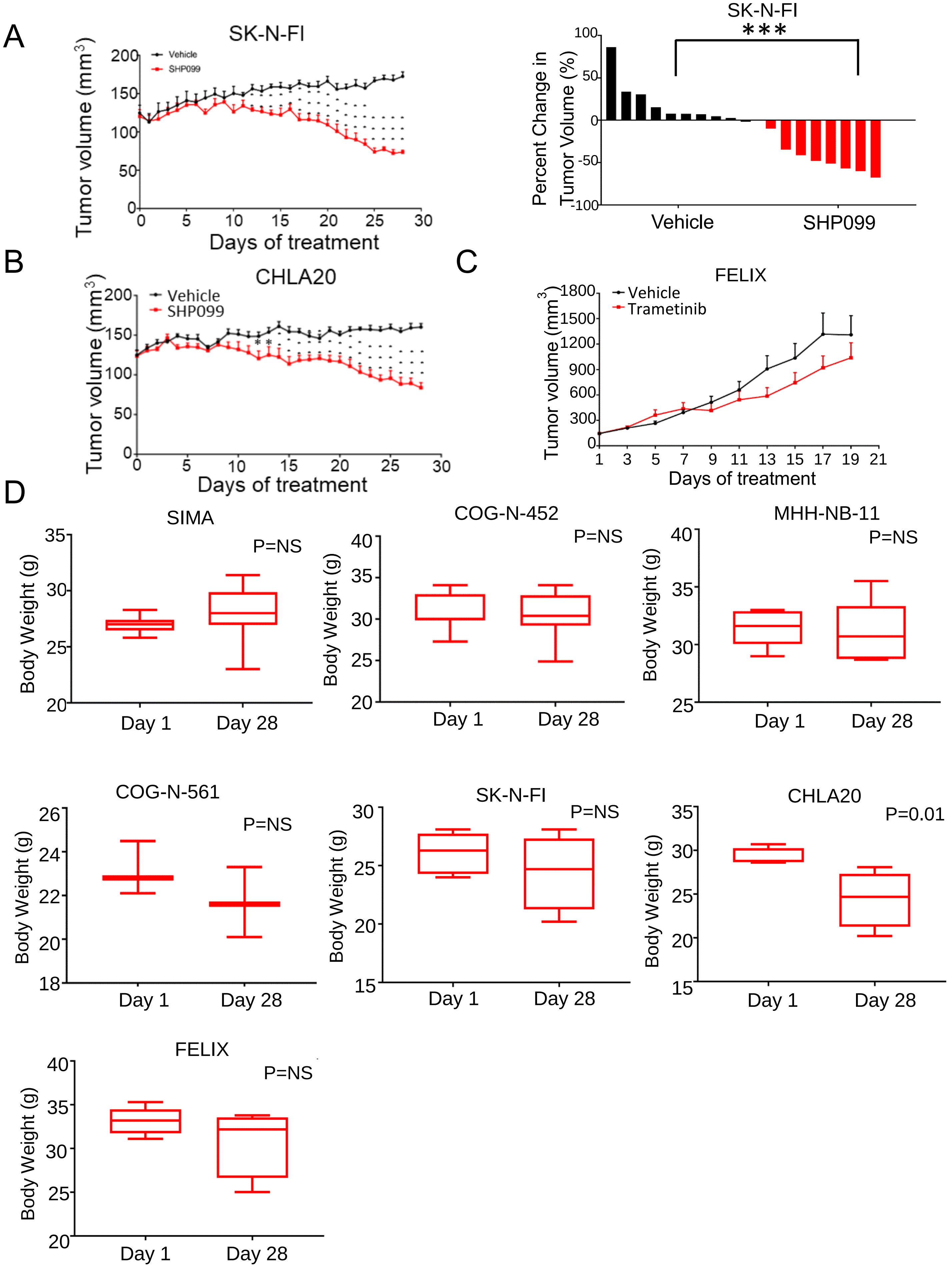
A
SK-N-FI
Tumor volume (mm3)
Vehicle
SHP099
Days of treatment
SK-N-FI
***
Percent Change in Tumor Volume (%)
Vehicle
SHP099
B
CHLA20
Tumor volume (mm3)
Vehicle
SHP099
Days of treatment
C
FELIX
Tumor volume (mm3)
Vehicle
Trametinib
Days of treatment
D
SIMA
P=NS
Body Weight (g)
Day 1
Day 28
COG-N-452
P=NS
Body Weight (g)
Day 1
Day 28
MHH-NB-11
P=NS
Body Weight (g)
Day 1
Day 28
COG-N-561
P=NS
Body Weight (g)
Day 1
Day 28
SK-N-FI
P=NS
Body Weight (g)
Day 1
Day 28
CHLA20
P=0.01
Body Weight (g)
Day 1
Day 28
FELIX
P=NS
Body Weight (g)
Day 1
Day 28

Sup Fig.13. SHP099 is effective *in vivo* and trametinib is ineffective *in vivo*. (A and B) Anti-tumor activity of SHP099 was assessed in mice bearing the indicated tumors. Mice were dosed with SHP099 (75 mg/kg) daily or vehicle (control) for 28 days. Waterfall plot represents change in tumor volume percent of each tumor to their initial tumor size (right panel) in the control and treated group. Error bars are ±SEM. Student t-test was used to calculate significance (*$p<0.05$, **$p<0.01$, ***$p<0.001$). C) FELIX PDX model treated with 1 mg/kg/qd trametinib. D) SHP099 treatment does not induce toxicity in mice. Body weight of SHP099 (75 mg/kg) treated *MYCN*-amplified or wild type xenograft and PDX mouse models of NB measured at day1 and day28 of treatment, NS= Non-significant by student's t test. Related to Figure 6.
