# Supplementary material for: High-risk neuroblastoma with NF1 loss of function is targetable using SHP2 inhibition
Source: Cell Rep. Author manuscript; Available in PMC 2023 Jul 19. (PMC10353975; doi:10.1016/j.celrep.2022.111095)
Supplement: 5 [file NIHMS1914136-supplement-5.pdf]

Cell Line Short Tandem Repeat (STR) Profile Report

Principal Investigator:
Institution:
Cell Line ID: SKNFI
Report Date: February 25, 2022
Submitting Researcher:
Sample Type Submitted: Cultured cells
Tracking ID: CLA-Mycoplasma Testing-Request_External - _2022.02.18
Profile Kit Requested: GenePrint 10 (Promega)

STR Graphic Profile:

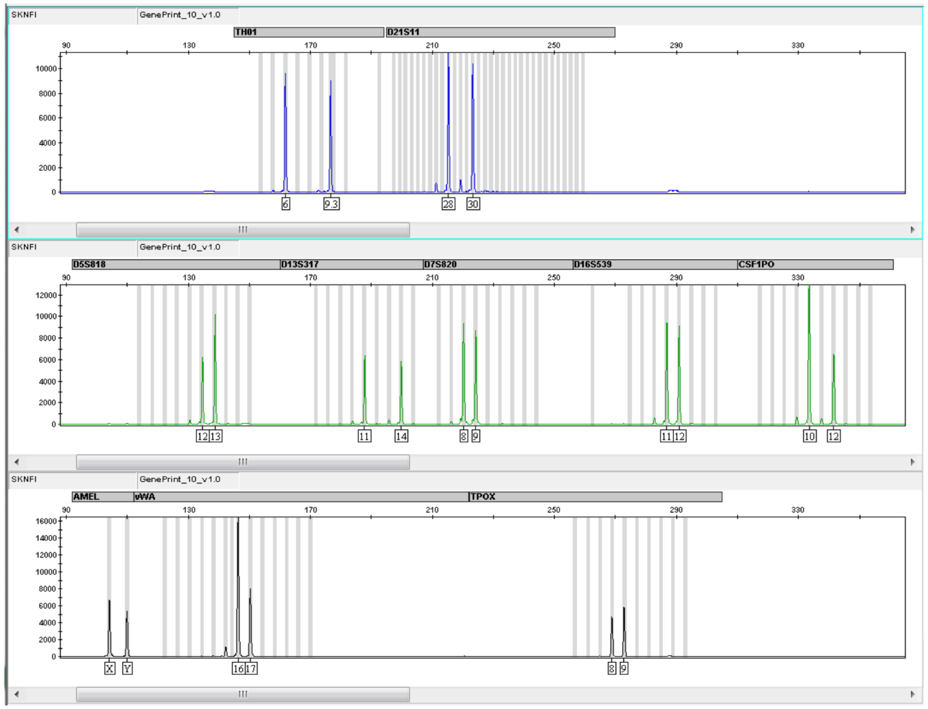

STR Profile:

Loci	SKNFI
AMEL	X,Y
CSF1PO	10,12
D13S317	11,14
D16S539	11,12
D21S11	28,30
D5S818	12,13
D7S820	8,9
TH01	6,9.3
TPOX	8,9
vWA	16,17

» Loci with one allele listed are homozygous for that STR marker.

Peaks that appear to be legitimate stutter or are spectral bleed-through artifacts are not labeled.

- This report provides a STR profile for the cell line submitted, it does not automatically authenticate the cell line. Authentication is determined by comparison to the STR profile of the original donor tissue or cell stock. This STR profile can be used to verify the cell line is human, evaluate profile consistency between provisionally related cell isolates, detect cross-contamination with another human cell line (intra-species contamination), and to compare to profile databases, which we strongly recommend. This STR report does not rule out cross-contamination with an inter-species cell line (non-human).
- Please see the Pdf file *Additional Information* sent with the results for more information about interpreting your STR profile and links to profile databases.

Principal Investigator:
Institution:
Cell Line ID: SKNFI
Report Date: February 25, 2022

Procedure:

A Promega GenePrint 10 Kit is used to PCR amplify eight short tandem repeat (STR) loci plus a gender determining marker, Amelogenin. The PCR product is electrophoresed on an ABI Prism® 3730xl Genetic Analyzer using an ILS600 internal lane standard (Promega). Data is analyzed using GeneMapper® v 4.0 software (Applied Biosystems). An allelic ladder that contains fragments of the same lengths as many of the known alleles for the loci is used to determine the amplified sample fragments alleles. Appropriate positive and negative controls are used.

Raw Data Table: (STR markers with one allele listed are homozygous for that marker.)

Sample Name	Marker	Allele 1	Allele 2	Size 1	Size 2	Height 1	Height 2	Peak Area 1	Peak Area 2
SKNFI	AMEL	X	Y	104.07	109.91	00:00.0	5360	40885	31766
SKNFI	CSF1PO	10	12	333.48	341.53	12944	6500	91378	46584
SKNFI	D13S317	11	14	187.77	199.74	6399	5825	39075	35907
SKNFI	D16S539	11	12	286.78	290.86	9432	9111	64526	61935
SKNFI	D21S11	28	30	215.17	223.21	11318	10398	70258	66048
SKNFI	D5S818	12	13	134.64	138.75	6209	10188	38279	61604
SKNFI	D7S820	8	9	220.21	224.23	9343	8684	59025	55886
SKNFI	TH01	6	9.3	161.83	176.65	9624	9063	57747	54285
SKNFI	TPOX	8	9	268.82	272.8	4669	5807	30814	38708
SKNFI	vWA	16	17	146.29	150.32	16485	8029	101342	50397

Cellosaurus

CLASTR 1.4.4

The Cellosaurus STR Similarity Search Tool

Markers

Human | Mouse | Dog

Marker	Value	Marker	Value
Amelogenin	X,Y	D1S1656	
CSF1PO	10,12	D2S441	
D2S1338		D6S1043	
D3S1358		D10S1248	
D5S818	12,13	D12S391	
D7S820	8,9	D22S1045	
D8S1179		DXS101	
D13S317	11,14	DYS391	
D16S539	11,12	F13A01	
D18S51		F13B	
D19S433		FESFPS	
D21S11	28,30	LPL	
FGA		Penta C	
Penta D		SE33	
Penta E			
TH01	6,9.3		
TPOX	8,9		
vWA	16,17		

Scoring

Algorithms:

- Tanabe
- Masters (vs. query)
- Masters (vs. reference)

Modes:

- Non-empty markers
- Query markers
- Reference markers

Include Amelogenin

Filters

Score Filter: 60%

Min Markers: 8

Max Results: 200

Actions

Search

Load File

Example

Reset

Help | About

Results of similarity search on the **7919 human** cell lines with STR profiles in Cellosaurus release **40.0**

Export table

Accession	Name	N° Markers	Score	Amel	CSF1PO	D2S1338	D3S1358	D5S818	D7S820	D8S1179	D13S317	D16S539	D18S51	D19S433	D21S11	FG
NA	Query	NA	NA	X,Y	10,12			12,13	8,9		11,14	11,12			28,30	
CVCL_1702	SK-N-FI	9	100.00%	X,Y	10,12	17,18	16,18	12,13	8,9	11,12	11,14	11,12	12,14	12	28,30	22
CVCL_0093 *Best*	RS4;11	9	76.47%	X	11,12	20,25	15,17	12,13	8,12	13,14	11	11,12	12,16	12,14	28,30	24,
CVCL_0093 *Worst*	RS4;11	9	72.73%	X	11,12	20,25	15,17	12	8,12	13,14	11	11,12	12,16	12,14	28,30	24,
CVCL_7811	UT-SCC-15	9	76.47%	X	12,15	19	15	12,13	8,9	13	11	11,12	12,14	15,15.2	28	25
CVCL_B886	GENEA078	9	75.00%	X	10,11	17,24	15	12,13	8,10	11,13	11	12	12,13	14,15	28,30	22,
CVCL_B451	OX-CO-1	9	75.00%	X	12			12	8,12		11	11,12			30	
CVCL_1982	CL-40	9	74.29%	X	10,12		16	11	8,9	12,14	8,11	11,12	14		30,32.2	21,
CVCL_YC25	GIBHi002-A	9	74.29%	X	10,12		15,18	12,13	8,11	10,14	8,12	11,12	13,16		28,30	22,
CVCL_0936	Hs 832(C).T	9	74.29%	X	10,12		14,16	10,11	8,9	12,13	11,12	11,12	16,17		28,32.2	24,
CVCL_0814	Hs 600.T	8	73.33%	X,Y	10,12			11,12	8,9		11	11,12				
CVCL_0860	Hs 704.Sk	8	73.33%	X	10,12			11,12	8,10		11,12	11,12				
CVCL_0861	Hs 704.T	8	73.33%	X	10,12			11,12	8,10		11,12	11,12				
CVCL_0887	Hs 742.Sk	8	73.33%	X	10,12			12	8,9		9,12	11,12				
CVCL_0888	Hs 742.T	8	73.33%	X	10,12			12	8,9		9,12	11,12				
CVCL_B160	HUES 29	9	72.73%	X	10,12		14,15	11,12	8,9	11,13	11,12	11,12	14,16		31.2	21,
CVCL_3901	EA.hy 926	8	72.73%	X	10,11,12			11	8,9,10		11	11,12				

CVCL_B165	HUES 33	9	72.22%	X	10,13	17,24	14,15	12,13	9,11	11	8,11	11,12	13,20	14	28,29	23,
CVCL_2197	SKW-3	9	72.22%	X,Y	10,12	19,25	15,18	12,13	8,12	11,14	8,12	11,12	13,18	13,15	28,29,30	24,
CVCL_A9W1	GSC#452C	8	71.43%	X	10,12			12,13	8		8,12	11				
CVCL_A9W2	GSC#452P	8	71.43%	X	10,12			12,13	8		8,12	11				
CVCL_4364	NCI-H1622	8	71.43%	X,Y	10			12	10,12		11	11,12				
CVCL_4W97	NCI-H23-Luc	8	71.43%	X	10			12,13	9,10		12	11				
CVCL_G002	HCC60	9	70.97%	X	10		15	12,13	9,10	15	12	11	14		30	24
CVCL_1458 *Best*	NCI-H1184	9	70.97%	X,Y	10		14	12	12	10,11	11	11,12	16,20		32.2	22
CVCL_1458 *Worst*	NCI-H1184	9	64.52%	X,Y	10		14	12	10,12	10,11	11	11,12	16,20		32.2	22
CVCL_1547 *Best*	NCI-H23	9	70.97%	X	10	18,23	15	12,13	9,10	15	12	11	14,16	12,14	30	24
CVCL_1547 *Worst*	NCI-H23	9	64.52%	X	10	18,23	15	12	9,10	15	12	9,11	14,16	12,14	30	24
CVCL_4613	BDCM	8	70.97%	X,Y	10,11			12,13	9,11		11	10,11				
CVCL_0F60	CCD-973Sk	8	70.97%	X	11,12			12,13	8,11		11,12	11,12				
CVCL_1327	KE-37	8	70.97%	X,Y	10,12			12,13	8,12		8,12	11,12				
CVCL_EE38	SIGi001-A	8	70.97%	X	10,12			12,13	8,11		8,12	11,12				
CVCL_2635 *Best*	NCI-BL1184	9	70.59%	X,Y	10		14,15	11,12	10,12	10,11	11	11,12	16,20		28,32.2	22,
CVCL_2635 *Worst*	NCI-BL1184	9	66.67%	X,Y	10		14,15	11,12	10,12	10,11	11	11,12	16,20		28,32.2	22,
CVCL_S426	NZM041	9	70.59%	X	10,11	19,20	15,16	12,13	10	13,15	8,11	11,12	12,19	13,14	29,30	25,
CVCL_S424	NZM047	9	70.59%	X	10,11	19,20	15,16	12,13	10	13,15	8,11	11,12	12,19	13,14	29,30	25,
CVCL_1635	Panc 03.27	9	70.59%	X	10,11		15,16	12,13	8,13	13,14	11,12	12	18		29,30	22,
CVCL_A5SM	Wilms11	9	70.59%	X,Y	10,12		15,17	12,13	8,9	13	12	11,12	14,21		29,30	23,
CVCL_S457	Fsp 62891	8	70.00%	X,Y	7,10,12,13			12	8,9,11,12		8,11,14	11,12,13				
CVCL_1158	D-392MG	8	68.97%	X	12,13			12	12		8,14	11,12				
CVCL_0F29	DFCI-LU011	8	68.97%	X,Y	10			12	9,12		11,12	11,12				
CVCL_AS83	HBEC30-KT	8	68.97%	X	10,11			11	8,9		11,12	11				
CVCL_0675	Hs 2.We	8	68.97%	X,Y	10,12			12,13	8,9		11	13				
CVCL_6404	HT-297.T	8	68.97%	X,Y	10,12			9,12	8,9		11,13	9,12				
CVCL_0871 *Best*	Hs 729.T	9	68.75%	X,Y	10		17	11,12	8,9	10,14	11	11	12		28,31.2	19,
CVCL_0871 *Worst*	Hs 729.T	9	66.67%	X,Y	10		17	11,12	8,9	10,14	11,12	11	12		28,31.2	19,
CVCL_IT36	NEM163	9	68.75%	X,Y	8,10			12,13	9		10,11	11			28,30	
CVCL_N707	BT054	8	68.75%	X	10,12			11,12	9,11		12,14	11,12				
CVCL_1058	A-204	9	68.57%	X	10,13	17,22	14,17	12	8,10	13,15	11,12	11,12	17,18	12,13	28,30	21
CVCL_8607 *Best*	92-1 [Human uveal melanoma]	9	66.67%	X	10,11		14,15	12,13	11	15	10,11	11,12	12,13		30	21,
CVCL_8607 *Worst*	92-1 [Human uveal melanoma]	9	48.48%	X	10,11		14,15	9,11	10,11	15	11,12	12	12,13		30	21,
CVCL_0202 *Best*	Caov-4	9	66.67%	X	10,11	17,20	16	11,13	8,10	12	12,14	9,11	21	11,12	28,33.2	22
CVCL_0202 *Worst*	Caov-4	9	64.71%	X	10	17,20	16	11	8,10	12	12,14	9,11	21	11,12	28,33.2	22
CVCL_2415	COR-L47	9	66.67%	X,Y	10,12		14	10,13	8,10	12,14	11	11,12	13,15		30,31.2	24
CVCL_9011	GENEA002	9	66.67%	X,Y	10,11	23	14,17	11,12	8,10	12,14	11	11,12	13,16	13,14	28,30	19,
CVCL_QX35	GIS2014-SB3C16	9	66.67%	X,Y	10,12	20	15,16	12	8,11	11,14	8	11	13	15,15.2	28,30	23,
CVCL_N127 *Best*	GM09948	9	66.67%	X,Y	10,11,12	23	15,17	11,13	11	12,13	11	11	15,18	13,14	29,30	24,
CVCL_N127 *Worst*	GM09948	9	62.50%	X,Y	10,11	23	15,17	11,13	11	12,13	11	11	15,18	13,14	29,30	24,
CVCL_VF64	HMGEC	9	66.67%	X,Y	10,12		15,17	10,13	8,11	13,15	9,14	8,11	12,16		29,30.2	23,
CVCL_B155	HUES 24	9	66.67%	X,Y	10,12	19,23	11,17	10,12	9	10,13	12,14	9,12	13,18	14,15	30	21,
CVCL_0D39	NZM075	9	66.67%	X,Y	11,12	20,24	15,16	11,12	8,9	16	8,11	11	17	14,15	28	20
CVCL_0103	SUP-B15	9	66.67%	X,Y	11,12		15,16	12,13	10,11	11,14	8,14	11,12	14		28,31	19,
CVCL_1738 *Best*	TCCSUP	9	66.67%	X	10			12	8,9	13	11,14	9,11	15		27,31.2	21
CVCL_1738 *Worst*	TCCSUP	9	62.50%	X	10			12	8,9	13	11	9,11	15		27,31.2	21

CVCL_7740	UM-SCC-3	9	66.67%	X	10,12		17	11,13	9,10	14	11,13	11,12	12,15		31.2	20,
CVCL_VV85	ACOSC3	8	66.67%	X,Y	10,12			12,13	9,10		8,11	11				
CVCL_2346	CCD-1097Sk	8	66.67%	X	12			10,12	8,10		11,12	11,12				
CVCL_3300	CCD-1106 KERTr	8	66.67%	X,Y	10			11,12	8,12		11,12	8,12				
CVCL_YB37	EDi045-A	8	66.67%	X,Y	10,12			12,13	8,10		9,12	11				
CVCL_C603 *Best*	FM74	8	66.67%	X,Y	11,12		15,16	12,13	8,9		8,9	12				20,
CVCL_C603 *Worst*	FM74	8	57.14%	X,Y	11,12		15,16	13	8,10		12	12				20,
CVCL_A9UJ	GSC#277	8	66.67%	X,Y	10,12			12	8,10		11	12,13				
CVCL_A9UQ	GSC#314C	8	66.67%	X	10			12,13	10,12		11,14	11				
CVCL_A9UR	GSC#314P	8	66.67%	X	10			12,13	10,12		11,14	11				
CVCL_J253	HCC1419-Luc	8	66.67%	X	10,12			10,12	11		9,12	11,12				
CVCL_0T97	HE48	8	66.67%	X	10,12			12,13	8,9		11	9,11				
CVCL_0809	Hs 58.Fs	8	66.67%	X,Y	10,12			11,12	9,10		10,11	11				
CVCL_1022	Hs 919.Sk	8	66.67%	X	10,12			12	10		11,12	11,13				
CVCL_1023	Hs 919.T	8	66.67%	X	10,12			12	10		11,12	11,13				
CVCL_5987	JHU-012	8	66.67%	X,Y	10			12,13	9,10		11	12				
CVCL_5991	JHU-022	8	66.67%	X,Y	10			12,13	9,10		11	12				
CVCL_5985	JHU-06	8	66.67%	X,Y	10			12,13	9,10		11	12				
CVCL_UR39	K-562-luc2	8	66.67%	X	9,10			11,12,13	9,11		8	11,12				
CVCL_5144	K562/MTX-2	8	66.67%	X	9,10			11,12	9,11		8	11,12				
CVCL_A119	Ma-Mel-06	8	66.67%	X,Y	10,12		14,15	12,13	10		11,13	12,13				22,
CVCL_1421	MN-60	8	66.67%	X,Y	11			12	10		11	11,12				
CVCL_V000	NCI-H2818	8	66.67%	X,Y	10,12			12,13	9,11		13	11,12				
CVCL_S410	PIG1	8	66.67%	X	10,12			12,13	8,10		8,11	8,11				
CVCL_2200	SPI-801	8	66.67%	X	10			12	9,11		8	11,12				
CVCL_2201	SPI-802	8	66.67%	X	10			12	9,11		8	11,12				
CVCL_1063	A-388	9	64.71%	X	10,11		13	11,13	10	12,13	11	11,12	14		28,30	22
CVCL_1090 *Best*	BONNA-12	9	64.71%	X	11,12		14,17	9,14	8,10	10,13	11,14	11,12	14		30,31.2	18,
CVCL_1090 *Worst*	BONNA-12	9	60.61%	X	11,12		14,17	9,14	8,10	10,13	11,14	12	14		30,31.2	18,
CVCL_DQ80	BSS1-PBMC-iPS 4F4	9	64.71%	X	10		14,15	11,12	9,10	11,16	8,11	11,12	13,14	13,14	30	19,
CVCL_1107 *Best*	CAL-27	9	64.71%	X	10,12	23,24	16	11,12	10	13,15	10,11	11,12	13	14,15.2	28,29	21,
CVCL_1107 *Worst*	CAL-27	9	64.71%	X	10,12	23,24	16	11,12	10	13,15	10,11	11,12	13	14,15.2	28,29	25
CVCL_1275	D425 Med	9	64.71%	X	9,12		14,17	12,13	11,12	11,15	11	11,12	15,17,18		29,33.2	20,
CVCL_YP34	H58	9	64.71%	X,Y	12			12	11,12		9,11	11,13			28,30	
CVCL_1251	HCC1419	9	64.71%	X	10,12		14,17	10,12	11	15	9,12	11,12	14		27,30	20,
CVCL_5135 *Best*	HCC461	9	64.71%	X	10,12		15,18	10,13	9,11	13,14	10,11	11,12	12,14		30	21,
CVCL_5135 *Worst*	HCC461	9	64.71%	X,Y	10,12		15,18	10,13	9,11	13,14	10,11	11,12	12,14		30	21,
CVCL_D605 *Best*	JJ012	9	64.71%	X	10	20,23	15,16,17,18	11,12	8,10	13	11	11,12	15	12,13	28,29	21,
CVCL_D605 *Worst*	JJ012	9	58.82%	X,Y	10	20,23	16,17	11,12	8,10	13	11	11,12	15,17	12,13	28,29	21,
CVCL_0004 *Best*	K-562	9	64.71%	X	9,10	17	16	11,12,13	9,11	12	8	11,12	15	14,14.2	29,30	21,
CVCL_0004 *Worst*	K-562	9	52.94%	X	9,10	17	16	11,12	9,11	12	8	11,12,13	15,16	14,14.2	29,31	21,
CVCL_WR77	LaNCE hiPSC-23	9	64.71%	X	12	19,20	15	12,13	11,12	10,15	11,14	8,11	15,18	13,15	28,29	23
CVCL_1380 *Best*	Loucy	9	64.71%	X	11,12	21,22	15,19	12	9,10	12,13	11,13	11	15,16	14	27,30	22
CVCL_1380 *Worst*	Loucy	9	62.86%	X	11,12	21,22	15,19	11,12	9,10	12,13	11,13	11	15,16	14	27,30	22
CVCL_RP45	LUTC-8	9	64.71%	X,Y	12,13	19,23	15	12,13	9,10	10,11	14	11,12	13,15	13,14	29,30	22
CVCL_A1WQ	RCNSi001-A	9	64.71%	X	10,12			12,13	8,11		11,14	13			28,31.2	
CVCL_A1WR	RCNSi001-B	9	64.71%	X	10,12			12,13	8,11		11,14	13			28,31.2	
CVCL_A1WS	RCNSi002-A	9	64.71%	X	10,12			12,13	8,11		11,14	13			28,31.2	

CVCL_A1WT	RCNSi002-B	9	64.71%	X	10,12			12,13	8,11		11,14	13			28,31.2	
CVCL_5593	SUM225CWN	9	64.71%	X	10,12		16,19	11,13	8,9	15	12	11	14		30,33.2	24
CVCL_4Z32	TC-215	9	64.71%	X,Y	11,12	16,26	14,17	12,13	8,10	12,16	12	12	14,16	14	28,34.2	19,
CVCL_W921	THJ-21T	9	64.71%	X,Y	11,15		15,17	12,13	8,10	9,14	11,12	11,12	13,15		30	24
CVCL_A1SS	TOV-3392D	9	64.71%	X	12			11,13	9,11		12,14	9,11			28,30	
CVCL_A5TC	CAISMOV24	9	64.52%		12	17	16,17	12,13	8,12	10,13	11,12	12	12,19	13,14	30,32.2	23,
CVCL_2059 *Best*	HCC366	9	64.52%	X	10,12		15,17	12	10,11	15	11,13	11	19		28	19,
CVCL_2059 *Worst*	HCC366	9	60.00%	X	10,12		15,17	12	10,11	15	13	11	19		28	19,
CVCL_V579 *Best*	HCC4017	9	64.52%	X	10,11		16	11	8,9	13,14	12	11	16		28,30	20
CVCL_V579 *Worst*	HCC4017	9	62.50%	X	10,11		16	11	8,9	13,14	12	11	16		28,30	20
CVCL_0031 *Best*	MCF-7	9	64.52%	X	10	21,23	16	12	8,9	10,14	11	11,12	14	13,14	30	23,
CVCL_0031 *Worst*	MCF-7	9	62.50%	X	10	21,23	16	11,12	8,9	10,14	11	11,12	14	13,14	30	23,
CVCL_0U80	MCF-7/HER2-18	9	64.52%	X	10		16	12	8,9	10,14	11	11,12	14		30	23,
CVCL_M436	MCF-7/TAMR-1	9	64.52%	X	10		16	12	8,9	10,14	11	11,12	14		30	23,
CVCL_S429	NZM039	9	64.52%	X,Y	10	19,20	14	12	8,10	13,15	11,14	10,12	13,19	14,16	30	21,
CVCL_6183	SK-RC-26A	9	64.52%	X	11,12	19	16	13	8,9	14	11	11	13,14	15.2	28,30	20
CVCL_3120	SK-RC-26B	9	64.52%	X	11,12	19	16	13	8,9	14	11	11	13	15.2	28,30	20
CVCL_2745	UM-UC-11	9	64.52%	X,Y	10		15,16	12	8,12	11,13	11,14	11	13		30	20,
CVCL_1047	380	8	64.52%	X,Y	10,11			11,13	9,11		11,12	11,12				
CVCL_2394	CCD-39Lu	8	64.52%	X	10,12			11,12	9,12		11	11,13				
CVCL_2395	CCD-39Sk	8	64.52%	X	10,12			11,12	9,12		11	11,13				
CVCL_A9UD	GSC#221	8	64.52%	X,Y	10			11,13	8,13		8,11	11,12				
CVCL_A9UT	GSC#318	8	64.52%	X,Y	10,12			12	9,10		11,12	11,12				
CVCL_D095	HES-5	8	64.52%	X,Y	10,11			10,12	9,13		11	11,12				
CVCL_0833	Hs 67	8	64.52%	X	11,12			12	8,12		11,12	11,13				
CVCL_R709	Hs 677.Rec	8	64.52%	X	11,12			12	9,11		13,14	11,12				
CVCL_0837	Hs 677.Sk	8	64.52%	X	11,12			12	9,11		13,14	11,12				
CVCL_0838	Hs 677.Tg	8	64.52%	X	11,12			12	9,11		13,14	11,12				
CVCL_0F27	HUVEC-CS	8	64.52%	X	11,12			11,12	8,12		9,11	11,12				
CVCL_6413	KMA	8	64.52%	X,Y	10,12			12,13	11,12		11,12	12,13				
CVCL_6416	LTR228	8	64.52%	X,Y	11,12			12,13	9,12		11	11,12				
CVCL_L224	OKF6/TERT-1	8	64.52%	X	11,12			12,13	8,10		11	11,12				
CVCL_L225	OKF6/TERT-2	8	64.52%	X,Y	11,12			12,13	8,10		11	11,12				
CVCL_B347	SA001	8	64.52%	X,Y	10,13			12,13	8,10		11,12	12,13				
CVCL_3796	SKW 6.4	8	64.52%	X,Y	11,12			12,13	9,11		11	11,12				
CVCL_1722 *Best*	SW1783	8	64.52%	X,Y	10,13			12,13	9,13		9,11	12				
CVCL_1722 *Worst*	SW1783	8	60.00%	X,Y	10,13			12	9,13		9,11	12				
CVCL_AR68	TK6 (IVGT)	8	64.52%	X,Y	11,12			12,13	9,11		11	11,12				
CVCL_2733	TK6TGR	8	64.52%	X,Y	11,12			12,13	9,11		11	11,12				
CVCL_6741	WI-L2-729HF2	8	64.52%	X,Y	11,12			12,13	9,11		11	11,12				
CVCL_2761 *Best*	WIL2 NS	8	64.52%	X,Y	11,12			12,13	9,12		11	11,12				
CVCL_2761 *Worst*	WIL2 NS	8	58.06%	X,Y	11,12			12,13	9,12		11	11,12				
CVCL_3809	WIL2 S	8	64.52%	X,Y	11,12			12,13	9,11		11	11,12				
CVCL_RZ81	XYFMGG23G	8	64.52%	X,Y	10,11			11,12	8,9		11,12	11				
CVCL_0165	BCBL-1	8	64.29%	X	12			11,13	8,9		11,12	12				
CVCL_2382	CCD-19Lu	8	64.29%	X	10,12			12	9,11		11,12	11				
CVCL_C617 *Best*	FM93/2	8	64.29%	X	12,14		18	11,12	9		11	11,12				20
CVCL_C617 *Worst*	FM93/2	8	62.07%	X	12,14		18	11,12	9		11	11,12				20
CVCL_G356	HT-29-MTX-E12	8	64.29%	X	11,12			11,12	10		11	11,12				

CVCL_A919	chHES-183	9	62.86%	X,Y	10,12		16,18	10,11	8,9	12,16	11,14	12	16		28,29	16,
CVCL_6594	CHLA-15	9	62.86%	X	10,12	17,26	16	12,15	9,11	13	10,11	11,13	15,19	14,15	30,31.2	23,
CVCL_2410	COR-L26	9	62.86%	X	12,13		18	11,13	9,11	11,14	11	11,13	15		28,30	18,
CVCL_QX37	GIS2014-WT3C16	9	62.86%	X,Y	10,12	20	16	10,12	8,11	11,14	9,10	11,12	13	15,15.2	28,30	18,
CVCL_2521 *Best*	HT29/219	9	62.86%	X	11,12		15,17	11,12	10	10	11,12	11,12	13		28,30	19,
CVCL_2521 *Worst*	HT29/219	9	60.61%	X	11		15,17	11,12	10	10	11	11,12	13		28,30	19,
CVCL_2959	HUVEC-C	9	62.86%	X	11,12		16	11,12	8,12	14,16	9,11	11,12	13,17		28,31	21,
CVCL_X151	JFEN	9	62.86%	X,Y	12,13	20,24	16,18	11,12	8,10	11,14	11,12	9,12	12,18	14,15	28,30	20,
CVCL_VE24	KITi001-A	9	62.86%	X,Y	12,13	19,23	15,17	12,13	9,12	12,14	11	11,12	14	14,16.2	30,31	22,
CVCL_VE25	KITi001-A-35	9	62.86%	X,Y	12,13	19,23	15,17	12,13	9,12	12,14	11	11,12	14	14,16.2	30,31	22,
CVCL_WS27	LN-2207GS	9	62.86%	X,Y	10,11,12	19,25	14,16	11,12	8	11,13	11,12	12	11,14	13,14	30,31	22,
CVCL_WV16	SJG-31	9	62.86%	X	10,12		16	10,11	9,12	12,14	13,14	10,11	13,18		30	21,
CVCL_6169	SK-RC-2	9	62.86%	X,Y	11,12	16,24	17	12	9,11	8,15	9,11	11,12	12,19	12,13	28,30	20,
CVCL_VE35	TUSMi003-A	9	62.86%	X	10,12	20	17	12,13	8,9	10	8,9	11,12	17,19	12,13.2	29,30	24
CVCL_S471 *Best*	U-343MG	9	62.86%	X,Y	10,12	22,24	15,17	12,13	9,11	13,14	9,13	9,12	23	13,14	31,33.2	19,
CVCL_S471 *Worst*	U-343MG	9	61.11%	X,Y	10,12	22,24	15,17	12,13	9,11	13,14	9,13	9,12	21,23	13,14	31,33.2	19,
CVCL_RV27	ULUNDi005-A	9	62.86%	X,Y	11,12			12	8,11		11,13	9,11			28,33.2	
CVCL_0571	UROtsa	9	62.86%	X	11,12		15	12,13	11,12	14	11,13	11,12	16		27,29	20
CVCL_A5SC	Wilms1-2I	9	62.86%	X	10,12		14,17	12,13	9,14	12,14	11,13	11,14	15,18		30,31	22,
CVCL_0144	ARO	9	62.50%	X	11,12		15,17	11,12	10	10,16	11	11,12	12,13		30	20,
CVCL_8207	GLC-2	9	62.50%	X,Y	12,13		17	12,13	9,12	12	14	12	15,18		30,32	21,
CVCL_1269 *Best*	HCC4006	9	62.50%	X	10		18	12	9,12	10,14	11,12	11,12	19		31	21,
CVCL_1269 *Worst*	HCC4006	9	58.06%	X	10		18	12	9,12	10,14	11,12	12	19		31	21,
CVCL_D224	HNO41	9	62.50%	X	10	27	17	10,13	8	13	12	11,12	12	14	30	22
CVCL_EG32	JVE774	9	62.50%	X,Y	10,11			12	8		11,12	9,12			28,29	
CVCL_2094 *Best*	KPL-1	9	62.50%	X	10			11,12	8,9		11	11,12			30	
CVCL_2094 *Worst*	KPL-1	9	60.61%	X	10			11,12	8,9		10,11	11,12			30	
CVCL_UC24	LM-MEL-9	9	62.50%	X,Y	11			13	9,11		11	11,12			29,30	
CVCL_1D49	MCF-7/164R-4	9	62.50%	X	10		16	11,12	8,9	10,14	11	11,12	14		30	23,
CVCL_1D39	MCF-7/164R-7	9	62.50%	X	10		16	11,12	8,9	10,14	11	11,12	14		30	23,
CVCL_1D42	MCF-7/TAMR-4	9	62.50%	X	10		16	11,12	8,9	10,14	11	11,12	14		30	23,
CVCL_1D43	MCF-7/TAMR-7	9	62.50%	X	10		16	11,12	8,9	10,14	11	11,12	14		30	23,
CVCL_6860	MCF-7B	9	62.50%	X	10	21,23	16	11,12	8,9	10,14	11	11,12	14	13,14	30	23,
CVCL_XD68	MCF7-Cas9-542	9	62.50%	X	10	21,23	16	11,12	8,9	10,14	11	11,12	14	13,14	30	23,
CVCL_XD69	MCF7-Cas9-543	9	62.50%	X	10	21,23	16	11,12	8,9	10,14	11	11,12	14	13,14	30	23,
CVCL_XD70	MCF7-Cas9-544	9	62.50%	X	10	21,23	16	11,12	8,9	10,14	11	11,12	14	13,14	30	23,
CVCL_0426 *Best*	MG-63	9	62.50%	X,Y	10,12	17,24	15	11,12	10	13	11	11,12	16	13,14	30	21,
CVCL_0426 *Worst*	MG-63	9	58.06%	X,Y	10,12	17,24	15	11,12	10	13	11	11	16	13,14	30	21,
CVCL_2638	NCI-BL1395	9	62.50%	X	12	17,24	15,18	12	8	12,14	10,14	11,13	12,14	13,16	28,29	18,
CVCL_1507	NCI-H1930	9	62.50%	X	12		16	13	9,10	12,14	8,11	11	12,19		30	21
CVCL_0D37	NZM073	9	62.50%	X	12	19,20	16	12	8,12	10,13	12,13	12	12,18	14,15	30	21
CVCL_WV09	SJG-8	9	62.50%	X,Y	11,12		15,17	9,12	9	10,13	11	10,11	12,16		28,30	21
CVCL_E998	WaGa	9	62.50%	X,Y	12		14	12	10	10,13	8,13	11,12	10,13		28,30	19
CVCL_2350	CCD-1100Sk	8	62.50%	X	10,11			12,13	7,9		9,11	9,11				
CVCL_2357	CCD-1118Sk	8	62.50%	X	11,12			12,13	9,10		11,12	11,13				
CVCL_X503	U-2946	8	62.50%	X,Y	10,11			11,12	8,10		11,12	11,12				
CVCL_1500	NCI-H1869	9	62.07%	X	10		17	11	8,9	13,14	11	12	12,18		31	22
CVCL_V610	SLR24	9	62.07%	X	10,12			12	8		11,12	11			30	
CVCL_2330	CCD-1068Sk	8	62.07%	X	9			12,13	8,9		8,11	11,12				

CVCL_2338	CCD-1086Sk	8	62.07%	X	10,12			11,13	9,12		12	11,12				
CVCL_4150	Cri du Chat	8	62.07%	X	10,12			10,11	8,11		11	11,12				
CVCL_A9T7	GSC#1	8	62.07%	X	10			12,13	8,10		8	11,14				
CVCL_A9V5	GSC#389	8	62.07%	X,Y	10,11			12	9,10		8,11	11,12				
CVCL_A9VF	GSC#406	8	62.07%	X,Y	10			12,13	10,12		14	9,12				
CVCL_0T89	HE36	8	62.07%	X,Y	10			12,13	8,12		11,12	9				
CVCL_2941	HFb16d	8	62.07%	X,Y	10,12			10,13	9,10		11	12,13				
CVCL_3290	HIAE-65	8	62.07%	X	10,11			12	9,12		11,14	11,13				
CVCL_3294	HIVE-65	8	62.07%	X	10,11			12	9,12		11,14	11,13				
CVCL_0720	Hs 3.T	8	62.07%	X	10,11			11,12	9		11	11,12				
CVCL_0802	Hs 571.T	8	62.07%	X	10			11	8,9		10,11	11,12				
CVCL_R717	Hs 680.Li	8	62.07%	X	11,12			12	9,10		11,14	9,11				
CVCL_0840	Hs 680.Rec	8	62.07%	X	11,12			12	9,10		11,14	9,11				
CVCL_0841	Hs 680.Sk	8	62.07%	X	11,12			12	9,10		11,14	9,11				
CVCL_0842	Hs 680.Tg	8	62.07%	X	11,12			12	9,10		11,14	9,11				
CVCL_0843	Hs 680.Tr	8	62.07%	X	11,12			12	9,10		11,14	9,11				
CVCL_JM00	K-562-GFP	8	62.07%	X	9,10			11,12	9,11		8	11,12				
CVCL_J257	K562-Luc	8	62.07%	X	9,10			11,12	9,11		8	11,12				

SIB Swiss Institute of Bioinformatics | Disclaimer

Article: Robin T, Capes-Davis A, Bairo

Cell Line Short Tandem Repeat (STR) Profile Report

Principal Investigator:
Institution:
Cell Line ID: CHLA20
Report Date: February 25, 2022
Submitting Researcher:
Sample Type Submitted: Cultured cells
Tracking ID: CLA-Mycoplasma Testing-Request_External - _2022.02.18
Profile Kit Requested: GenePrint 10 (Promega)

STR Profile:

Loci	CHLA20
AMEL	X
CSF1PO	10,12
D13S317	11
D16S539	13
D21S11	30,31.2
D5S818	12,15
D7S820	9,11
TH01	6,7
TPOX	8
vWA	17

» Loci with one allele listed are homozygous for that STR marker.

STR Graphic Profile:

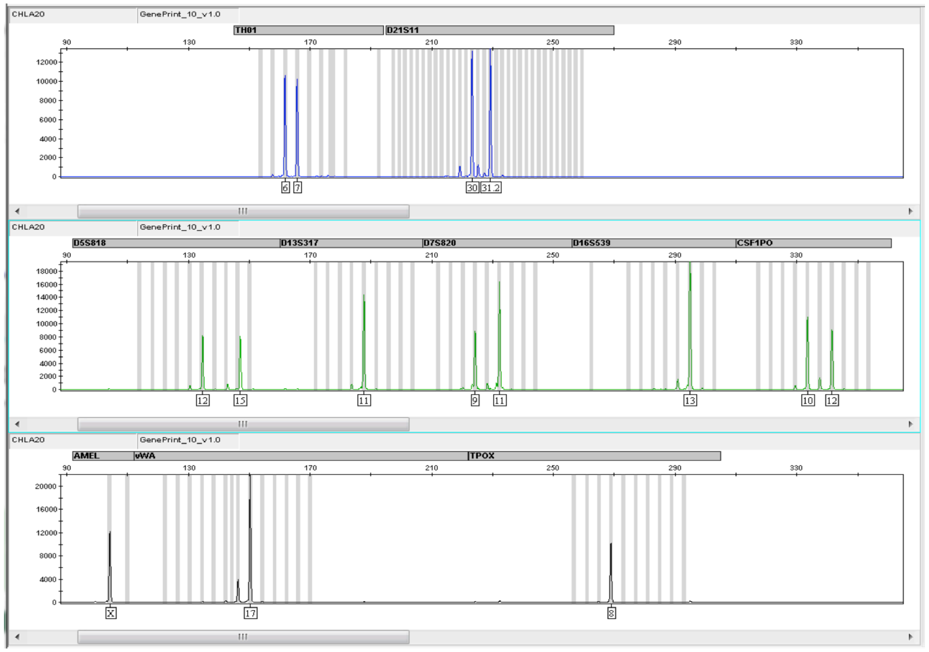

Peaks that appear to be legitimate stutter or are spectral bleed-through artifacts are not labeled.

- This report provides a STR profile for the cell line submitted, it does not automatically authenticate the cell line. Authentication is determined by comparison to the STR profile of the original donor tissue or cell stock. This STR profile can be used to verify the cell line is human, evaluate profile consistency between provisionally related cell isolates, detect cross-contamination with another human cell line (intra-species contamination), and to compare to profile databases, which we strongly recommend. This STR report does not rule out cross-contamination with an inter-species cell line (non-human).
- Please see the Pdf file *Additional Information* sent with the results for more information about interpreting your STR profile and links to profile databases.

Principal Investigator:
Institution:
Cell Line ID: CHLA20
Report Date: February 25, 2022

Procedure:

A Promega GenePrint 10 Kit is used to PCR amplify eight short tandem repeat (STR) loci plus a gender determining marker, Amelogenin. The PCR product is electrophoresed on an ABI Prism® 3730xl Genetic Analyzer using an ILS600 internal lane standard (Promega). Data is analyzed using GeneMapper® v 4.0 software (Applied Biosystems). An allelic ladder that contains fragments of the same lengths as many of the known alleles for the loci is used to determine the amplified sample fragments alleles. Appropriate positive and negative controls are used.

Raw Data Table: (STR markers with one allele listed are homozygous for that marker.)

Sample Name	Marker	Allele 1	Allele 2	Size 1	Size 2	Height 1	Height 2	Peak Area 1	Peak Area 2
CHLA20	AMEL	X		104.17		12207		72574	
CHLA20	CSF1PO	10	12	333.48	341.53	11012	9130	77411	64716
CHLA20	D13S317	11		187.69		14404		86654	
CHLA20	D16S539	13		294.85		19420		132940	
CHLA20	D21S11	30	31.2	223.2	229.2	13174	13433	81685	83137
CHLA20	D5S818	12	15	134.65	147.06	8206	8112	49896	48411
CHLA20	D7S820	9	11	224.23	232.29	8833	16347	56136	105509
CHLA20	TH01	6	7	161.75	165.68	10653	10286	63324	61565
CHLA20	TPOX	8		268.89		10265		67906	
CHLA20	vWA	17		150.27		22040		134661	

Cellosaurus

CLASTR 1.4.4

The Cellosaurus STR Similarity Search Tool

Markers

Human | Mouse | Dog

Marker	Value	Marker	Value
Amelogenin	X	D1S1656	
CSF1PO	10,12	D2S441	
D2S1338		D6S1043	
D3S1358		D10S1248	
D5S818	12,15	D12S391	
D7S820	9,11	D22S1045	
D8S1179		DXS101	
D13S317	11	DYS391	
D16S539	13	F13A01	
D18S51		F13B	
D19S433		FESFPS	
D21S11	30,31.2	LPL	
FGA		Penta C	
Penta D		SE33	
Penta E			
TH01	6		
TPOX	8		
vWA	17		

Scoring

Algorithms:

- Tanabe
- Masters (vs. query)
- Masters (vs. reference)

Modes:

- Non-empty markers
- Query markers
- Reference markers

Include Amelogenin

Filters

Score Filter: 60%

Min Markers: 8

Max Results: 200

Actions

Search

Load File

Example

Reset

Help | About

Results of similarity search on the **7919 human** cell lines with STR profiles in Cellosaurus release **40.0**

Export table

Accession	Name	Nº Markers	Score	Amel	CSF1PO	D2S1338	D3S1358	D5S818	D7S820	D8S1179	D13S317	D16S539	D18S51	D19S433	D21S11	FG
**NA**	**Query**	**NA**	**NA**	**X**	**10,12**			**12,15**	**9,11**		**11**	**13**			**30,31.2**	
CVCL_6602	CHLA-20	9	92.86%	X	10,12	17	16	12,15	9,11	13	11	13	15,19	14,15	30,31.2	23,
CVCL_AQ27	CHLA-255	9	92.86%	X	10,12	17	16	12,15	9,11	13	11	13	15,19	14,15	30,31.2	23,
CVCL_6594	CHLA-15	9	86.67%	X	10,12	17,26	16	12,15	9,11	13	10,11	11,13	15,19	14,15	30,31.2	23,
CVCL_II76	HMC3	8	72.00%	X	10,11			11,12	9,11		11	12,13				
CVCL_Y546	I83-LCL	8	72.00%	X,Y	10,11			11,12	9,11		11,13	13				
CVCL_2382	CCD-19Lu	8	69.57%	X	10,12			12	9,11		11,12	11				
CVCL_1375 *Best*	LCLC-103H	9	69.23%	X	10,12	16,19	16,17	12	8,11	12,14	11	13	19	15	29,31.2	22
CVCL_1375 *Worst*	LCLC-103H	9	66.67%	X,Y	10,12	16,19	16,17	11,12	8,11	12,14	11	13	19	15	29,31.2	22
CVCL_2394	CCD-39Lu	8	69.23%	X	10,12			11,12	9,12		11	11,13				
CVCL_2395	CCD-39Sk	8	69.23%	X	10,12			11,12	9,12		11	11,13				
CVCL_6413	KMA	8	69.23%	X,Y	10,12			12,13	11,12		11,12	12,13				
CVCL_RZ85	XYFMGC50H	8	69.23%	X,Y	10,12			12	9,11		11,14	10,13				
CVCL_RZ79	XYFMGG18B	8	69.23%	X,Y	10,12			12,13	9		11,13	9,13				
CVCL_1069	ALL-PO	9	66.67%	X	10,11			11,12	9,11		11,12	13			29,30	
CVCL_A9SP	NP4	9	66.67%	X,Y	10,12	17,25	16,17	11,12	10,11	13	11,12	11,13	15,16	14,14.2	30	19,
CVCL_S417	NZM061	9	66.67%	X,Y	11,12	16,18	17	11,12	11	12,13	11,13	13	12,13	14,15	29,30	19,

CVCL_VP49	BCJ-4T	8	66.67%	X,Y	10,12		16,18	12	11	11,14	11,12	12	12,17			23,
CVCL_6404	HT-297.T	8	66.67%	X,Y	10,12			9,12	8,9		11,13	9,12				
CVCL_8504	KYSE-1440	8	66.67%	X	10			12	9,11		11	9,13				
CVCL_B279	S2-007	8	66.67%	X	11,12			11,12	9,11		9,11	9				
CVCL_F972	S2-VP10	8	66.67%	X	11,12			11,12	9,11		9,11	9				
CVCL_5728	CLS-54	9	64.29%	X	12		18	13	10,11	11	11	12,13	11,17,18		30,31.2	20
CVCL_B160	HUES 29	9	64.29%	X	10,12		14,15	11,12	8,9	11,13	11,12	11,12	14,16		31.2	21,
CVCL_2614	MOG-G-UVW	9	64.29%	X,Y	10,11	21,24	15	11,12	9,12	13,15	11	13	14,15	14	30,30.2	23,
CVCL_2901	FLAM-76	8	64.00%	X,Y	10,12			9,12	9,10		8,11	9				
CVCL_A9UJ	GSC#277	8	64.00%	X,Y	10,12			12	8,10		11	12,13				
CVCL_A9VM	GSC#421	8	64.00%	X,Y	10,12			10,12	9,11		11	10,12				
CVCL_A9W0	GSC#450	8	64.00%	X	10			11,12	9,11		11,13	11,13				
CVCL_A9TH	GSC#68	8	64.00%	X,Y	10			11,12	10,11		10,11	11,13				
CVCL_0809	Hs 58.Fs	8	64.00%	X,Y	10,12			11,12	9,10		10,11	11				
CVCL_1022	Hs 919.Sk	8	64.00%	X	10,12			12	10		11,12	11,13				
CVCL_1023	Hs 919.T	8	64.00%	X	10,12			12	10		11,12	11,13				
CVCL_Y437	I83-E95	8	64.00%	X,Y	10,11			11,12	9.3,11		11,13	13				
CVCL_QW85	MDA-T120	8	64.00%	X	12,14			11,12	10,11		11	13				
CVCL_1478	NCI-H1573	8	64.00%	X	10,12			11,13	9,11		11,13	12				
CVCL_B280	S2-013	8	64.00%	X	11,12			11,12	9,11		9,11	9				
CVCL_B281	S2-020	8	64.00%	X	11,12			11,12	9,11		9,11	9				
CVCL_F971	S2-CP8	8	64.00%	X	11,12			11,12	9,11		9,11	9				
CVCL_1698 *Best*	SK-MG-1	8	64.00%	X	12,15			11,12	9,11		11	12,13				
CVCL_1698 *Worst*	SK-MG-1	8	58.33%	X	12,15			11,12	9		11	12,13				
CVCL_YU16	NCC-MPNST4-C 1	9	63.64%	X,Y	12			12	11		10	9			30	
CVCL_3061	NCR-G3	8	63.64%	X,Y	9,12			12	11,13		11	13				
CVCL_0293 *Best*	HEC-1-A	9	62.50%	X	10,12	18,19	15	11,14,15	9,11	13,14	11,12	12,13	16,21	13	30,31	21,
CVCL_0293 *Worst*	HEC-1-A	9	54.55%	X	10,13,14	18,19	15	11,15	9,11	13,14	11	12,13	16,21	13	30,31	21,
CVCL_9S76	GENYOi006-A	9	62.07%	X,Y	10,12	21,22	14,15	10,12	9,11	15	11	9,15	14,16	12,14	27,30	20,
CVCL_YN83	GENYOi006-A-1	9	62.07%	X,Y	10,12	21,22	14,15	10,12	9,11	15	11	9,14	14,16	12,14	27,30	20,
CVCL_YN84	GENYOi006-A-2	9	62.07%	X,Y	10,12	21,22	14,15	10,12	9,11	15	11	9,14	14,16	12,14	27,30	20,
CVCL_YP34	H58	9	62.07%	X,Y	12			12	11,12		9,11	11,13			28,30	
CVCL_VR76	ICCSICi008-A	9	62.07%	X,Y	10,12			11,12	8,11		9,11	12,13			30	
CVCL_EG16	JVE015	9	62.07%	X	12			11,12	9,11		11,12	9			30,32.2	
CVCL_2993	KMS-27	9	62.07%	X,Y	10,12		16,17	10,12	11	11,12	10,11	11,12	14,16		30,32.2	22,
CVCL_S432	NZM027	9	62.07%	X	11	17,23	17,20	11,12	9,11	11,12	11,13	12	12,14	12	30,31.2	22,
CVCL_2675	OV7	9	62.07%	X	12,13	20,24	16	11,12	9,11	10,14	8,11	13	12,14	14	30,31.2	23
CVCL_A1WQ	RCNSi001-A	9	62.07%	X	10,12			12,13	8,11		11,14	13			28,31.2	
CVCL_A1WR	RCNSi001-B	9	62.07%	X	10,12			12,13	8,11		11,14	13			28,31.2	
CVCL_A1WS	RCNSi002-A	9	62.07%	X	10,12			12,13	8,11		11,14	13			28,31.2	
CVCL_A1WT	RCNSi002-B	9	62.07%	X	10,12			12,13	8,11		11,14	13			28,31.2	
CVCL_1706 *Best*	SNB-75	9	62.07%	X	10,12	17,23	17	12	9,11	12	8,12	11,13	18	13,14	30	19,
CVCL_1706 *Worst*	SNB-75	9	51.85%	X	10,12	17,23	17	12	9	12	8,12	11,13	18	13,14	30	19,
CVCL_3172	SUIT-2	9	62.07%	X	11,12		16	11,12	9,11	14,15	9,11	9	16		29,30	20,
CVCL_1G08 *Best*	HROC80 T1 M1	9	61.54%	X	12			12,13	9,11		8	10			30,30.2	
CVCL_1G08 *Worst*	HROC80 T1 M1	9	59.26%	X	12			12,13	9,11		8	10,15			30,30.2	
CVCL_A4GK	hTERT-VFF E10	9	61.54%	X	10,11		14,18	11,12	11	10,13	13	11,13	15,19		[illegible]31.2	22,
CVCL_A4GI	hTERT-VFF E6	9	61.54%	X	10,11		14,18	11,12	11	10,13	13	11,13	15,19		29,31.2	22,

CVCL_A4GJ	hTERT-VFF E7	9	61.54%	X	10,11		14,18	11,12	11	10,13	13	11,13	15,19		29,31.2	22,
CVCL_A625 *Best*	IPH-926	9	61.54%	X	10,12	19,20	15	11	9,11	13,15	11	12	12,22	16	30	19
CVCL_A625 *Worst*	IPH-926	9	53.85%	X	10,12	19,20	15	11	9,11	13,15	11	12	12,22	16	30	19
CVCL_RP46	LUTC-10	9	61.54%	X,Y	11,12	17,25	14,16	11	9,11	13,14	11,14	9,11	14	14	30	19,
CVCL_0485 *Best*	PLC/PRF/5	9	61.54%	X	10	19	15	12	9,11	13,16	11,12	13	17	11,13	30,33.2	19
CVCL_0485 *Worst*	PLC/PRF/5	9	59.26%	X	10	19,22	15	12	9,11	13,16	11,12	13	17	11,13	30,33.2	19
CVCL_0167 *Best*	SK-N-BE(2)-M17	9	61.54%	X	10	17,23	19	12	9,10	13,14	11	11	14	12,13	30,32.2	22,
CVCL_0167 *Worst*	SK-N-BE(2)-M17	9	48.00%	X	10	23	19	12	9,10	13,14	11	9,11	16,26	12,13	30,32.2	22,
CVCL_IR61	U3013MG	9	61.54%	X	10,12	25,26	17,19	12	9,11	13,14	14	10	13,14	14	31.2,32.2	18,
CVCL_C279	WM3918	9	61.54%	X,Y	12	19,24	17	11,12	12	13,16	11	10,13	9,13	14,16	28,30	20,
CVCL_6364	9D10	8	61.54%	X,Y	10,12			11,12	8,11		9,13	11,13				
CVCL_YB33	EDi041-A	8	61.54%	X	10,12			11,12	11,12		11,12	12,13				
CVCL_2490	HG-261	8	61.54%	X,Y	11,12			11,12	10,11		9,11	12,13				
CVCL_3519	Hs 207.T	8	61.54%	X	10,12			12	11,12		11,12	11,12				
CVCL_0695	Hs 222.Sp	8	61.54%	X	10,12			11,12	9,11		9,11	12,13				
CVCL_0747	Hs 401.Lu	8	61.54%	X	10,12			11,13	9,11		11,12	8,9				
CVCL_0859	Hs 701.T	8	61.54%	X	10,12			10,12	11,12		11,12	11				
CVCL_9Q53	HUVEC/TERT2	8	61.54%	X	12			15,16	9,11		11,12	9,13				
CVCL_UJ14	JMP-1	8	61.54%	X,Y	11,12			11,12	11,12		11	9,13				
CVCL_3019	Marcus	8	61.54%	X	12,15			11,12	9,11		11	12,13				
CVCL_2558	XP6BR LCL	8	61.54%	X,Y	10,12			11,12	8,12		11,12	9,13				
CVCL_RZ74	XYFMGC07K	8	61.54%	X,Y	10,12			11,13	10,11		11,13	12,13				
CVCL_V643	CHL-2	8	60.87%	X	10			11,12	11,13		8,11	13				
CVCL_1257	HCC1599 BL	8	60.87%	X	12,13			12	10,11		8,11	12,13				
CVCL_3317	HMCB	8	60.87%	X	10			11,12	11,13		8,11	13				
CVCL_0731	Hs 364.Ct	8	60.87%	X,Y	12			12	11,12		11	10,13				
CVCL_0803	Hs 573.Lu	8	60.87%	X	12			12,13	11,12		10,11	9,12				
CVCL_0804	Hs 573.T	8	60.87%	X,Y	12			12,13	11,12		10,11	9,12				
CVCL_1340	KP-N-SI9s	8	60.87%	X,Y	10			13	8,11		11,14	9,13				
CVCL_1T57	PCI-22B	8	60.87%	X	10,12			10,12	10,11		12	9				
CVCL_G366	SKR1	8	60.87%	X	10,12			10,12	10,11		12	9				
CVCL_IR64	U3019MG	8	60.87%	X	10,12		17,19	12	9,11	13,14	14	10	13,14	14	31.2,32.2	18,
CVCL_0207 *Best*	CCRF-CEM	9	60.00%	X	10,11	24	14,15	12,13	9,12	12,13	11	10,13	13,18	14,15	30,33.2	23,
CVCL_0207 *Worst*	CCRF-CEM	9	50.00%	X	11	24	15,16	12,13	9,13	13	10,11,12	10,13,14	13,18	14,15	30,33.2	23,
CVCL_A5RK	CD#59 ipsC	9	60.00%	X	11,12			10,12	9,11		8,11	11,12			30,31.2	
CVCL_VG71	CGBC 01	9	60.00%	X	10,13	23,24	15,16	9,12	9,11	13,14	8,9	11,13	13,14	14	30,32.2	23,
CVCL_0P14	GM00059	9	60.00%	X	11,12			10,12	9,11		8,11	11,12			30,31.2	
CVCL_B187	HUES 53	9	60.00%	X,Y	12,13	17,23	15,16	11,12	11,12	12,14	11,12	11,12	15,20	14	30,31.2	21,
CVCL_UN03	ICCSICi006-A	9	60.00%	X,Y	10,12			11,12	11,12		8,11	9,13			30,31.2	
CVCL_VX34	OCC-06	9	60.00%	X,Y	10,12	23,25	16,17	12	8,12	10,12	11,12	9,13	15,23	12,14	28,30	24
CVCL_5216	PMC42-LA	9	60.00%	X	10,12		15	11,12	7,9	13	11,14	9,11	14,17		30,31.2	20
CVCL_4020	SK-RC-35	9	60.00%	X	11,12	23,24	17	12	8,9	11,13,14	11,12	10,13	12,19	14	30,32.2	21,
CVCL_5133	HCC2450	8	60.00%	X,Y	13			12	9		11	11,13				

SIB Swiss Institute of Bioinformatics | Disclaimer

Article: Robin T, Capes-Davis A, Bairo

TOP
